# Synthesis of Tetramic Acid Fragments Derived from Vancoresmycin Showing Inhibitory Effects towards *S. aureus*

**DOI:** 10.1002/cmdc.202000241

**Published:** 2020-06-26

**Authors:** Lukas Martin Wingen, Marvin Rausch, Tanja Schneider, Dirk Menche

**Affiliations:** ^1^ Kekulé-Institut für Organische Chemie und Biochemie Rheinische Friedrich-Wilhelms-Universität Bonn Gerhard-Domagk-Str. 1 53121 Bonn Germany; ^2^ Institut für Pharmazeutische Mikrobiologie Rheinische Friedrich-Wilhelms-Universität Bonn Meckenheimer Allee 168 53115 Bonn Germany; ^3^ German Center for Infection Research (DZIF) Partner site Bonn–Cologne Bonn Germany

**Keywords:** antibiotics, tautomerism, tetramic acids, vancoresmycin

## Abstract

An efficient route to various vancoresmycin‐type tetramic acids has been developed. The modular route is based on an effective Fries‐type rearrangement to introduce various appending acetyl residues. The minimum inhibitory concentration (MIC) values of the new tetramic acids against *Staphylococcus aureus* and *Escherichia coli* were determined, revealing that three of the new compounds exhibit antimicrobial activity against *S. aureus*. These bioactive compounds were structurally most closely related to the authentic vancoresmycin building block. Additionally, the compounds induced a *lial*‐*lux* bioreporter, which responds to cell wall stress induced by antibiotics that interfere with the lipid II biosynthesis cycle. These data suggest the tetramic acid moiety to be a part of the vancoresmycin pharmacophore.

## Graphical abstract

**An efficient procedure** for the synthesis of various tetramic acid fragments related to vancoresmycin, a highly potent polyketide antibiotic, is reported. Activities against *S. aureus* and results of the *lial‐lux* bioreporter assay suggest the tetramic acid moiety to be part of the pharmacophore of this complex polyketide.

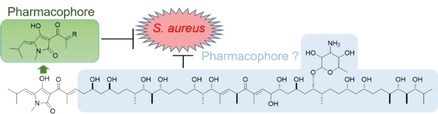

## Introduction

**Figure 1:**
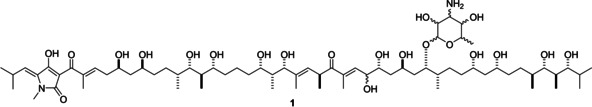
Structure of vancoresmycin (**1**) with the proposed stereoinformation.^2^

Vancoresmycin (**1**, Figure 1) presents a structurally unique linear metabolite that is characterized by a functionalized acetyl tetramic acid connected to an extended polyketide chain with a plethora of hydroxy‐ and methyl‐bearing stereogenic centers and also contains an attached aminoglycoside moiety. It was first isolated in 2002 from the fermentation broth of the actinomycete *Amycolatopsis* sp. ST 101170.^1^ This polyketide shows extremely potent MICs values ranging from 0.125 to 2 μg/mL against a variety of pathogens, including multiresistant Gram‐positive bacteria. In 2017, the mode of action was investigated, and it was proposed that vancoresmycin is involved in a non‐pore‐forming, concentration‐dependent depolarization of bacterial membranes.^2^

**Scheme 1:**
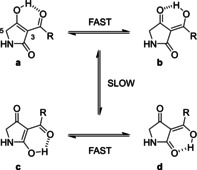
Tautomerism of 3‐acyltetramic acids.

Additionally, a stereochemical assignment based on domain analysis of the ketoreductase domains was proposed for most of the configurations, but has not yet been proven.^2^ As part of our studies with potent polyketide antibiotics bearing extended polyene segments,^3^‐^7^ we became interested in developing a synthetic route to the vancoresmycin‐type acetylated tetramic acids bearing characteristic labile diene segments and to evaluate their antibacterial properties. In general, tetramic acids including their 3‐acyl derivatives found in a variety of terrestrial and marine species have been studied and reviewed previously.^8^, ^9^ A range of bioactivities like antibacterial,^10^ antiviral,^11^ and antitumoral^12^ potencies have been reported for these compounds. Their intriguing structures enables complex formation with various metal ions like Mg^2+^, Fe^2+^, Zn^2+^ and Cu^2+[13]^ and in some cases this type of chelation has been shown to be essential for the biological activity.^14^ Furthermore, these features are characterized by extended tautomerism. As shown in Scheme 1, 3‐acyltetramic acids may occur in solution in four forms (**a**, **b**, **c**, **d**), which may be classified as a pair of “external” (**ab**/**cd**) and a pair of “internal” (**a**/**b**; **c**/**d**) tautomers. Due to the C−C bond rotation of the 3‐acyl group, the interconversion between the external isomers is a rather slow process on the NMR timescale, resulting in separate NMR signals in nonpolar solvents (e. g. CD_2_Cl_2_).^15^, ^16^ Complexation as well as tautomerism render tetramic acids fascinating heterocycles but also complicate synthesis and analysis.^8^, ^9^

## Results and Discussion

**Scheme 2:**
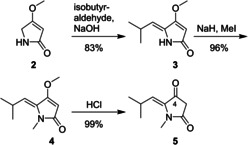
Synthesis of compound **5**.

As shown in Scheme 2, our successful route to vancoresmycin‐type tetramic acids started with introduction of the 5‐substituent to commercially available 4‐methoxy‐3‐pyrrolin‐2‐on (**2**). As previously reported, isobutyraldehyde was added in basic conditions to give compound **3** in high yield.^17^ After methylation of the ring nitrogen with iodomethane, the methoxy group was cleaved under acidic conditions yielding compound **5** in 79 % over three steps, without the need of column chromatography. If required, any intermediate may also be purified by using column chromatography.

**Scheme 3:**
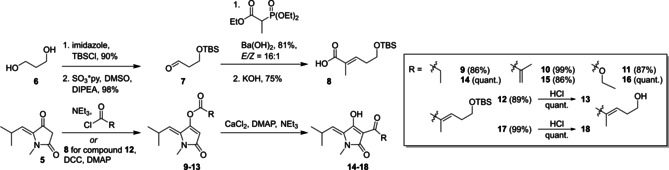
Synthetic route to vancoresmycin derived 3‐acyltetramic acids (**14**–**18**).

For the elaboration of structures **12**/**13** and **17**/**18** (Scheme 3), compound **8** was required. For synthesis of **8** a modification of a reported procedure from propanediol (**6**) was applied.^18^ In detail, a Parikh‐Doering oxidation was used instead of a Swern reaction and a different Horner‐Wadsworth‐Emmons (HWE) procedure was applied for better *E/Z* selectivity. Finally, saponification gave desired **8**, which was used for coupling with compound **5**. Various esters at the 4‐position of compound **5** are easily accessible using acid chlorides or esterification reactions like the Steglich protocol using *N,N’*‐dicyclohexylcarbodiimide (DCC) and 4‐(dimethyl‐amino)‐pyridine (DMAP) giving compounds **9**–**12** (Scheme 3) in good to excellent yields.^15^, ^17^ To realize the required oxygen‐to‐carbon transfer, the novel tetramic acid esters **9**–**13** were submitted to a known rearrangement by using CaCl_2_ in the presence of DMAP and triethylamine.^19^, ^20^ This process efficiently realized the synthesis of 3‐acyl derivatives **14**–**18** in high yield. Acylation reagents were chosen to be authentic to vancoresmycin and also to allow further modifications like aldol condensation, cross metathesis or a nucleophilic attack of the ester at the 3‐position. The TBS protecting group of compounds **12** and **17** can be cleaved either at the ester stage to furnish **13** or at the final stage to give **18**. Notably, preparation of compound **18** represents the successful synthesis of a vancoresmycin fragment, which is also the largest part of the natural product that bears no chiral center.

**Scheme 4:**
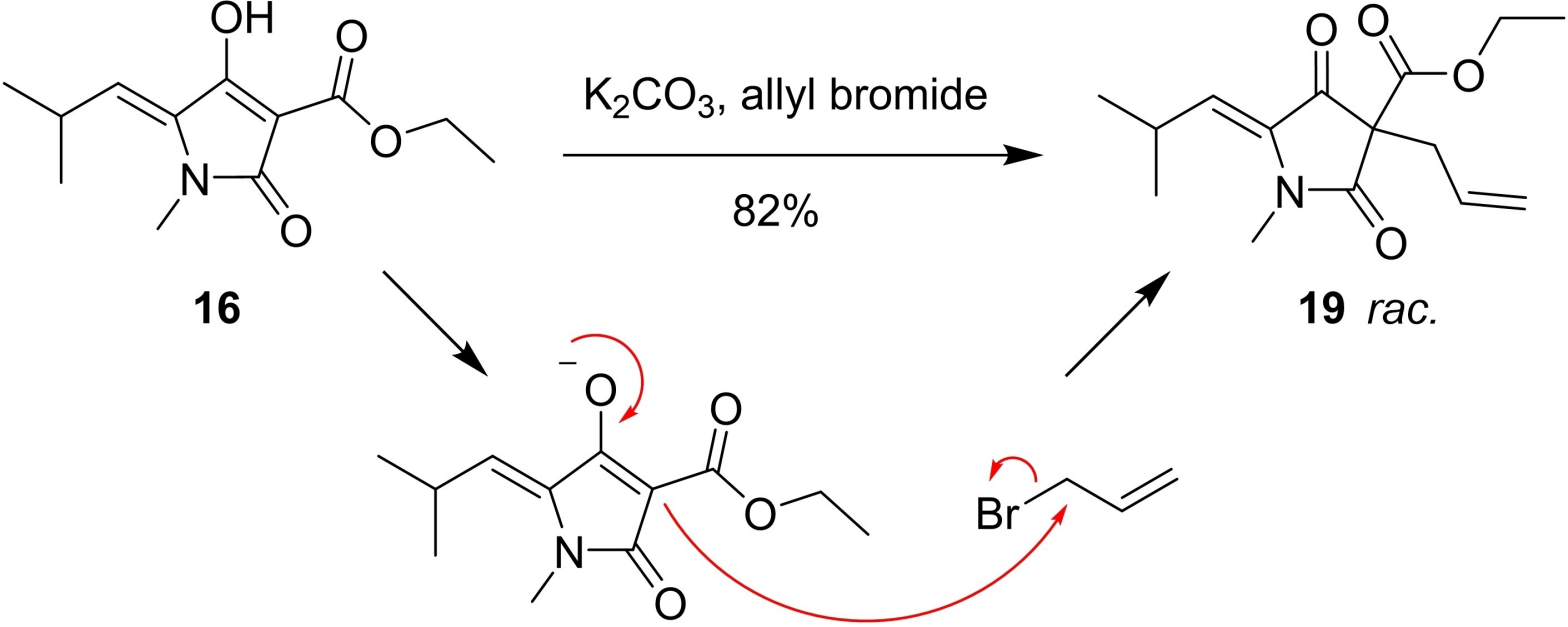
Synthesis of compound **19** and the proposed mechanism.

In CD_2_Cl_2_, tautomerism was observed for all tetramic acids **14**–**18**, resulting in double data sets of NMR measurements and also in broadening of the signals. For compound **15**, even more data sets could be observed in the NMR, due to the extended polyene system, emerging from the additional conjugated double bond. On the other hand, compound **16** showed only traces of a second data set, which indicates a stabilization of the double bond system by the introduced ester group. Several trials to protect and trap one of the tautomers of **14** as a silyl ether failed. Therefore, protection of **16** was evaluated as this representative is characterized by a lower degree of such isomerization. Here, an allylic protection was chosen as silyl enol ethers would be expected to be more labile. However, likewise the hydroxy group could not be protected in initial experiments. Instead, the allyl group was attached to the 3‐position yielding compound **19** as a racemic mixture, which was proven by HPLC on chiral phase (Scheme 4). Future investigations of suitable reaction conditions will show whether the alkylation may be tuned with respect to the oxygen to carbon regioselectivity.

In total, 14 tetramic acid derivatives, that is, **3**–**5** and **9–19**, have been synthesized with 13 not reported before. Only **3** has been described previously.^17^ For these compounds the MIC values against Gram‐positive and Gram‐negative bacteria, that is *E. coli* K12 and *S. aureus* SG511, were determined and summarized in Table 1.

As shown in Table 1, some of the synthesized tetramic acids demonstrated inhibitory effects against *S. aureus*. The more potent compounds were compounds **14**, **15** and **18**, which are also most similar as compared to vancoresmycin. Among these compounds **18** was a little less potent (MIC: 64 μg/mL) as compared to **15** (MIC=32 μg/mL), even though **18** is the longest and most authentic compound. A possible reason for this discrepancy may be due to lower degrees of stability, as evidenced by NMR studies showing that **18** is not stable in DMSO or acetonitrile. Additionally, **18** decomposes when stored over weeks (under argon, at −15 °C). No activities were observed against *E. coli*, which is in agreement with the reported data for vancoresmycin.^2^

**Table 1:** MIC values of the synthesized compounds.

	MIC [μg/mL]			MIC [μg/mL]	
	*E. coli* K12	*S. aureus* SG511		*E. coli* K12	*S. aureus* SG511
3	>128	>128	13	>128	128
4	>128	>128	14	>128	*64*
5	>128	128	15	>128	*32*
9	>128	128	16	>128	>128
10	>128	128	17	>128	>128
11	>128	>128	18	>128	*64*
12	>128	>128	19	>128	>128

**Figure 2:**
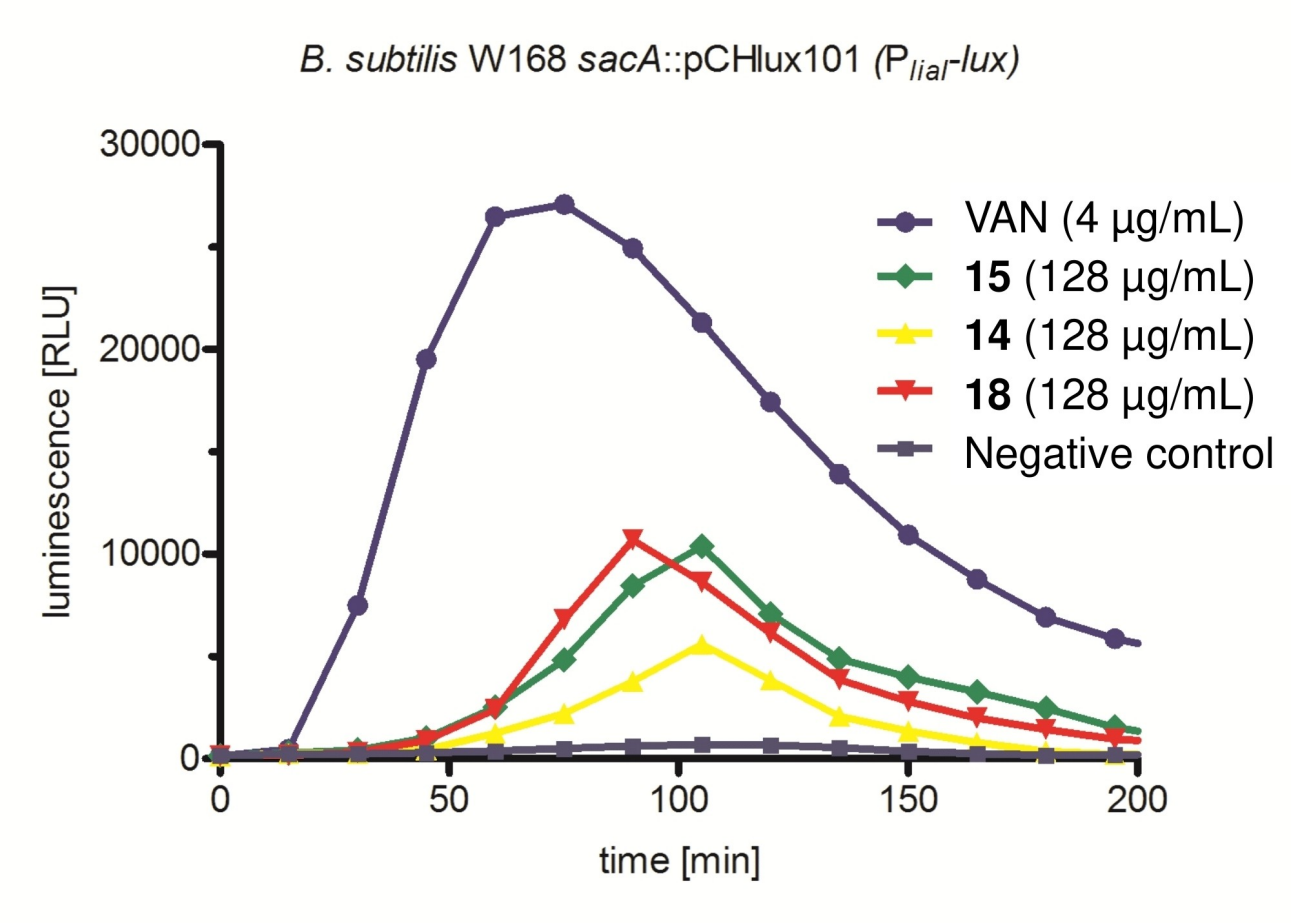
Results of the *lial‐lux* bioreporter assay. VAN=Vancomycin. In the negative control, no antibiotic compound was added to the assay.

To see if the synthesized compounds interact with the bacterial cell wall machinery they were further screened in a *lial*‐*lux* bioreporter assay, which responds to cell wall stress induced by antibiotics that interfere with the lipid II biosynthesis cycle substantiating again antimicrobial effects of the compounds, in line with the MIC values.^21^ As shown in Figure 2, **14**, **15** and **18** induce the *Bacillus subtilis lial‐lux* bioreporter, resulting in measurable luminescence and revealing interference with cell wall biosynthesis. A figure including the *lial‐lux* bioreporter assay outcome of additional novel tetramic acids can be found in the Supporting Information.

## Conclusion

In summary, we have reported an efficient synthetic route to novel vancoresmycin‐type 3‐acyl tetramic acids. The successful route uses an aldol condensation to efficiently attach the alkylidene substituent at C‐5 and a Fries‐type rearrangement to introduce the acetyl residue. Additionally, we determined the MIC values against *E. coli* and *S. aureus*, thereby identifying three compounds with antimicrobial activities against the Gram‐positive pathogen. Among all tested compounds the active heterocycles are structurally most closely related to vancoresmycin, thus indicating that this segment is part of the vancoresmycin pharmacophore. These structures are also characterized by high degrees of tautomerism, which might suggest that polyene shifts are important for biological activity. The active tetramic acids also induced the *B. subtilis lial‐lux* bioreporter, revealing their interference with cell wall biosynthesis.

## Conflict of interest

The authors declare no conflict of interest.

## Supporting information

As a service to our authors and readers, this journal provides supporting information supplied by the authors. Such materials are peer reviewed and may be re‐organized for online delivery, but are not copy‐edited or typeset. Technical support issues arising from supporting information (other than missing files) should be addressed to the authors.

Supplementary
