# Supplementary material for: Synthesis of Tetramic Acid Fragments Derived from Vancoresmycin Showing Inhibitory Effects towards S. aureus
Source: ChemMedChem. 2020 Jun 26;15(15):1390–3. doi: 10.1002/cmdc.202000241 (PMC7496136; doi:10.1002/cmdc.202000241)
Supplement: Supplementary file 1 — Supplementary [file CMDC-15-1390-s001.pdf]

ChemMedChem

Supporting Information

Synthesis of Tetramic Acid Fragments Derived from Vancoresmycin Showing Inhibitory Effects towards *S. aureus*

Lukas Martin Wingen, Marvin Rausch, Tanja Schneider, and Dirk Menche*

Supporting Information

Contents

1. General Methods S2
2. Experimental Details and Characterization Data S5
3. Copies of NMR Spectra S23

1. General Methods

Reaction conditions All reagents were purchased from commercial suppliers (Sigma-Aldrich, TCI, Acros, AlfaAesar) in the highest purity grade available and used without further purification. Anhydrous solvents (THF, DCM, MeCN, Et_2O, toluene) were obtained from a solvent drying system MB SPS-800 (MBraun) and stored over molecular sieves (3 or 4 Å). The reactions in which dry solvents were used were performed under an argon atmosphere in flame-dried glassware which had been flushed with argon unless stated otherwise. The reactants were handled using standard Schlenk techniques. Temperatures above rt (23 °C) refer to oil bath temperatures which were controlled by a temperature modulator. For cooling, the following baths were used: acetone/dry ice (−78 °C), MeCN/dry ice (−40 °C) and water/ice (0 °C).
TLC monitoring was performed with silica gel 60_{F254} pre-coated polyester sheets (0.2 mm silica gel, Macherey-Nagel) and visualized using UV light and staining with a solution of CAM (1.0 g $Ce(SO_4)_2)$, 2.5 g $(NH_4)_6Mo_7O_{24}$, 8 mL conc. H_2SO_4 in 100 mL H_2O) and subsequent heating. Purification methods for column chromatography, silica gel (pore size 60 Å, 40-63 µm) obtained from Merck or Aldrich was used. Compounds were eluated using the stated mixtures under a positive pressure of air. Solvents for column chromatography were distilled prior to use.

Analytical methods All NMR spectra were recorded on *Bruker* spectrometers at the University of Bonn under supervision of Dr. Senada Nozinovic with operating frequencies of 125 (^{13}C), 150 (^{13}C), 175 (^{13}C), 400 (^{1}H), 500 (^{1}H), 600 (^{1}H) and 700 MHz (^{1}H) in deuterated solvents obtained from *Deutero* and *Carl Roth*. Spectra were measured at room temperature unless stated otherwise and chemical shifts are reported in ppm relative to $(Me)_4Si$ (δ = 0.00 ppm) and were calibrated to the residual signal of undeuterated solvents.[1] Data for ^{1}H-NMR spectra are reported as follows: chemical shift (multiplicity, coupling constants, number of hydrogens, assignment). Abbreviations used are: s (singlet), d (doublet), t (triplet), q (quartet), quint (quintet), m (multiplet), br (broad).

Mass spectra (MS) and high-resolution-mass spectra (HRMS) were recorded on the documented systems in Table *S1* at the University of Bonn under supervision of Dr. Marianne Engeser.

Table *S1*: Used MS systems for MS and HRMS.

name	Manufacturer	measurements
MAT 95 XL	Thermo Finnigan (Bremen)	EI
MAT 90	Thermo Finnigan (Bremen)	EI, LIFDI, CI, FAB
MALDI autoflex II TOF/TOF	Bruker Daltonik (Bremen)	MALDI
micrOTOF-Q	Bruker Daltonik (Bremen)	ESI, APCI, nano-ESI, MS/MS, LC-MS, DC-MS
Orbitrap XL	Thermo Fisher Scientific (Bremen)	ESI, APCI, APPI, nano-ESI
Apex IV FT-ICR	Bruker Daltonik (Bremen)	ESI, nano-ESI, MALDI, EI, CI

Analytical HPLC analyses were performed on Knauer Wissenschaftliche Geräte GmbH systems by Andreas Schneider. The solvents for HPLC were purchased in HPLC grade. The chromatograms were recorded by UV-detection at 205, 230 and 300 nm.

Table *S2*: HPLC configuration for analytical HPLC.

	System
series	Azura
pumps	binary, HPG P 6.1L, 10 mL
pressure	700 bar
autosampler	3950 with 20 µL injection loop
mixing chamber	static, 350 µL
column heater	CT 6.1
detection type	DAD 6.1L, D_2/halogen lamps, 190-1000 nm
degasser	analytical 2-channel-online-degasser

Microbiological methods Minimum inhibitory concentration (MIC) was determined by broth microdilution according to CLSI guidelines. Cation adjusted Mueller-Hinton broth (MHB) was inoculated with $5x10^5$ CFU/ml at 37 °C for 16 h. MIC was taken as lowest compound concentration inhibiting visible growth. Experiments were performed in triplicate over three biological replicates.

Bacillus subtilis luciferase reporter assays were conducted as previously described.[2] Briefly, *Bacillus subtilis* W168 *sacA*::pCHlux101 (P_{liaI}-*lux*)[3] was grown in Mueller-Hinton broth at 30 °C containing 5 µg/ml chloramphenicol until they reached an OD_{600} of 0.5. Cells were added to 96-well white wall chimney plates containing antibiotics and luminescence measurements were performed at 30 °C in a microplate reader Spark 10M (Tecan). Experiments were performed with biological replicates. Data analysis was performed using Graph Pad Prism 5.01.

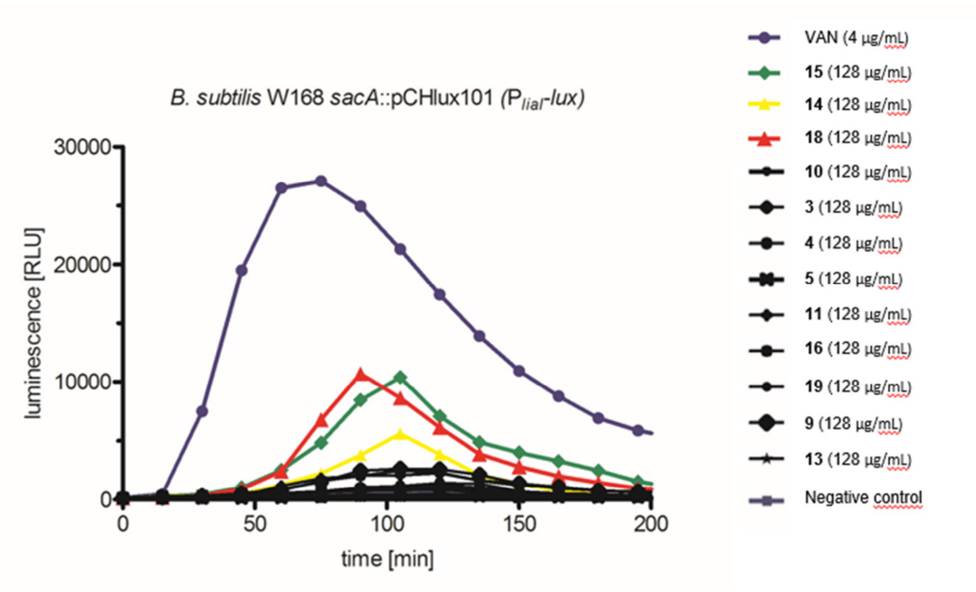

Figure S1: Additional results of the *liaI-lux* bioreporter assay. VAN = Vancomycin. In the negative control no antibiotic compound was added to the assay

2. Experimental Details and Characterization Data

Synthesis of Compound 3

(*Z*)-4-Methoxy-5-(2-methylpropylidene)-1,5-dihydro-2*H*-pyrrol-2-one

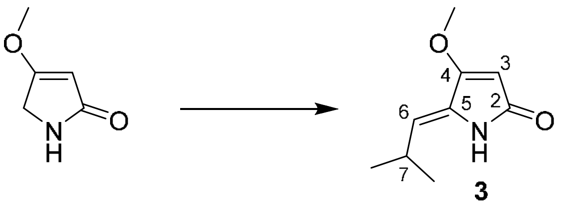

3

$C_9H_{13}NO_2$
M = 167.21 g/mol

To a solution of 4-methoxy-3-pyrrolin-2-on (3.00 g, 26.5 mmol, 1.00 equiv) in aq. NaOH (30 mL, 1 M) was added isobutyraldehyde (2.42 mL, 26.5 mmol, 1.00 equiv). The reaction mixture was stirred for 7 h at 60 °C. The formed precipitate was filtered, washed with cold water, redissolved in DCM, dried over Na_2SO_4 and the solvent was removed under reduced pressure to yield **3** as a white solid (3.70 g, 22.1 mmol, 83%).
R_f 0.14 (50% EtOAc/CyH). **^{1}H NMR** (700 MHz, CD_2Cl_2) δ = 7.35 (s, 1H, NH), 5.25 (d, *J* = 9.9 Hz, 1H, H-6), 5.07 (d, *J* = 1.7 Hz, 1H, H-3), 3.82 (s, 3H, O-CH_3), 2.56 – 2.50 (m, 1H, H-7), 1.08 (d, *J* = 6.7 Hz, 6H, 7-$(CH_3)_2$). **^{13}C NMR** (176 MHz, CD_2Cl_2) δ = 171.5 (C-2), 167.2 (C-4), 131.7 (C-5), 116.9 (C-6), 93.2 (C-3), 58.6 (O-CH_3), 27.7 (C-7), 23.0 (CH($\underline{C}H_3$)$_2$). **HRMS (ESI)** *m/z*: calcd for $C_9H_{13}NO_2H$ [M + H]$^+$: 168.1019, found: 168.1017. The spectroscopic data were in agreement with those previously reported.[4]

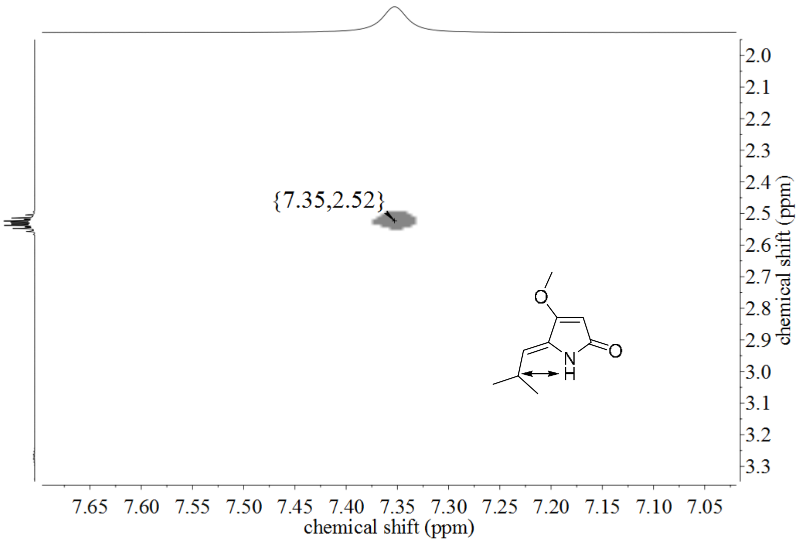

Figure S2: Selected NOESY signal of compound **3**, which was used for the determination of the *Z* configuration.

Synthesis of Compound 4

(*Z*)-4-Methoxy-1-methyl-5-(2-methylpropylidene)-1,5-dihydro-2*H*-pyrrol-2-one

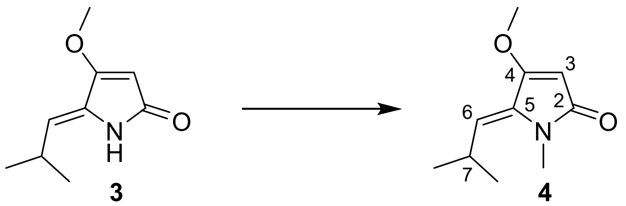

$C_{10}H_{15}NO_2$
M = 181.24 g/mol

A solution of compound **3** (3.50 g, 20.9 mmol, 1.00 equiv) in DMF (30 mL) was cooled to 0 °C and NaH (921 mg, 23.0 mmol, 1.10 equiv, 60% dispersion in mineral oil) was added. The reaction mixture was stirred 15 min at rt before cooling again to 0 °C. Iodomethane (1.43 mL, 23.0 mmol, 1.10 equiv) was added and the mixture was stirred 3 h at rt. Water (20 mL) and aq. sat. NH_4Cl solution (10 mL) were added. After separation of the organic phase, the aqueous phase was extracted with DCM (3x30 mL). The combined organic layers were washed with water (30 mL) and brine (30 mL), dried over

Na_2SO_4 and the solvent was removed under reduced pressure. The crude product was purified by flash chromatography (50% EtOAc/CyH) to yield the title compound **4** as a colorless solid (3.66 g, 20.2 mmol, 97%).

R*f* 0.24 (50% EtOAc/CyH). **^{1}H NMR** (500 MHz, CD_2Cl_2) δ = 5.23 (d, *J* = 10.7 Hz, 1H, H-6), 5.02 (s, 1H, H-3), 3.79 (s, 3H, O-CH_3), 3.22 (s, 3H, N-CH_3), 3.15 – 2.99 (m, 1H, H-7), 1.09 (d, *J* = 6.6 Hz, 6H, 7-$(CH_3)_2$). **^{13}C NMR** (126 MHz, CD_2Cl_2) δ = 170.7 (C-2), 166.8 (C-4), 132.9 (C-5), 117.7 (C-6), 91.5 (C-3), 58.4 (O-CH_3), 28.0 (N-CH_3), 26.1 (C-7), 24.1 (CH($\underline{C}H_3)_2$). **HRMS (ESI)** *m/z*: calcd for $C_{10}H_{15}NO_2H$ [M + H]$^+$: 182.1176, found: 182.1175.

Synthesis of Compound 5

(*Z*)-1-Methyl-5-(2-methylpropylidene)pyrrolidine-2,4-dione

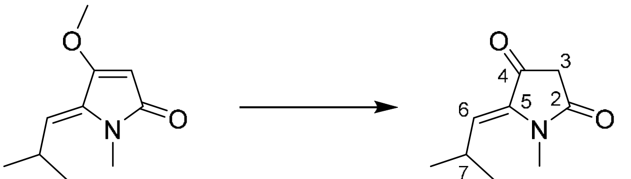

4 **5**

$C_9H_{12}NO_2$
M = 166.20 g/mol

A solution of compound **4** (50.0 mg, 276 µmol, 1.00 equiv) in conc. HCl (1.5 mL) was stirred at rt for 8 h. The reaction mixture was cooled to 0 °C and diluted with water (20 mL). The aqueous layer was extracted with DCM (3x20 mL), dried over Na_2SO_4 and the solvent was removed under reduced pressure to yield **5** as a yellow solid (45.8 mg, 274 µmol, 99%). If necessary, the product can be further purified by flash chromatography (50% EtOAc/CyH).

R*f* 0.32 (50% EtOAc/CyH). **^{1}H NMR** (700 MHz, CD_2Cl_2) δ = 5.01 (d, *J* = 9.9 Hz, 1H, H-6), 3.67 – 3.57 (m, 1H, H-7), 3.01 (s, 3H, N-CH_3), 3.01 (s, 2H, H-3), 1.04 (d, *J* = 6.7 Hz, 6H, 7-$(CH_3)_2$). **^{13}C NMR** (176 MHz, CD_2Cl_2) δ = 194.9 (C-4), 168.0 (C-2), 135.7 (C-5), 123.7 (C-6), 41.5 (C-3), 26.2 (N-CH_3), 25.7 (C-7), 23.6 (CH($\underline{C}H_3)_2$). **HRMS (ESI)** *m/z*: calcd for $C_9H_{13}NO_2H$ [M + H]$^+$: 168.1019, found: 168.1017.

General procedure for acylations using acid chlorides (9, 10, 11)

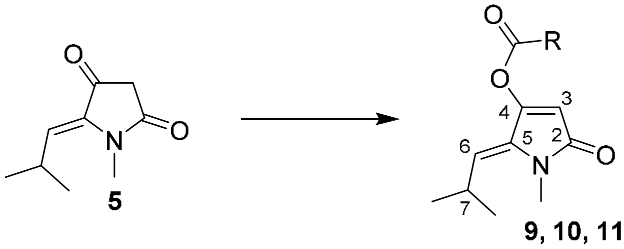

5 **9, 10, 11**

A solution of compound **5** (1.00 equiv) in DCM (0.25 M) was cooled to –5 °C. The appropriate acid chloride (1.15 equiv) and NEt_3 (1.40 equiv) were added dropwise. The reaction mixture was stirred for 30 min at –5 °C. 5% aq. $NaHCO_3$ solution and water were added followed by the extraction of the aqueous layer with DCM. The combined organic phases were washed with aq. HCl (0.5 M), dried over Na_2SO_4 and the solvent was removed under reduced pressure. The crude product was purified by flash chromatography (30% EtOAc/CyH).

Synthesis of Compound 9

(*Z*)-1-Methyl-2-(2-methylpropylidene)-5-oxo-2,5-dihydro-1*H*-pyrrol-3-yl propionate

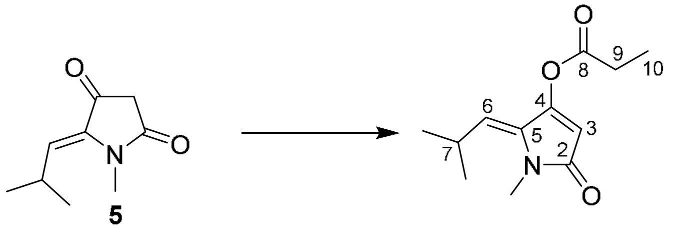

5

9
$C_{12}H_{17}NO_3$
M = 223.27 g/mol

Yield/Appearance: 86%/yellow solid. **R*f*** 0.22 (30% EtOAc/CyH). **^{1}H NMR** (500 MHz, CD_2Cl_2) δ = 6.12 (d, *J* = 1.4 Hz, 1H, H-3), 5.21 (dd, *J* = 10.0, 1.4 Hz, 1H, H-6), 3.31 – 3.20 (m, 1H, H-7), 3.00 (s, 3H, N-CH_3), 2.59 (q, *J* = 7.5 Hz, 2H, H-9), 1.23 (t, *J* = 7.5 Hz, 3H, H-10), 1.10 (d, *J* = 6.7 Hz, 6H, 7-$(CH_3)_2$). **^{13}C NMR** (126 MHz, CD_2Cl_2) δ = 170.6 (C-8), 168.0 (C-2), 154.9 (C-4), 133.6 (C-5), 123.0 (C-6), 108.2 (C-3), 28.7 (C-9), 26.8 (C-7), 25.2 (N-CH_3), 24.1 ($CH(\underline{C}H_3)_2$), 9.0 (C-10). **HRMS (ESI)** *m/z*: calcd for $C_{12}H_{17}NO_3H$ $[M + H]^+$: 224.1281, found: 224.1280.

Synthesis of Compound 10

(*Z*)-1-Methyl-2-(2-methylpropylidene)-5-oxo-2,5-dihydro-1*H*-pyrrol-3-yl methacrylate

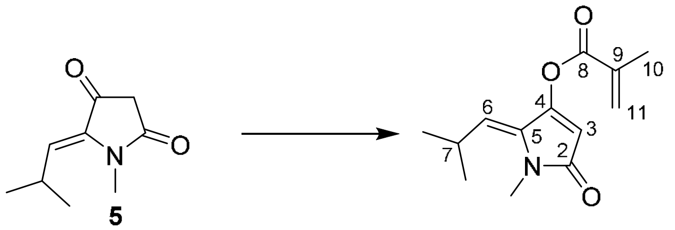

5

10
$C_{12}H_{17}NO_3$
M = 235.28 g/mol

Yield/Appearance: 99%/yellow solid. **R*f*** 0.21 (30% EtOAc/CyH). **^{1}H NMR** (500 MHz, CD_2Cl_2) δ = 6.29 – 6.26 (m, 1H, H-11), 6.17 (d, *J* = 1.4 Hz, 1H, H-3), 5.86 – 5.83 (m, 1H, H-11), 5.24 (dd, *J* = 10.1, 1.4 Hz, 1H, H-6), 3.32 – 3.20 (m, 1H, H-7), 3.02 (s, 3H, N-CH_3), 2.05 (s, 3H, H-10), 1.10 (d, *J* = 6.7 Hz, 6H, 7-$(CH_3)_2$). **^{13}C NMR** (126 MHz, CD_2Cl_2) δ = 167.9 (C-2), 163.8 (C-8), 154.9 (C-4), 135.8 (C-9), 133.7 (C-5), 129.0 (C-11), 122.9 (C-6), 108.6 (C-3), 26.8 (C-7), 25.2 (N-CH_3), 24.1 ($CH(\underline{C}H_3)_2$), 18.6 (C-10). **HRMS (ESI)** *m/z*: calcd for $C_{13}H_{17}NO_3H$ $[M + H]^+$: 236.1281, found: 236.1282.

Synthesis of Compound 11

(*Z*)-1-Methyl-2-(2-methylpropylidene)-5-oxo-2,5-dihydro-1*H*-pyrrol-3-yl methacrylate

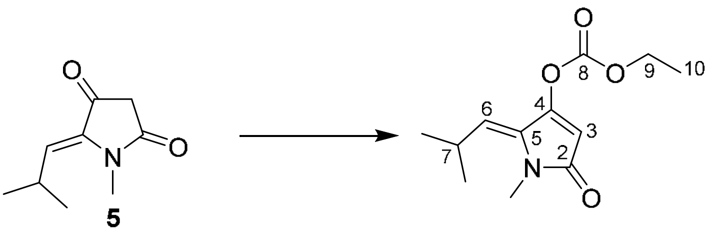

5

11
$C_{12}H_{17}NO_3$
M = 239.27 g/mol

Yield/Appearance: 87%/yellow solid. **R$_f$** 0.17 (30% EtOAc/CyH). **^{1}H NMR** (700 MHz, CD_2Cl_2) δ = 6.09 (d, *J* = 1.4 Hz, 1H, H-3), 5.22 (dd, *J* = 10.2, 1.4 Hz, 1H, H-6), 4.34 (q, *J* = 7.1 Hz, 2H, H-9), 3.31 – 3.21 (m, 1H, H-7), 3.00 (s, 3H, N-CH_3), 1.38 (t, *J* = 7.1 Hz, 3H, H-10), 1.09 (d, *J* = 6.6 Hz, 6H, 7-$(CH_3)_2$). **^{13}C NMR** (176 MHz, CD_2Cl_2) δ = 167.5 (C-2), 155.5 (C-4), 151.5 (C-8), 133.0 (C-5), 123.5 (C-6), 107.4 (C-3), 66.4 (C-9), 26.7 (C-7), 25.2 (N-CH_3), 24.1 (CH($\underline{C}H_3)_2$), 14.5 (C-10). **HRMS (ESI)** *m/z*: calcd for $C_{12}H_{17}NO_4H$ $[M + H]^+$: 240.1230, found: 240.1228.

Synthesis of Compound 7.I

3-((*tert*-Butyldimethylsilyl)oxy)propan-1-ol

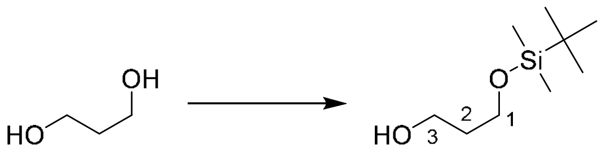

7.I
$C_9H_{22}O_2Si$
M = 190.36 g/mol

A solution of propanediol (7.21 mL, 99.5 mmol, 5.00 equiv) and 1*H*-imidazole (1.49 g, 21.9 mmol, 1.10 equiv) in THF (40 mL) was cooled to 0 °C. *tert*-Butyldimethylsilyl chloride (3.00 g, 19.9 mmol, 1.00 equiv) was added and the reaction mixture was stirred 30 min at 0 °C and on at rt. sat. aq. NH_4Cl solution (20 mL) was added and the aqueous phase was extracted with Et_2O (3x60 mL). The combined organic phase was washed with water (30 mL), dried over $MgSO_4$ and the solvent was removed under reduced pressure. The crude product was purified by flash chromatography (30% EtOAc/CyH) to yield the title compound **7.I** as a colorless oil (3.40 g, 17.9 mmol, 90%).

R$_f$ 0.34 (30% EtOAc/CyH). **^{1}H NMR** (500 MHz, $CDCl_3$) δ = 3.83 (t, *J* = 5.7 Hz, 2H, H-1), 3.80 (t, *J* = 5.7 Hz, 2H, H-3), 2.07 (s, 1H, OH), 1.78 (p, 2H, H-2), 0.90 (s, 9H, TBS), 0.08 (s, 6H, TBS). **^{13}C NMR** (126 MHz, $CDCl_3$) δ = 63.1 (C-1), 62.6 (C-3), 34.3 (C-2), 26.0 (TBS), 18.3 (TBS), -5.4 (TBS). **MS (EI)** *m/z*: calcd for $C_5H_{13}O_2Si^{+\bullet}$ $[M - {}^tBu]^{+\bullet}$: 133.0, found: 133.0. The spectroscopic data were in agreement with those previously reported.[5]

Synthesis of Compound 7

3-((*tert*-Butyldimethylsilyl)oxy)propanal

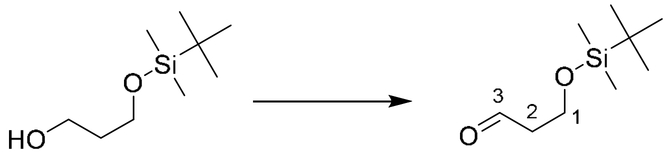

7.I **7**

$C_9H_{20}O_2Si$
M = 188.34 g/mol

DCM (45 mL) was added to sulfur trioxide pyridine complex (8.78 g, 55.2 mmol, 3.00 equiv) followed by the addition of *N*,*N*-diisopropylethylamin (12.5 mL, 73.6 mmol, 4.00 equiv) and DMSO (13.1 mL, 164 mmol, 10.0 equiv). The reaction mixture was cooled to 0 °C and alcohol **7.I** (3.50 g, 18.4 mmol, 1.00 equiv) was added. After stirring at rt for 1.5 h, sat. aq. $NaHCO_3$ solution (150 mL) was added and the aqueous phase was extracted with DCM (3x150 mL). The combined organic phase was washed with $CuSO_4$, NH_4Cl and brine solution (each aq. sat. 2x150 mL). The organic phase was dried over $MgSO_4$ and the solvent was removed under reduced pressure to yield the title compound **7** as a colorless oil (3.40 g, 18.0 mmol, 98%), which was used without further purification.

$\mathbf{R_f}$ 0.41 (20% EtOAc/CyH). **^{1}H NMR** (500 MHz, $CDCl_3$) δ = 9.80 (t, *J* = 2.1 Hz, 1H, H-3), 3.98 (t, *J* = 6.0 Hz, 2H, H-1), 2.59 (td, *J* = 6.0, 2.1 Hz, 2H, H-2), 0.88 (s, 9H, TBS), 0.06 (s, 6H, TBS). **^{13}C NMR** (126 MHz, $CDCl_3$) δ = 202.2 (C-3), 57.6 (C-1), 46.7 (C-2), 26.0 (TBS), 18.4 (TBS), -5.3 (TBS). **MS (ESI)** *m/z*: calcd for $C_9H_{20}O_2SiH$ $[M + H]^+$: 189.131, found: 189.130. The spectroscopic data were in agreement with those previously reported.[6]

Synthesis of Compound 8.I

Ethyl (*E*)-5-((*tert*-butyldimethylsilyl)oxy)-2-methylpent-2-enoate

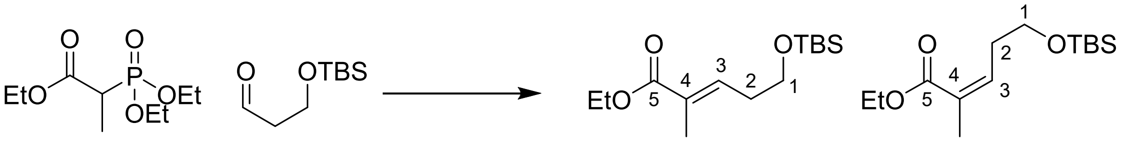

7 **8.I*E*** **8.I*Z***

$C_{14}H_{28}O_3Si$ $C_{14}H_{28}O_3Si$
M = 272.46 g/mol M = 272.46 g/mol

$Ba(OH)_2$ (1.37 g, 7.99 mmol, 3.50 equiv) was dried for 5 h at 120 °C using high vacuum. After cooling to rt, THF (40 mL) was added followed by the phosphonate (686 µL, 3.20 mmol, 1.40 equiv). The suspension was stirred for 30 min at rt. Freshly prepared aldehyde **7** (430 mg, 2.28 mmol, 1.00 equiv) was added and the reaction was stirred over night at rt. The Suspension was filtered over Celite and washed with Et_2O. The solvent was removed under reduced pressure and the crude was purified by flash chromatography (3% Et_2O /CyH) to yield the title compounds **8.I*E*** (472 mg, 1.73 mmol, 76%, *E*/*Z* = 16:1) and **8.I*Z*** (29.5 mg, 108 µmol, 5%, *E*/*Z* = 16:1) as colorless oils.

Analytical data for **8.I*E***:

R_f 0.22 (5% Et_2O /CyH). **^{1}H NMR** (700 MHz, $CDCl_3$) δ = 6.78 (tq, *J* = 6.8, 1.3 Hz 1H, H-3), 4.19 (q, *J* = 7.1 Hz, 2H, OEt), 3.70 (t, *J* = 6.7 Hz, 2H, H-1), 2.40 (dt, *J* = 7.2, 6.8 Hz, 2H, H-2), 1.85 (s, 3H, 4-CH_3), 1.29 (t, *J* = 7.1 Hz, 3H, OEt), 0.89 (s, 9H, TBS), 0.05 (s, 6H, TBS). **^{13}C NMR** (176 MHz, $CDCl_3$) δ = 168.2 (C-5), 138.6 (C-3), 129.4 (C-4), 61.9 (C-1), 60.6 (OEt), 32.5 (C-2), 26.0 (TBS), 18.5 (TBS), 14.4 (OEt), 12.7 (4-CH_3), -5.2 (TBS). **HRMS (ESI)** *m/z*: calcd for $C_{14}H_{28}O_3SiH$ $[M + H]^+$: 273.1880, found: 273.1880. The spectroscopic data were in agreement with those previously reported.[7]

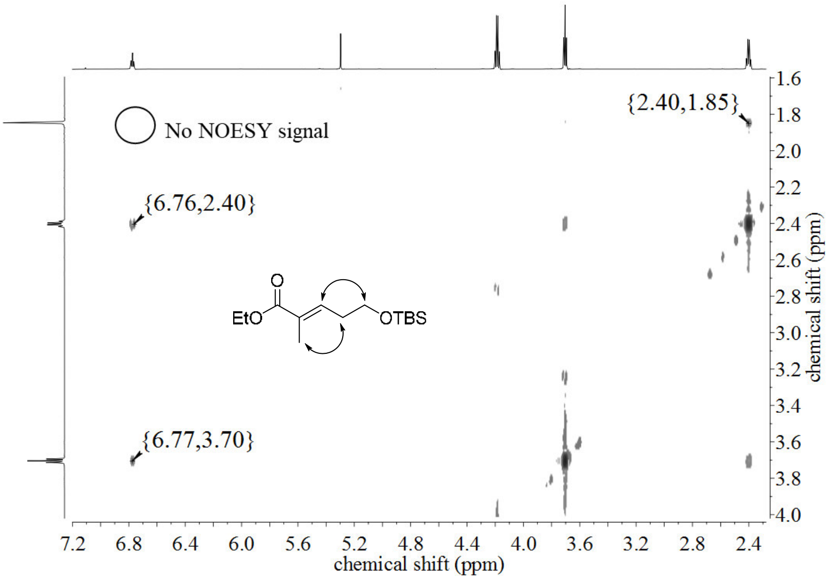

Figure S3: Selected NOESY signals of compound **8.I*E***, which were used for the determination of the *E* configuration. No NOESY signal could be detected between H-3 and 4-CH_3.

Analytical data for **8.I*Z***:

R_f 0.27 (5% Et_2O /CyH). **^{1}H NMR** (500 MHz, $CDCl_3$) δ = 6.01 (tq, *J* = 7.3, 1.6 Hz 1H, H-3), 4.19 (q, *J* = 7.1 Hz, 2H, OEt), 3.68 (t, *J* = 6.5 Hz, 2H, H-1), 2.71 – 2.64 (m, 2H, H-2), 1.91 (q, *J* = 1.6 Hz, 3H, 4-CH_3), 1.30 (t, *J* = 7.1 Hz, 3H, OEt), 0.89 (s, 9H, TBS), 0.05 (s, 6H, TBS). **^{13}C NMR** (126 MHz, $CDCl_3$) δ = 168.2 (C-5), 139.4 (C-3), 128.7 (C-4), 62.7 (C-1), 60.2 (OEt), 33.3 (C-2), 26.1 (TBS), 20.8 (4-CH_3), 18.5 (TBS), 14.4 (OEt), -5.1 (TBS). **HRMS (ESI)** *m/z*: calcd for $C_{14}H_{28}O_3SiH$ $[M + H]^+$: 273.1880, found: 273.1883. The spectroscopic data were in agreement with those previously reported.[7]

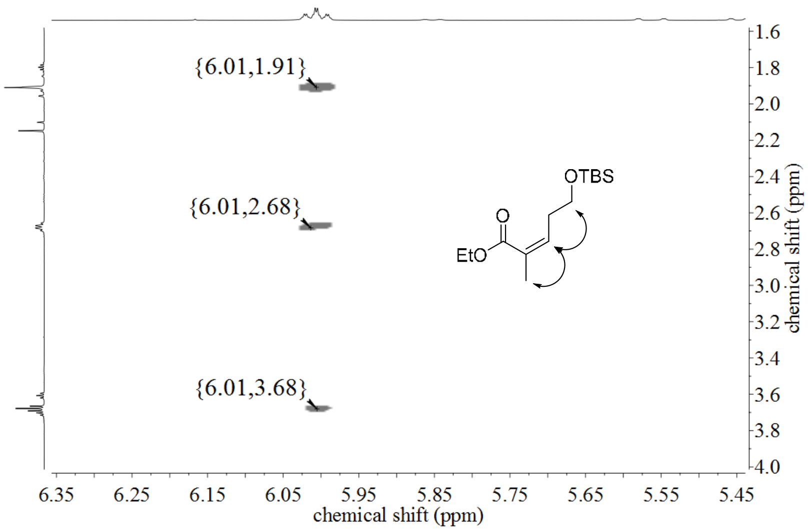

Figure S4: Selected NOESY signals of compound **8.I*Z***, which were used for the determination of the *Z* configuration.

Synthesis of Compound 8

(*E*)-5-((*tert*-Butyldimethylsilyl)oxy)-2-methylpent-2-enoic acid

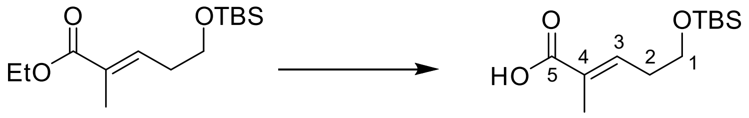

8.I*E* **8**

$C_{12}H_{24}O_3Si$
M = 244.41 g/mol

KOH (41.5 mg, 740 µmol, 1.20 equiv) was dissolved in water (40 µL) and ethanol (100 µL). The resulting solution was added to the ester **8.I*E*** (168 mg, 617 µmol, 1.00 equiv) and the reaction was stirred for 6 h at rt. Et_2O (10 mL) and water (10 mL) were added and the aqueous phase was acidified with aq. 1 M HCl until the pH turned 5-6. The organic layer was separated and the aqueous layer was extracted with Et_2O (3x20 mL). The combined organic phase was dried over $MgSO_4$, the solvent was removed under reduced pressure and the crude product was purified by flash chromatography (20% EtOAc/CyH) to yield the title compound **8** as a colorless oil (113 mg, 464 µmol, 75%).

R_f 0.37 (30% EtOAc/CyH). **^{1}H NMR** (500 MHz, $CDCl_3$) δ = 6.94 – 6.88 (m, 1H, H-3), 3.72 (t, *J* = 6.7 Hz, 2H, H-1), 2.43 (dt, *J* = 7.2 Hz, *J* = 6.7 Hz, 2H, H-2), 1.86 (s, 3H, 4-CH_3), 0.89 (s, 9H, TBS), 0.06 (s, 6H, TBS). **^{13}C NMR** (126 MHz, $CDCl_3$) δ = 172.7 (C-5), 141.7 (C-3), 128.6 (C-4), 61.7 (C-1), 32.8 (C-2), 26.0 (TBS), 18.4 (TBS), 12.4 (4-CH_3), -5.2 (TBS). **HRMS (ESI)** *m/z*: calcd for $C_{12}H_{24}O_3SiH$ $[M + H]^+$: 245.1567, found: 245.1563. The spectroscopic data were in agreement with those previously reported.[8]

Synthesis of Compound 12

(*Z*)-1-Methyl-2-(2-methylpropylidene)-5-oxo-2,5-dihydro-1*H*-pyrrol-3-yl (*E*)-5-((*tert*-butyldimethylsilyl)oxy)-2-methylpent-2-enoate

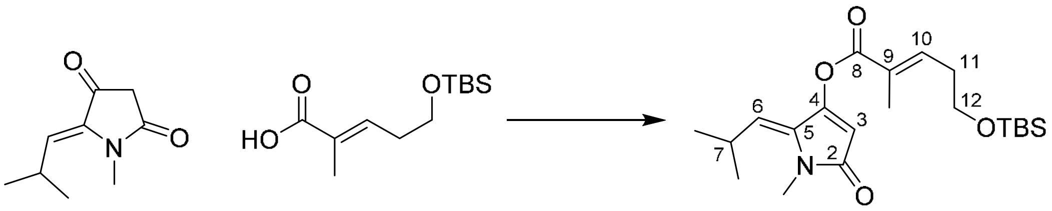

5 **8** **12**

$C_{21}H_{35}NO_4Si$
M = 393.60 g/mol

Acid **8** (439 mg, 1.79 mmol, 1.20 equiv) was dissolved in DCM (12 mL) and cooled to 0 °C. DCC (1.79 mL, 1.79 mmol, 1.20 equiv, 1 M in DCM) and DMAP (36.5 mg, 299 µmol, 0.20 equiv) were added and the reaction was stirred 5 min at 0 °C. Tetramic acid **5** (250 mg, 1.50 mmol, 1.00 equiv) was added and the reaction was allowed to warm to rt and stirred for 6 h. The resulting suspension was filtered over Celite, washed with EtOAc and the solvent was removed under reduced pressure. The crude product was purified by flash chromatography (20% EtOAc/CyH) to yield the title compound **12** as a yellow oil (522 mg, 1.33 mmol, 89%).

R_f 0.28 (30% EtOAc/CyH). **^{1}H NMR** (700 MHz, $CDCl_3$) δ = 7.00 – 6.97 (tq, *J* = 7.4, 1.3 Hz, 1H, H-10), 6.21 (d, *J* = 1.4 Hz, 1H, H-3), 5.20 (dd, *J* = 10.0, 1.3 Hz, 1H, H-6), 3.75 (t, *J* = 6.4 Hz, 1H, H-12), 3.27 – 3.21 (m, 1H, H-7), 3.06 (s, 3H, N-CH_3), 2.49 (dt, *J* = 7.4, 6.4 Hz, 2H, H-11), 1.95 (s, 3H, 9-CH_3), 1.11 (d, *J* = 6.7 Hz, 6H, 7-$(CH_3)_2$), 0.89 (s, 9H, TBS), 0.06 (s, 6H, TBS). **^{13}C NMR** (176 MHz, $CDCl_3$) δ = 168.0 (C-2), 163.9 (C-8), 154.6 (C-4), 143.4 (C-10), 133.6 (C-5), 128.2 (C-9), 122.5 (C-6), 108.6 (C-3), 61.6 (C-

12), 33.0 (C-11), 26.3 (C-7), 26.0 (TBS), 25.1 (N-CH_3), 24.1 (7-$(CH_3)_2$), 18.4 (TBS), 12.8 (9-CH_3), -5.2 (TBS). **HRMS (ESI)** *m/z*: calcd for $C_{21}H_{35}NO_4SiH$ [M + H]$^+$: 394.2408, found: 394.2415.

Synthesis of Compound 13

(*Z*)-1-Methyl-2-(2-methylpropylidene)-5-oxo-2,5-dihydro-1*H*-pyrrol-3-yl (*E*)-5-hydroxy-2-methylpent-2-enoate

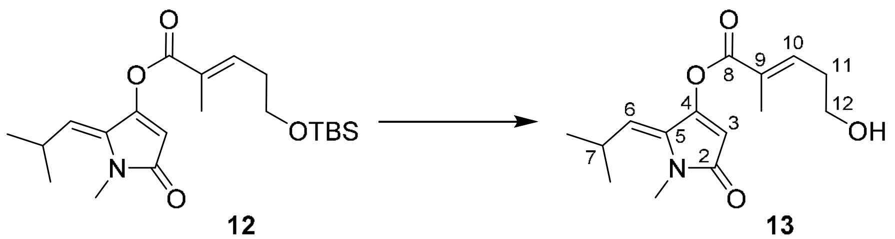

$C_{15}H_{21}NO_4$
M = 279.34 g/mol

Tetramic acid derivative **12** (40.0 mg, 102 µmol, 1.00 equiv) was dissolved in MeOH (2 mL) and 1 M aq. HCl (173 µL, 173 µmol, 1.70 equiv) was added dropwise at 0 °C. The reaction mixture was stirred for 1 h at rt. DCM (10 mL) and water (10 mL) were added and the aqueous layer was extracted with DCM (3x10 mL). The combined organic phase was dried over Na_2SO_4, the solvent was removed under reduced pressure and the crude product was purified by flash chromatography (80% EtOAc/CyH) to yield the title compound **13** as a white solid (28.3 mg, 101 µmol, quant.).

R_f 0.28 (80% EtOAc/CyH). **^{1}H NMR** (400 MHz, $CDCl_3$) δ = 7.02 (tq, *J* = 7.4, 1.3 Hz, 1H, H-10), 6.24 (d, *J* = 1.4 Hz, 1H, H-3), 5.21 (dd, *J* = 10.1, 1.4 Hz, 1H, H-6), 3.81 (dt, *J* = 5.9, 5.9 Hz, 2H, H-12), 3.35 – 3.18 (m, 1H, H-7), 3.05 (s, 3H, N-CH_3), 2.60 – 2.50 (m, 2H, H-11), 1.97 (q, *J* = 1.1 Hz, 3H, 9-CH_3), 1.62 (t, *J* = 5.4 Hz, 1H, OH), 1.11 (d, *J* = 6.7 Hz, 6H, 7-$(CH_3)_2$). **^{13}C NMR** (126 MHz, $CDCl_3$) δ = 168.0 (C-2), 163.7 (C-8), 154.5 (C-4), 142.7 (C-10), 133.5 (C-5), 128.7 (C-9), 122.6 (C-6), 108.4 (C-3), 61.3 (C-12), 32.5 (C-11), 26.4 (C-7), 25.1 (N-CH_3), 24.1 (7-$(CH_3)_2$), 12.9 (9-CH_3). **HRMS (ESI)** *m/z*: calcd for $C_{15}H_{21}NO_4H$ [M + H]$^+$: 280.1543, found: 280.1539.

General procedure for the O-to C-rearrangement (14, 15, 16, 17, 18)

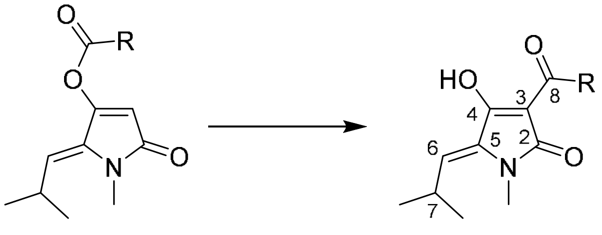

9, 10, 11, 12, 13 **14, 15, 16, 17, 18**

The corresponding ester (1.00 equiv) was dissolved in DCM (0.15 M). $CaCl_2$ (1.50 equiv) and DMAP (0.30 equiv) were added followed by NEt_3 (1.40 equiv). The reaction mixture was stirred for 4-7 h at rt until TLC showed complete conversion. DCM and aq. 1 M HCl were added and the aqueous layer was extracted with DCM. The combined organic phase was washed with water, dried over Na_2SO_4 and the solvent was removed under reduced pressure.

Synthesis of Compound 14

(3*E*,5*Z*)-3-(1-Hydroxypropylidene)-1-methyl-5-(2-methylpropylidene)pyrrolidine-2,4-dione

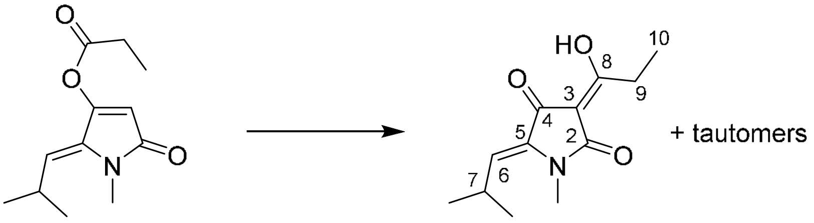

9 **14**

$C_{12}H_{17}NO_3$
M = 223.27 g/mol

Yield/Appearance: quant./orange solid. **R*f*** 0.24 (50% EtOAc/CyH). **^{1}H NMR** (500 MHz, CD_2Cl_2) δ = 13.6 (brs, 1H, OH), 5.25/5.13 (2xd, *J* = 10.1 Hz, 1H, H-6), 3.86 – 3.61/3.70 – 3.61 (2xm, 1H, H-7), 3.02/3.00 (2xs, 3H, N-CH_3), 2.92/2.87 (2xq, *J* = 7.5 Hz, 2H, H-9), 1.20/1.19 (2xt, 3H, H-10), 1.08/1.05 (d, *J* = 6.6 Hz, 6H, 7-$(CH_3)_2$). **^{13}C NMR** (126 MHz, CD_2Cl_2) δ = 194.5/187.6 (C-8), 186.4/183.0 (C-4), 171.3/164.5 (C-2), 134.8/132.9 (C-5), 125.9/124.9 (C-6), 104.6/101.9 (C-3), 28.5/26.3 (C-9), 26.3/25.3 (C-7), 25.2/25.1 (N-

CH_3), 23.7/23.6 (C-10), 10.3/9.6 (7-$(CH_3)_2$). **HRMS (ESI)** *m/z*: calcd for $C_{12}H_{17}NO_3H$ [M + H]$^+$: 224.1281, found: 224.1285.

Synthesis of Compound 15

(3*E*,5*Z*)-3-(1-Hydroxy-2-methylallylidene)-1-methyl-5-(2-methylpropylidene)pyrrolidine-2,4-dione

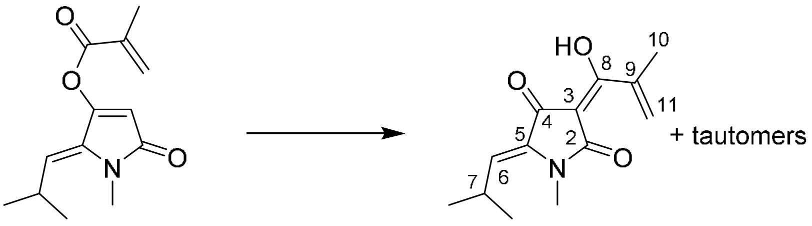

15

$C_{13}H_{17}NO_3$
M = 235.28 g/mol

Yield/Appearance: 86%/yellow solid. **R*f*** 0.27 (50% EtOAc/CyH). Due to extended tautomerism only signals of two major tautomers were picked. **^{1}H NMR** (700 MHz, CD_2Cl_2) δ = 6.44/6.40 (2xs, 1H, H-11), 5.85/5.79 (2xs, 1H, H-11), 5.26/5.16 (2xd, *J* = 9.9 Hz, 1H, H-6), 3.84 – 3.79/3.71 – 3.66 (2xm, 1H, H-7), 3.06/3.01 (2xs, 3H, N-CH_3), 2.04 (s, 3H, H-10), 1.10/1.05 (2xd, *J* = 6.7 Hz, 6H, 7-$(CH_3)_2$). **^{13}C NMR** (176 MHz, CD_2Cl_2) δ = 188.5/181.0 (C-8), 187.9/181.0 (C-4) 172.7/163.7 (C-2), 138.8/137.6 (C-9), 134.3/132.5 (C-5), 128.9/127.2 (C-11), 125.8/125.2 (C-6), 103.9/101.2 (C-3), 26.3/25.2 (C-7), 25.5/25.4 (N-CH_3), 23.7/23.5 (7-$(CH_3)_2$), 19.2/19.0 (H-10). **HRMS (ESI)** *m/z*: calcd for $C_{13}H_{17}NO_3H$ [M + H]$^+$: 236.1281, found: 236.1279.

Synthesis of Compound 16

Ethyl (*Z*)-4-hydroxy-1-methyl-5-(2-methylpropylidene)-2-oxo-2,5-dihydro-1*H*-pyrrole-3-carboxylate

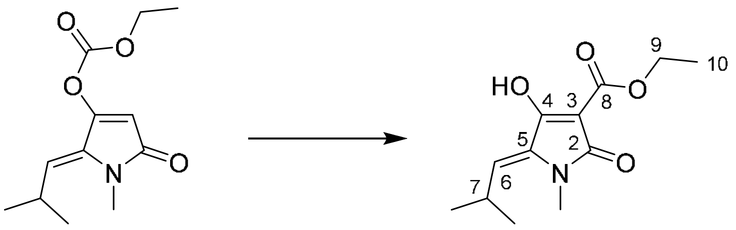

$C_{12}H_{17}NO_4$
M = 239.27 g/mol

Yield/Appearance: quant./red oil. **R*f*** 0.26 (50% EtOAc/CyH). **^{1}H NMR** (500 MHz, CD_2Cl_2) δ = 11.58 (brs, 1H, OH), 5.40 (d, *J* = 10.1 Hz, 1H, H-6), 4.37 (q, *J* = 7.1 Hz, 2H, H-9), 3.50 – 3.43 (m, 1H, H-7), 2.98 (s, 3H, N-CH_3), 1.37 (t, *J* = 7.1 Hz, 3H, H-10), 1.11 (d, *J* = 6.6 Hz, 6H, 7-$(CH_3)_2$). **^{13}C NMR** (126 MHz, CD_2Cl_2) δ = 175.2 (C-6), 168.5 (C-8), 163.4 (C-2), 131.5 (C-5), 127.3 (C-6), 98.2 (C-3), 61.9 C-9), 27.0 (C-7), 25.2 (N-CH_3), 23.8 (7-$(CH_3)_2$), 14.6 (C-10). **HRMS (ESI)** *m/z*: calcd for $C_{12}H_{17}NO_4H$ $[M + H]^+$: 240.1230, found: 240.1229.

Synthesis of Compound 17

(3*E*,5*Z*)-3-((*E*)-5-((*tert*-Butyldimethylsilyl)oxy)-1-hydroxy-2-methylpent-2-en-1-ylidene)-1-methyl-5-(2-methylpropylidene)pyrrolidine-2,4-dione

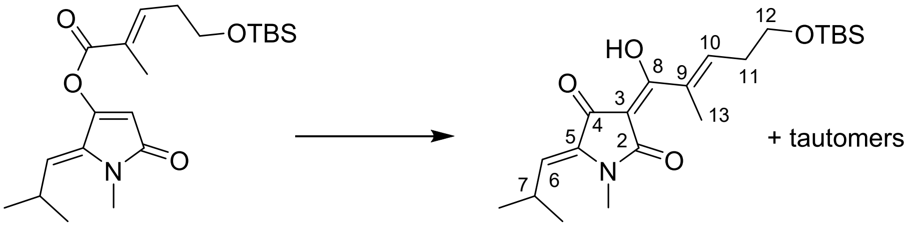

$C_{21}H_{35}NO_4Si$
M = 393.60 g/mol

Yield/Appearance: 99%/yellow oil. **R*f*** 0.18 (30% EtOAc/CyH). Due to extended tautomerism only signals of two major tautomers were picked. **^{1}H NMR** (500 MHz, CD_2Cl_2) δ = 7.00/6.98 (2xt, *J* = 7.2 Hz, 1H, H-10), 5.23/5.13 (d, *J* = 9.9 Hz, 1H, H-6), 3.87 – 3.79/3.73 – 3.64 (2xm, 1H, H-7), 3.77/3.75 (2xt, *J* = 6.7 Hz, 2H, H-12), 3.05/2.99 (2xs, 3H, N-CH_3), 2.52 (dt, *J* = 6.7, 6.6 Hz, 2H, H-11), 1.94/1.93 (2xs, 3H, H-13),

1.09/1.04 (2xd, *J* = 6.6 Hz, 6H, 7-(CH_3)$_2$), 0.89/0.88 (2xs, 9H, TBS), 0.06/0.06 (2xs, 6H, TBS). **^{13}C NMR** (126 MHz, CD_2Cl_2) δ = 189.0/182.7 (C-8), 188.4/180.9 (C-4), 173.1/163.9 (C-2), 143.7/141.8 (C-10), 134.4/132.7 (C-5), 131.9/130.5 (C-9), 125.2/124.7 (C-6), 103.4/100.3 (C-3), 62.1 (C-12), 33.5/33.2 (C-11), 26.2 (TBS), 26.2/25.2 (C-7), 25.5/25.3 (N-CH_3), 23.8/23.6 (7-(CH_3)$_2$), 18.7 (TBS), 13.2/13.1 (C-13), -5.1 (TBS). **HRMS (ESI)** *m/z*: calcd for $C_{21}H_{35}NO_4SiH$ [M + H]$^+$: 394.2408, found: 394.2406.

Synthesis of Compound 18

(3*E*,5*Z*)-3-((*E*)-1,5-Dihydroxy-2-methylpent-2-en-1-ylidene)-1-methyl-5-(2-methylpropylidene)pyrrolidine-2,4-dione

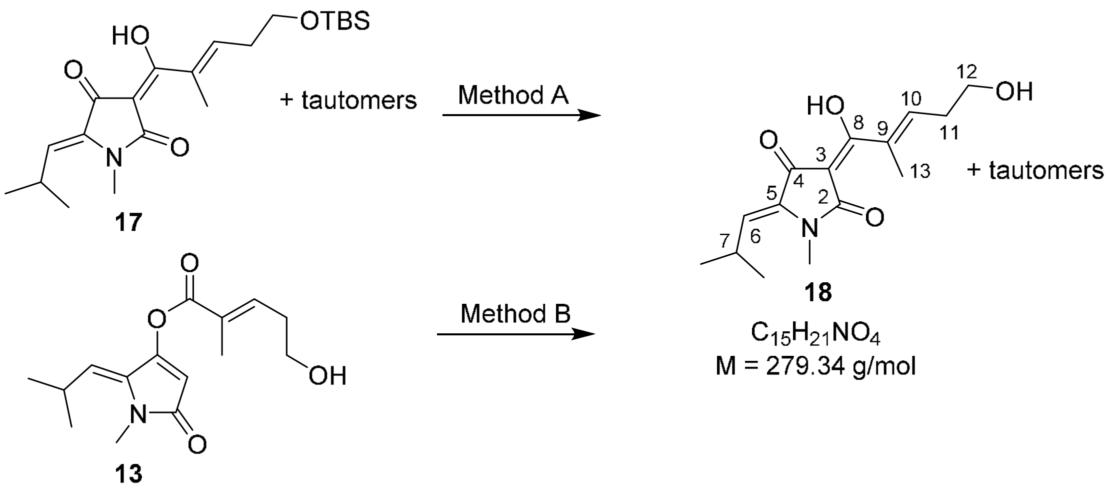

Method A:

Tetramic acid derivative **17** (24.5 mg, 62.3 µmol, 1.00 equiv) was dissolved in MeOH (1 mL) and 1 M aq. HCl (106 µL, 106 µmol, 1.70 equiv) was added dropwise at 0 °C. The reaction mixture was stirred for 20 min at rt. DCM (20 mL) and water (20 mL) were added and the aqueous layer was extracted with DCM (3x60 mL). The combined organic phase was dried over Na_2SO_4 and the solvent was removed under reduced pressure to yield the title compound **18** as an orange oil (17.3 mg, 61.9 µmol, quant.).

Method B:

See general procedure for the *O*-to *C*-rearrangement (Yield: quant.)

R_f 0.40 (10% MeOH/DCM). Due to extended tautomerism only signals of two major tautomers were picked. **^{1}H NMR** (500 MHz, CD_2Cl_2) δ = 6.60/6.54 (2xt, *J* = 7.4 Hz, 1H, H-10), 5.31/5.19 (2xd, *J* = 9.9 Hz, 1H, H-6), 3.79/3.76 (2xt, *J* = 5.6 Hz, 2H, H-12), 3.73 – 3.66/3.64 – 3.59 (2xm, 1H, H-7), 3.05/3.02 (2xs, 3H, N-CH_3), 2.50 (dt, *J* = 6.3 Hz, 2H, H-11), 1.92 (s, 3H, H-13), 1.10/1.04 (2xd, *J* = 6.6 Hz, 6H, 7-$(CH_3)_2$).**^{13}C NMR** (126 MHz, CD_2Cl_2) δ = 189.8/184.1 (C-8), 188.4/181.9 (C-4), 172.6/164.6 (C-2), 142.7/141.2 (C-10), 134.6/132.3 (C-5), 131.1/130.2 (C-9), 126.5/125.9/123.8 (C-6), 103.5/100.8 (C-3), 61.7/61.6 (C-12), 33.0/32.9 (C-11), 26.3/25.3 (C-7), 25.7/25.3 (N-CH_3), 23.7/23.5 (7-$(CH_3)_2$), 12.8/12.7 (C-13). **HRMS (ESI)** *m/z*: calcd for $C_{15}H_{21}NO_4H$ $[M + H]^+$: 280.1543, found: 280.1544.

Synthesis of Compound 19

Ethyl (*Z*)-3-allyl-1-methyl-5-(2-methylpropylidene)-2,4-dioxopyrrolidine-3-carboxylate

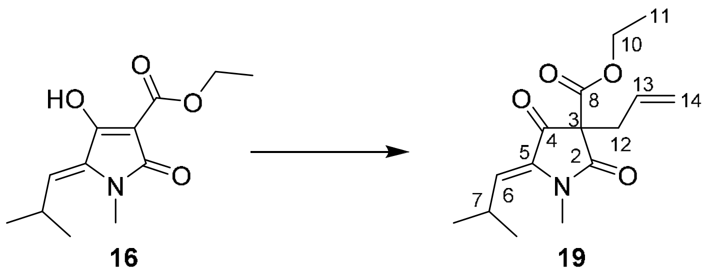

$C_{15}H_{21}NO_4$
M = 279.34 g/mol

DMF (8 mL) was added to tetramic acid derivative **16** (105 mg, 439 µmol, 1.00 equiv) and K_2CO_3 (91.0 mg, 658 µmol, 1.50 equiv). Allyl bromide (49.3 µL, 571 µmol, 1.30 equiv) was added and the reaction mixture was stirred 5 h at 45 °C. DCM (20 mL), aq. sat. $NaHCO_3$ solution (10 mL) and water (20 mL) were added, the phases separated and the aqueous layer was extracted with DCM (3x20 mL). The combined organic phase was dried over Na_2SO_4 and the solvent was removed under reduced pressure. The crude product was purified by flash chromatography (30% EtOAc/CyH) to yield the racemic title compound **19** as a white solid (100 mg, 358 µmol, 82%).

R_f 0.28 (30% EtOAc/CyH). **^{1}H NMR** (700 MHz, CD_2Cl_2) δ = 5.54 – 5.48 (m, 1H, H-13), 5.14 – 5.10 (m, 2H, H-14), 5.04 (d, *J* = 10.1 Hz, 1H, H-6), 4.19 – 4.10 (m, 2H, H-10), 3.63 – 3.58 (m, 1H, H-7), 3.06 (s, 3H, N-CH_3), 2.83 – 2.81 (m, 2H, H-12), 1.18 (t, *J* = 7.1 Hz, 3H, H-11), 1.06 (d, *J* = 6.7 Hz, 3H, 7-$(CH_3)_2$), 1.04 (d, *J* = 6.7 Hz, 3H, 7-$(CH_3)_2$). **^{13}C NMR** (176 MHz, CD_2Cl_2) δ = 193.7 (C-4), 167.7 (C-2), 165.6 (C-8), 134.3 (C-5), 130.8 (C-13), 125.6 (C-6), 120.8 (C-14), 63.0 (C-10), 62.9 (C-3), 35.8 (C-12), 26.7 (N-CH_3), 26.0 (C-7), 23.5 (7-$(CH_3)_2$), 14.2 (C-11). **HRMS (ESI)** *m/z*: calcd for $C_{15}H_{21}NO_4H$ $[M + H]^+$: 280.1543, found: 280.1541. Analytical, chiral HPLC showed the existence of the two enantiomers (98% *n*-hexane/ethanol, retention time 2.57 min and 2.76 min, using a DAICEL Chiralpak IG-U <2 µm; 3.0 x 100 mm)

[1] G. R. Fulmer, A. J. M. Miller, N. H. Sherden, H. E. Gottlieb, A. Nudelman, B. M. Stoltz, J. E. Bercaw, K. I. Goldberg, *Organometallics* **2010**, *29*, 2176–2179.

[2] S. Tan, K. C. Ludwig, A. Müller, T. Schneider, J. R. Nodwell, *ACS Chem. Biol.* **2019**, *14*, 966–974.

[3] J. Radeck, S. Gebhard, P. S. Orchard, M. Kirchner, S. Bauer, T. Mascher, G. Fritz, *Mol. Microbiol.* **2016**, *100*, 607–620.

[4] M. Bánziger, J. F. Mcgarrity, T. Meul, *J. Org. Chem.* **1993**, *58*, 4010–4012.

[5] R. A. Fernandes, V. P. Chavan, *Tetrahedron Asymmetry* **2011**, *22*, 1312–1319.

[6] J. L. Freeman, M. A. Brimble, D. P. Furkert, *Org. Chem. Front.* **2019**, *6*, 2954–2963.

[7] L. Lin, C. Romano, C. Mazet, *J. Am. Chem. Soc.* **2016**, *138*, 10344–10350.

[8] A. Hager, C. Kuttruff, D. Hager, D. Terwilliger, D. Trauner, *Synlett* **2013**, *24*, 1915–1920.

3. Copies of NMR Spectra

NMR-Spectra for Compound 3

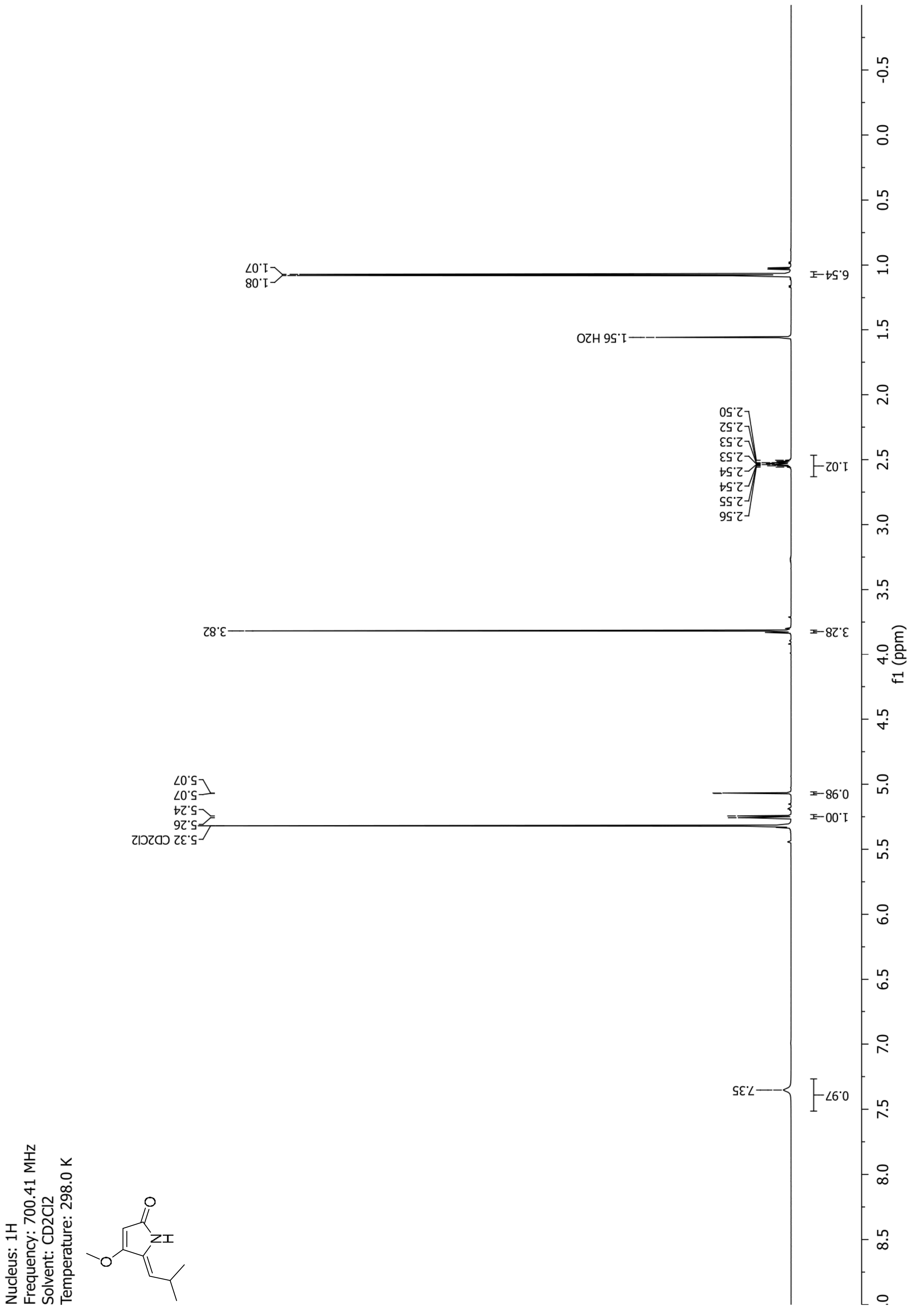

Nucleus: 13C
Frequency: 176.12 MHz
Solvent: CD2Cl2
Temperature: 298.0 K

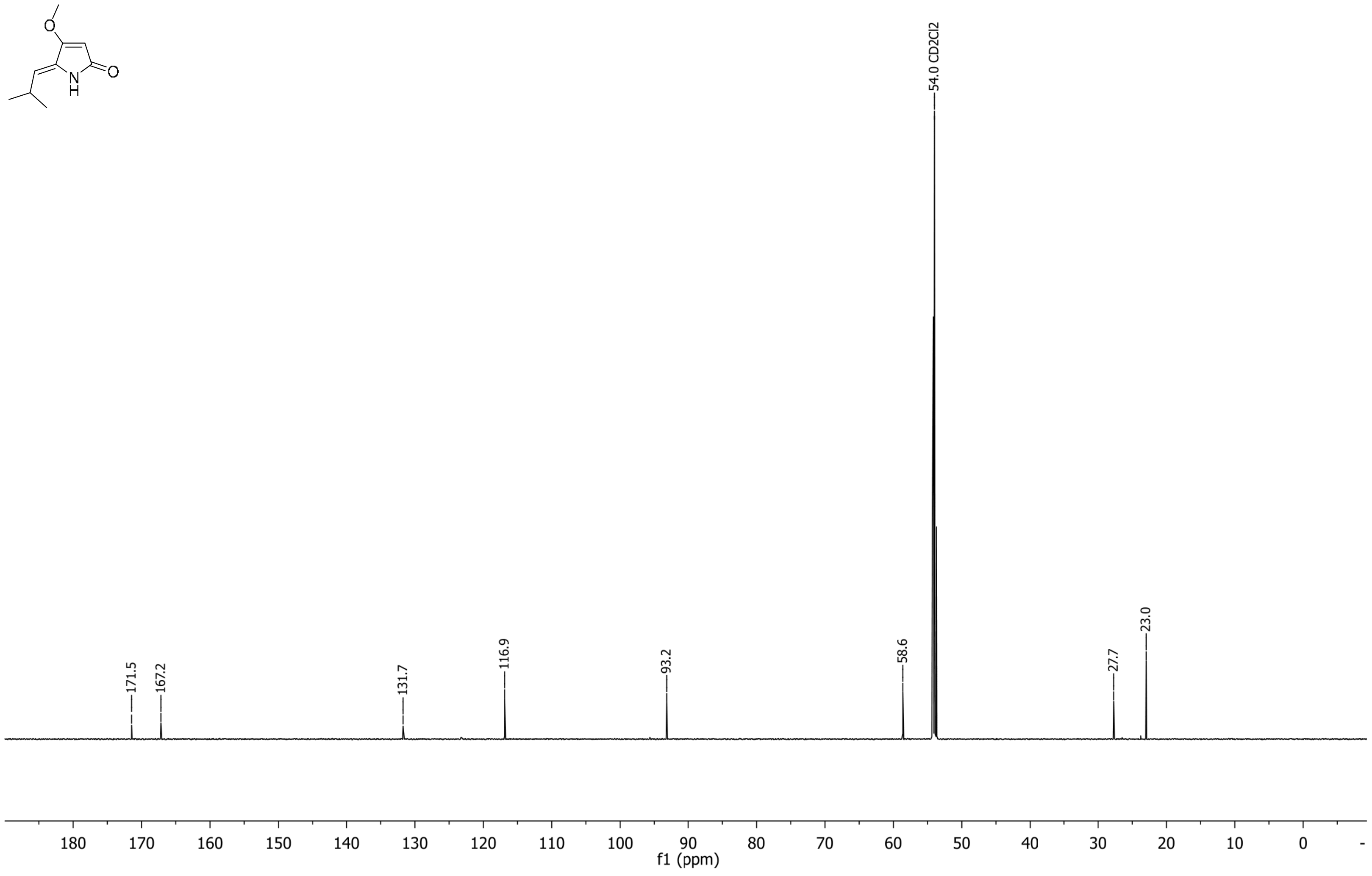

NMR-Spectra for Compound 4

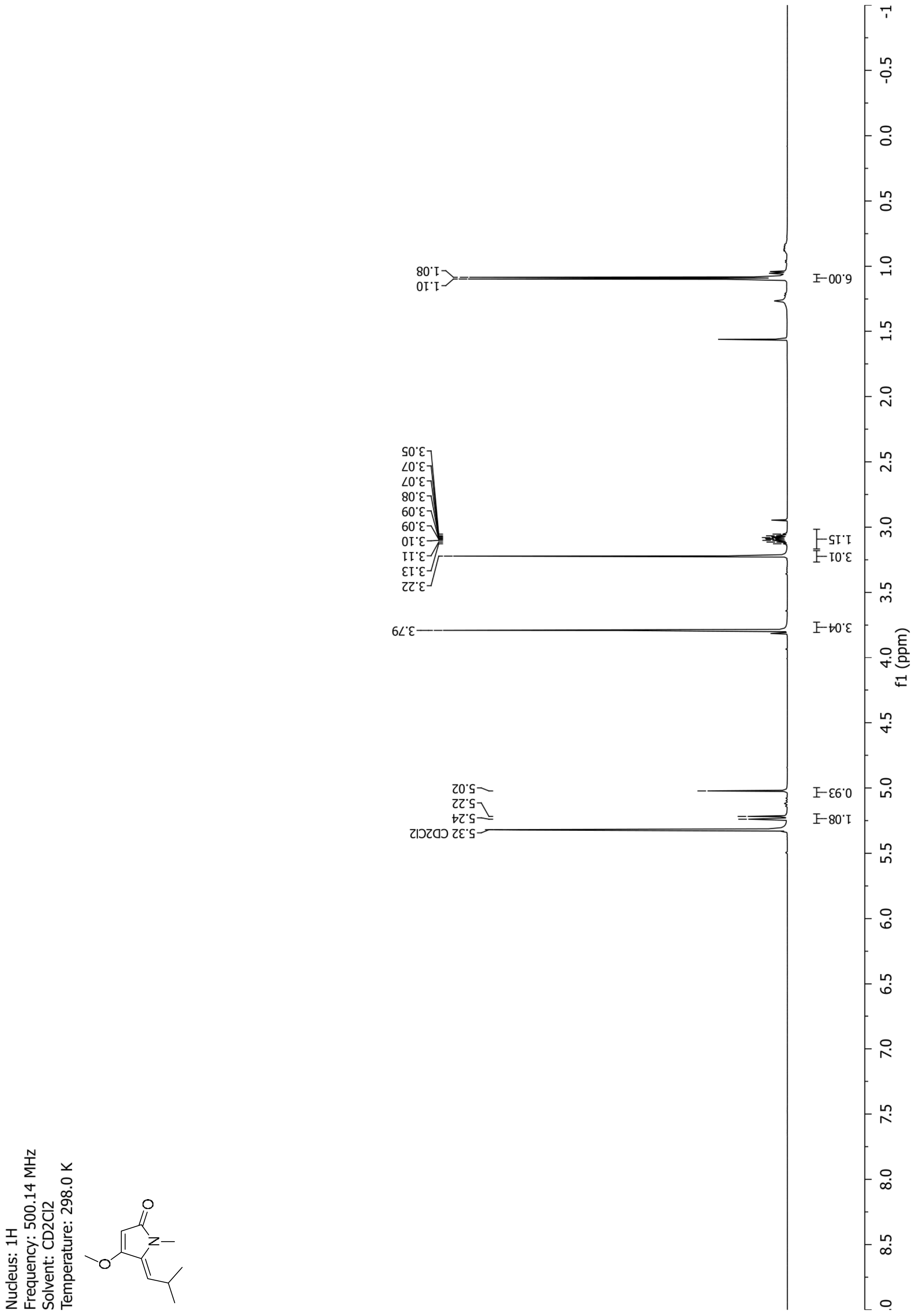

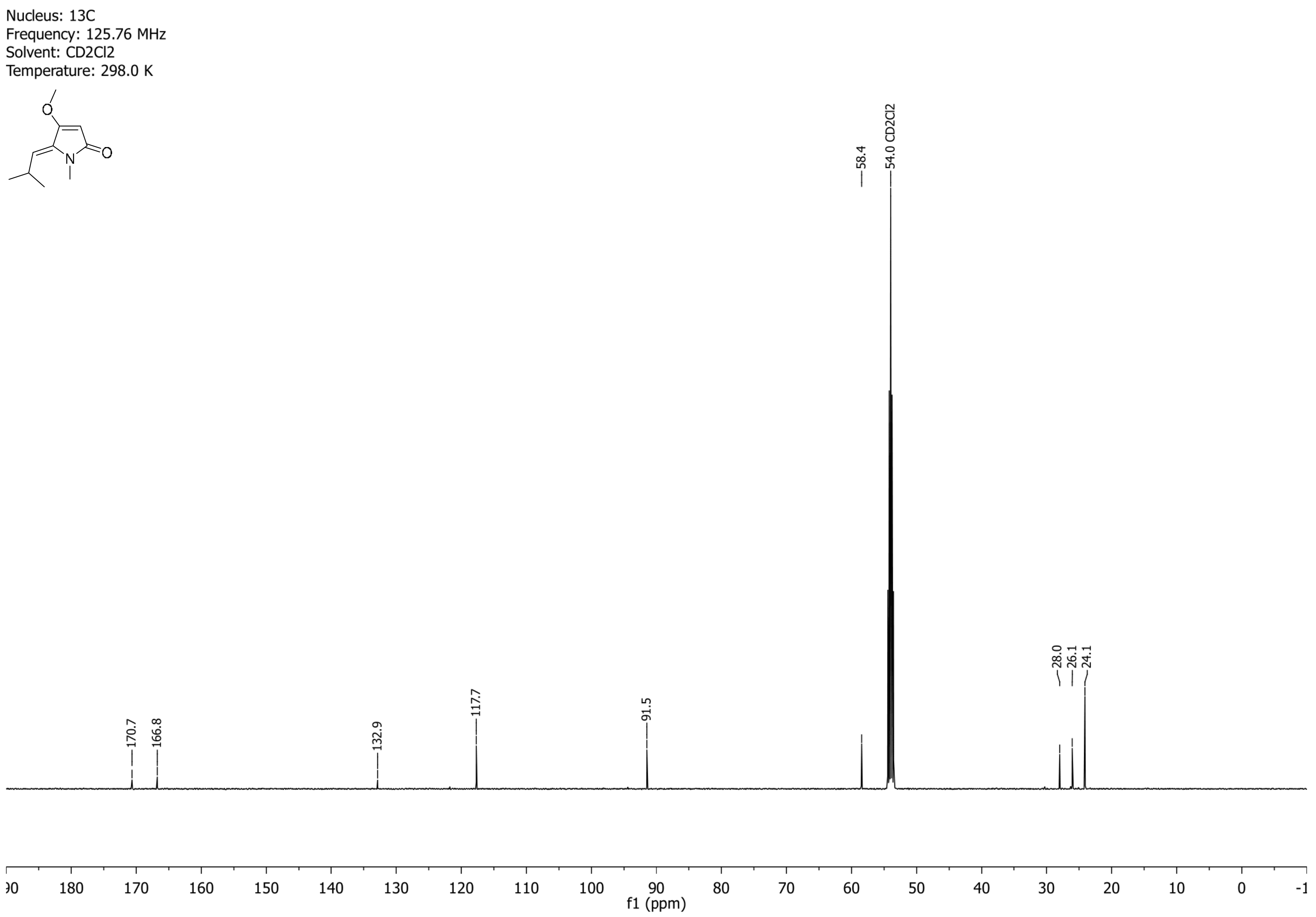
Nucleus: 13C
Frequency: 125.76 MHz
Solvent: CD2Cl2
Temperature: 298.0 K
O
N
O
170.7
166.8
132.9
117.7
91.5
58.4
54.0 CD2Cl2
28.0
26.1
24.1
f1 (ppm)

NMR-Spectra for Compound 5

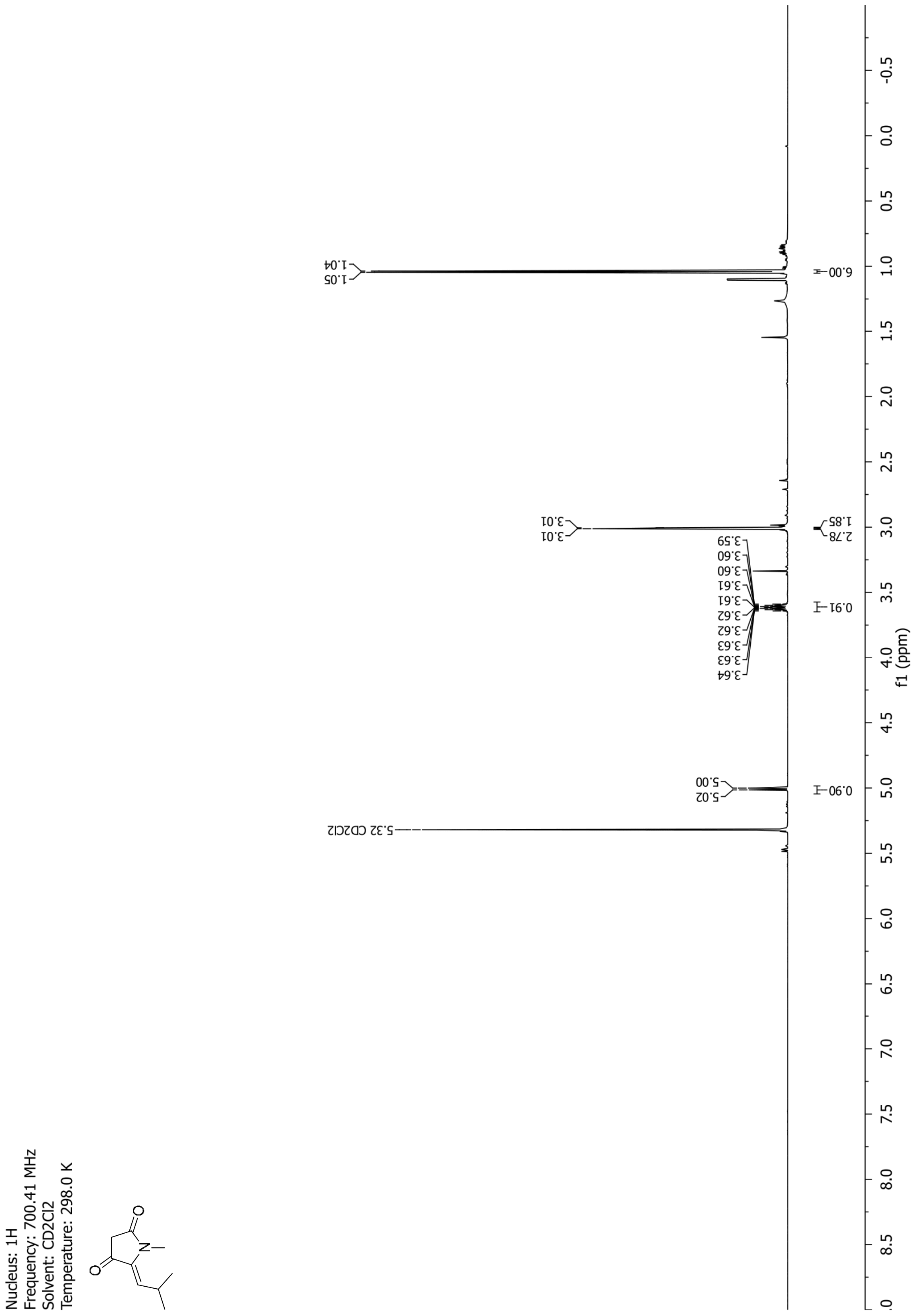

Nucleus: 13C
Frequency: 176.12 MHz
Solvent: CD2Cl2
Temperature: 298.0 K

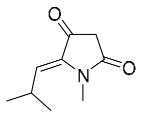

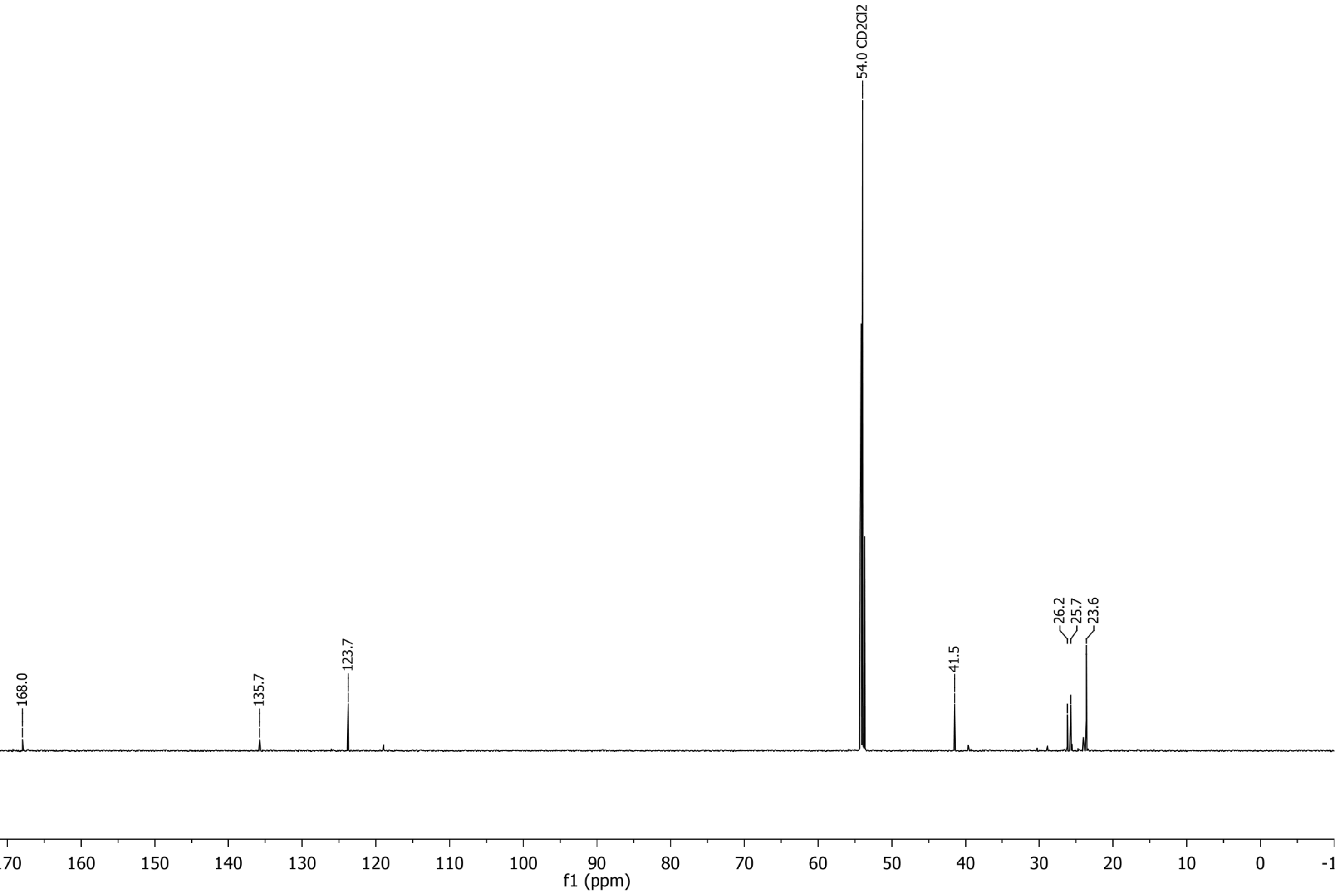

NMR-Spectra for Compound 9

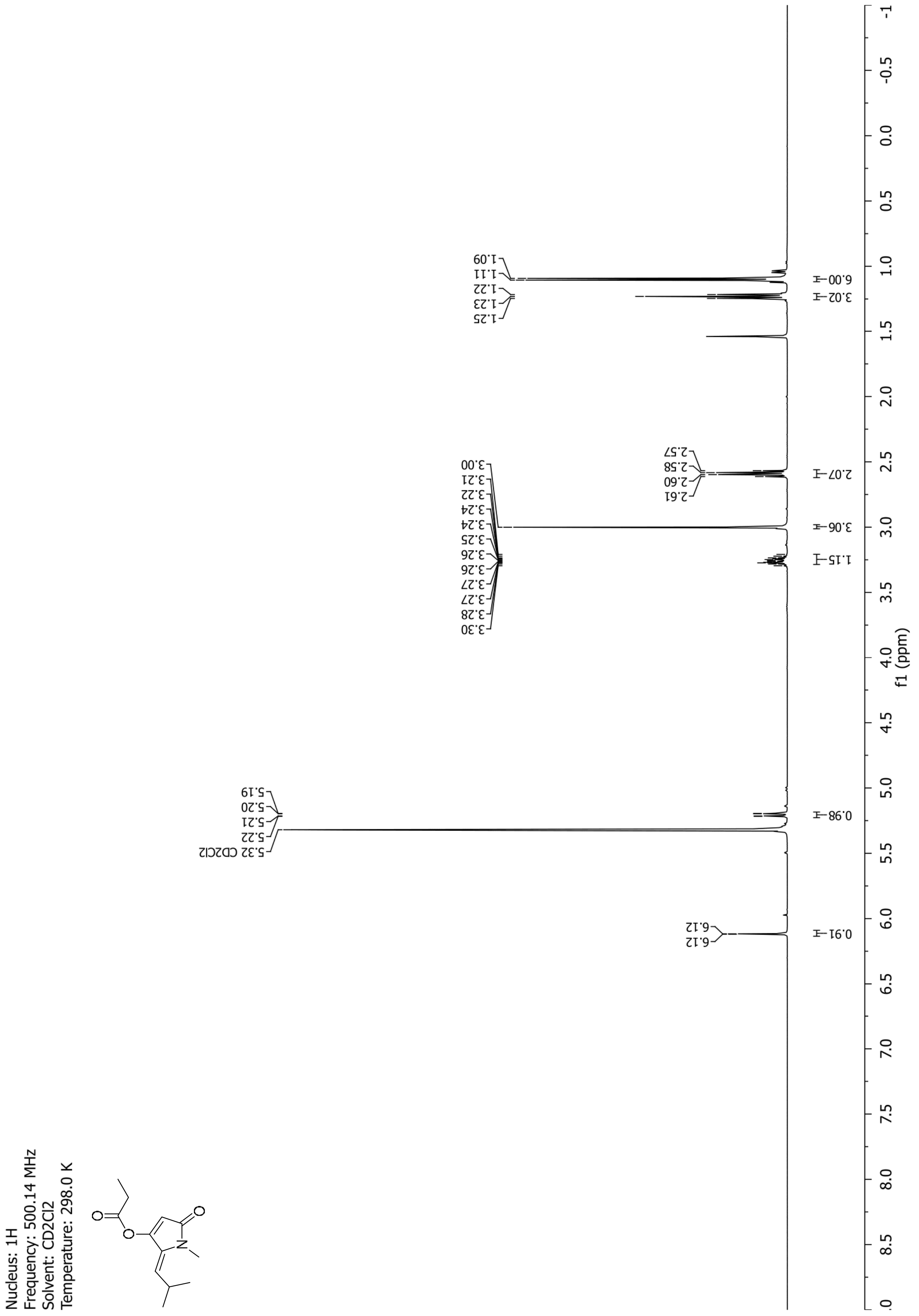

Nucleus: 13C
Frequency: 125.76 MHz
Solvent: CD2Cl2
Temperature: 298.0 K

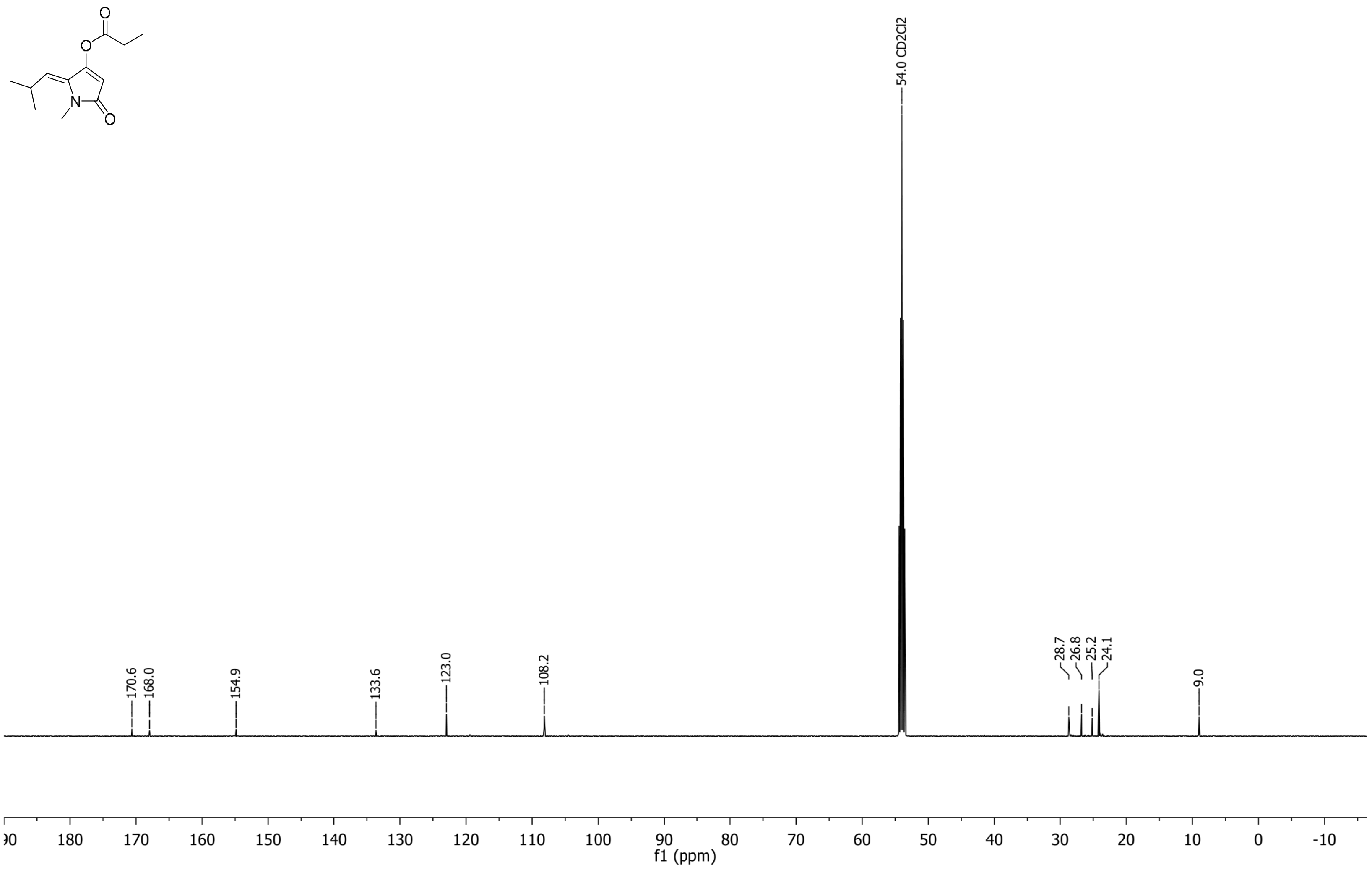

NMR-Spectra for Compound 10

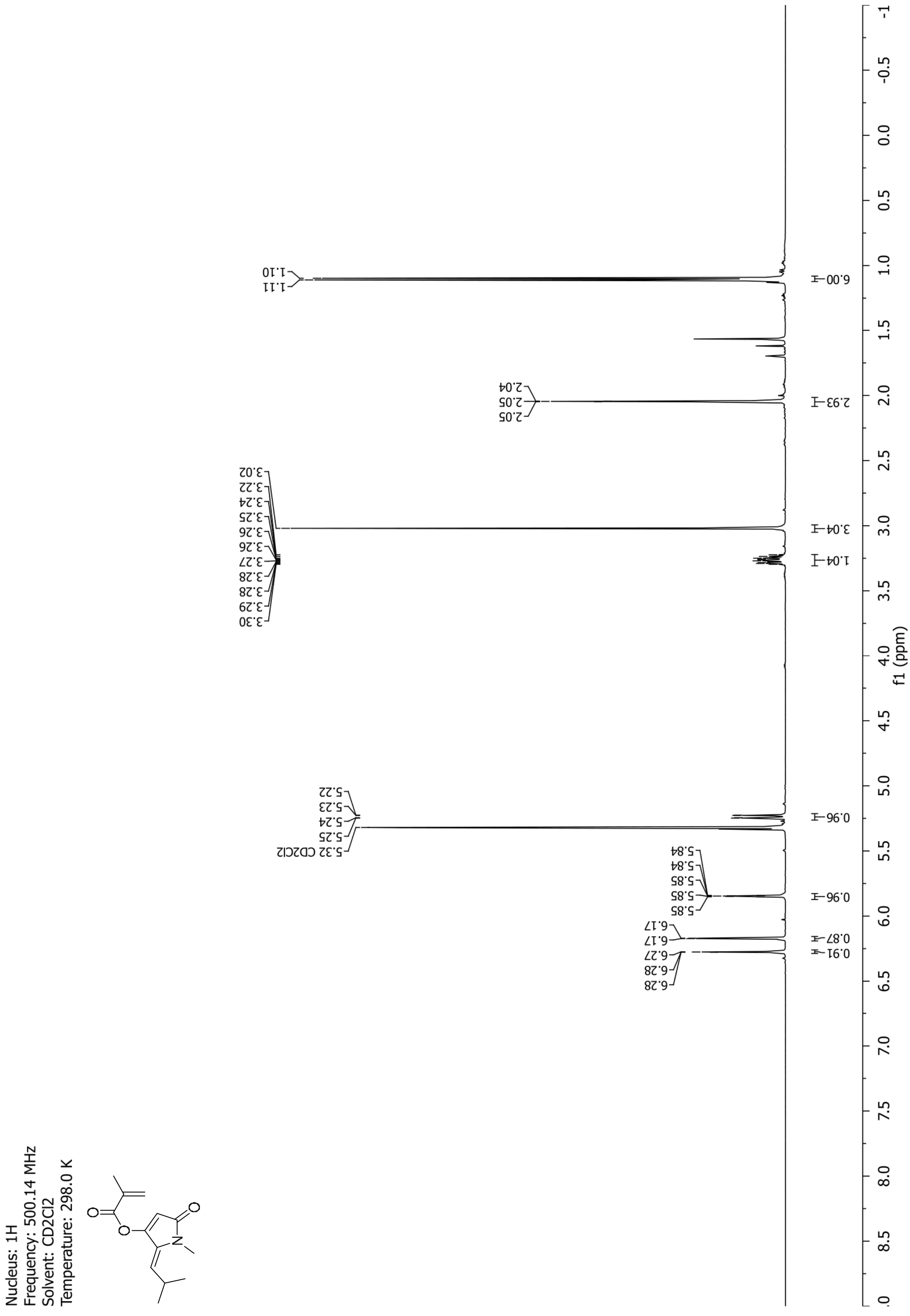

Nucleus: 13C
Frequency: 125.76 MHz
Solvent: CD2Cl2
Temperature: 298.0 K

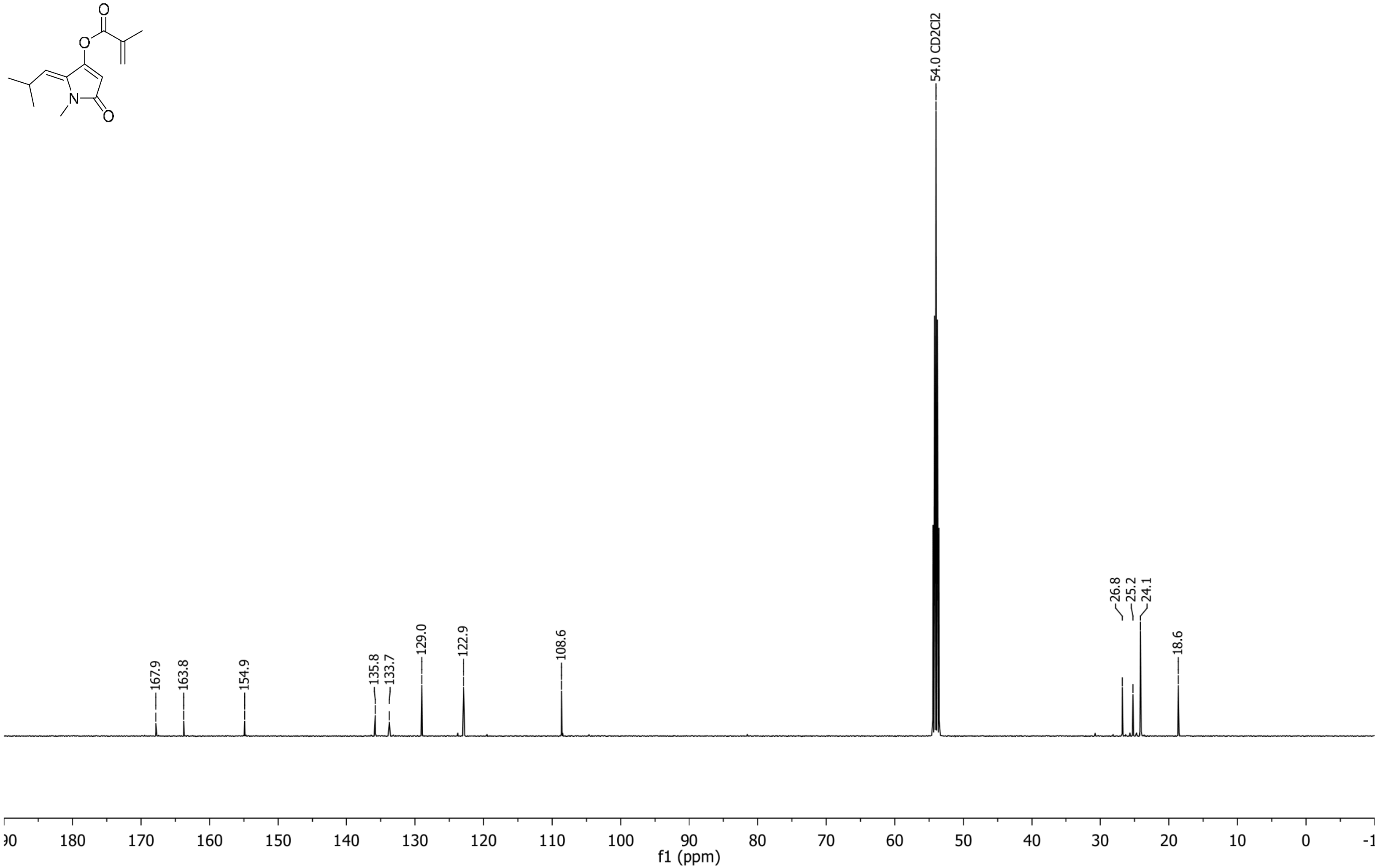

NMR-Spectra for Compound 11

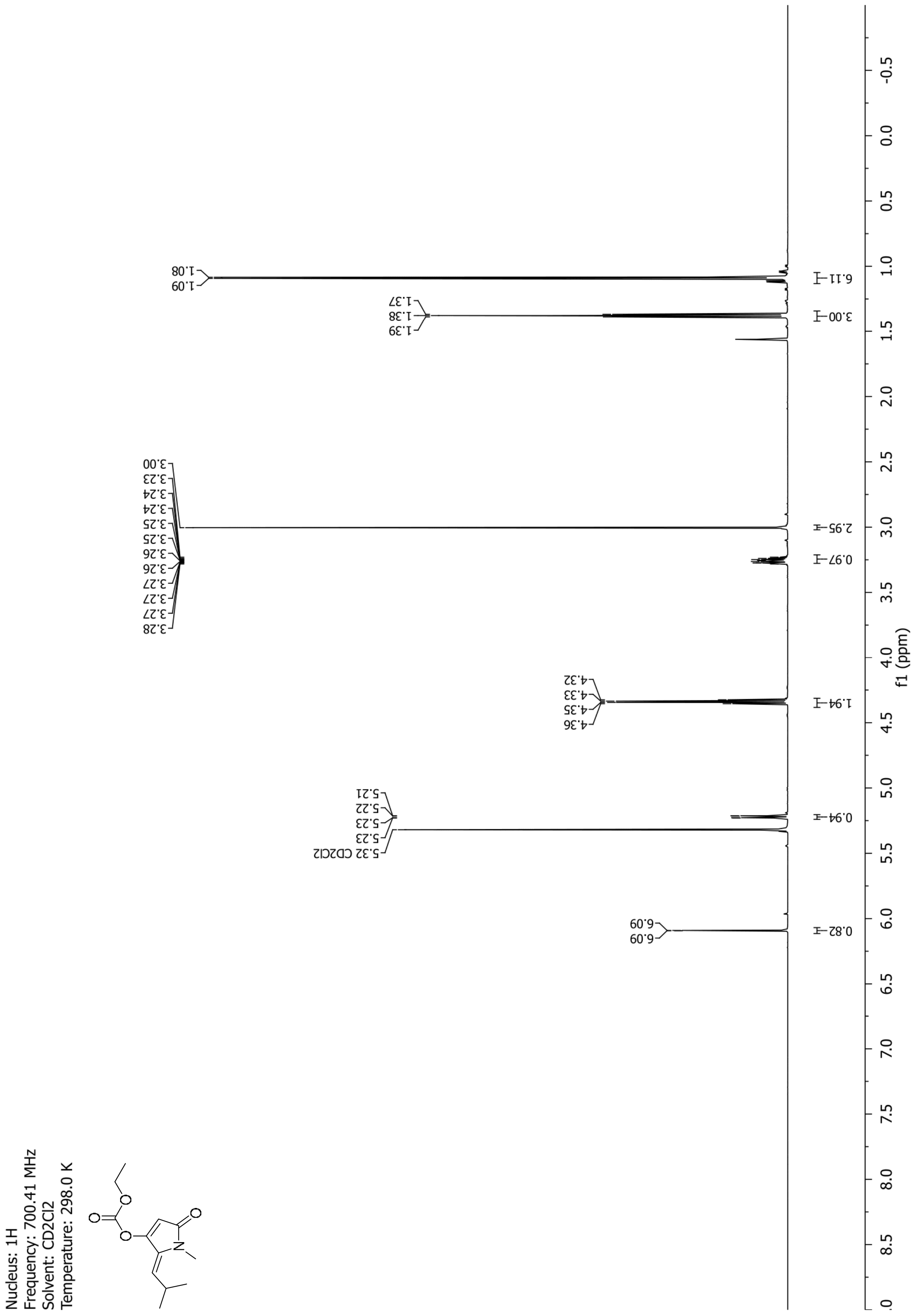

Nucleus: 13C
Frequency: 176.12 MHz
Solvent: CD2Cl2
Temperature: 298.0 K

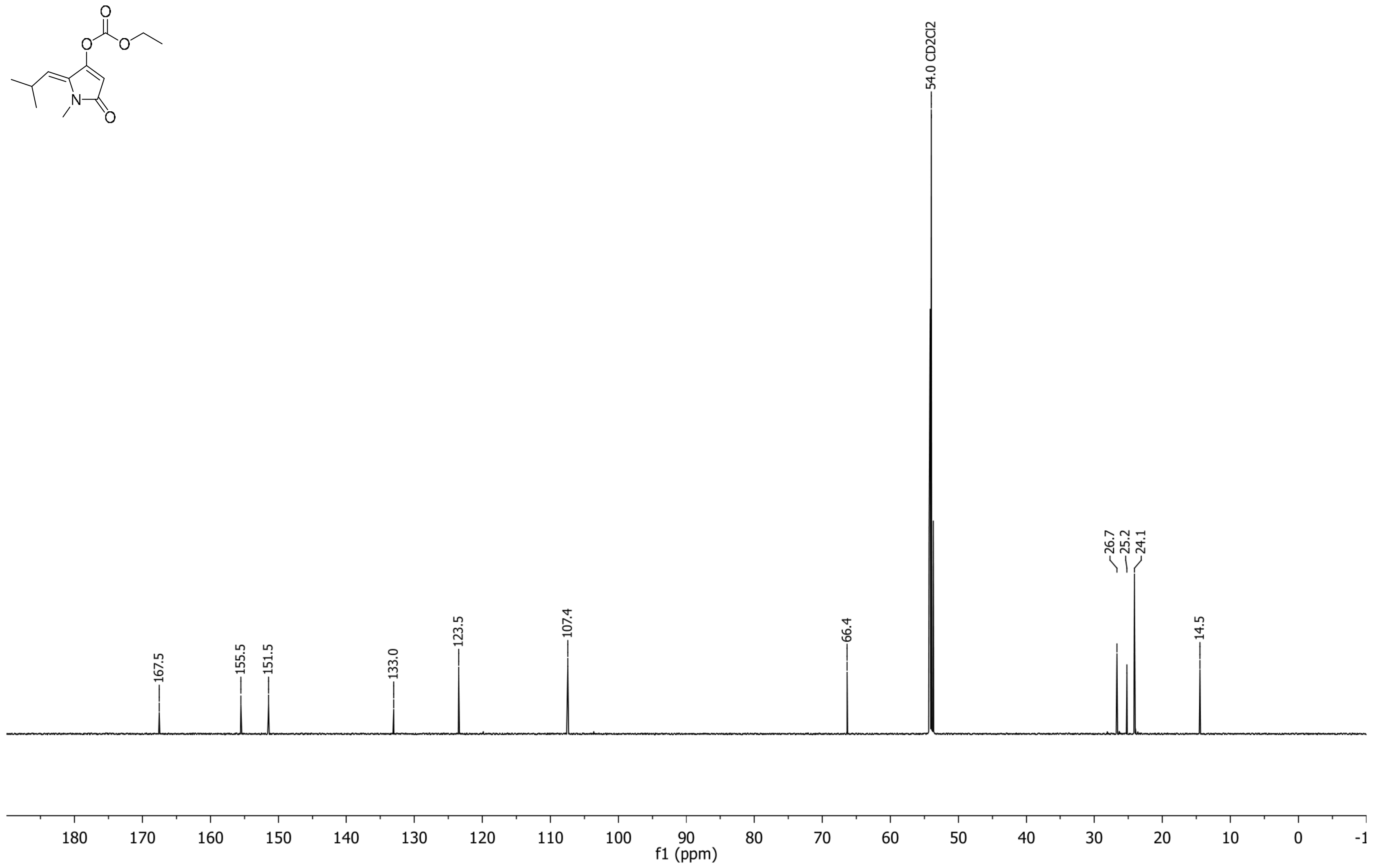

NMR-Spectra for Compound 7.I

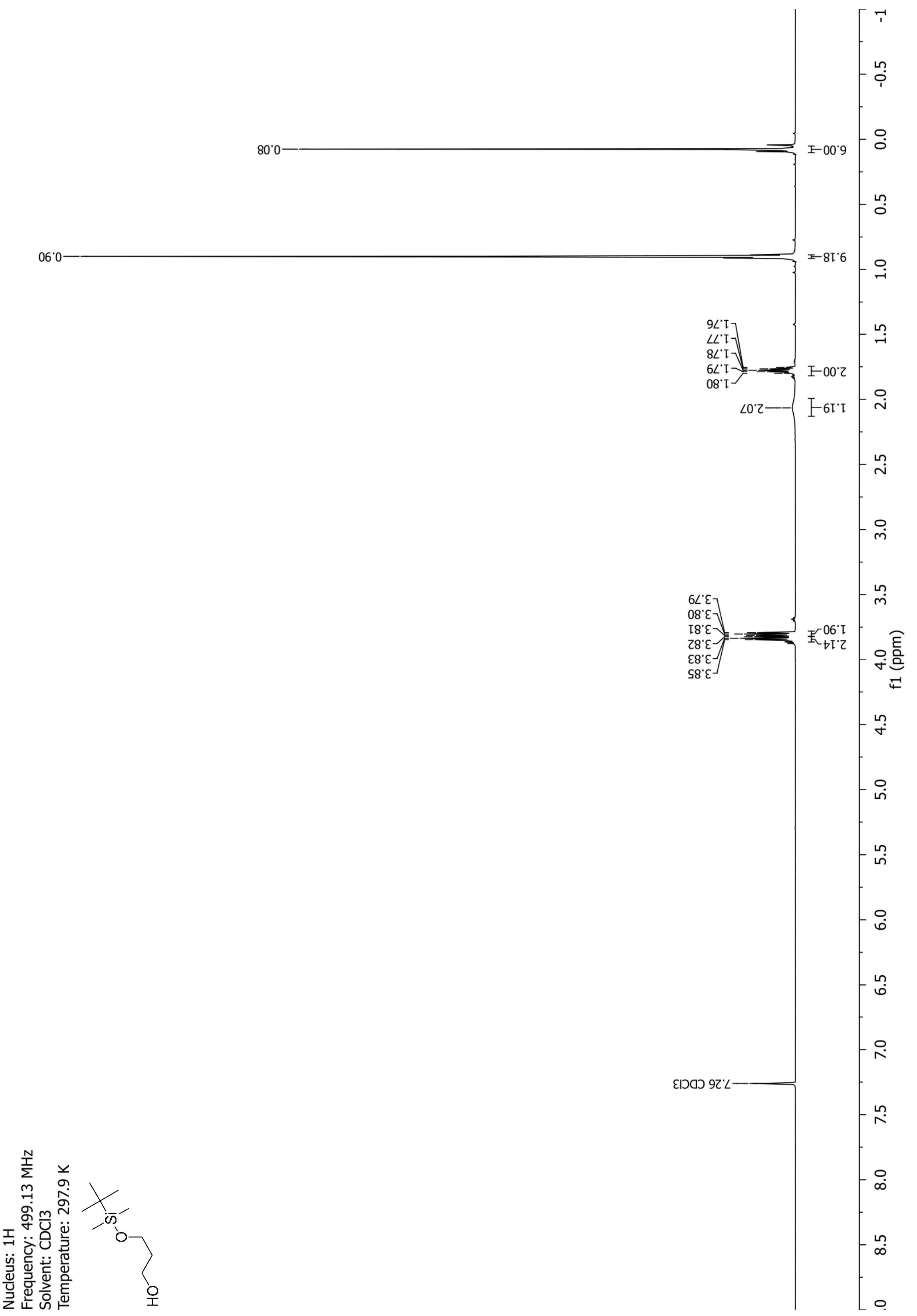

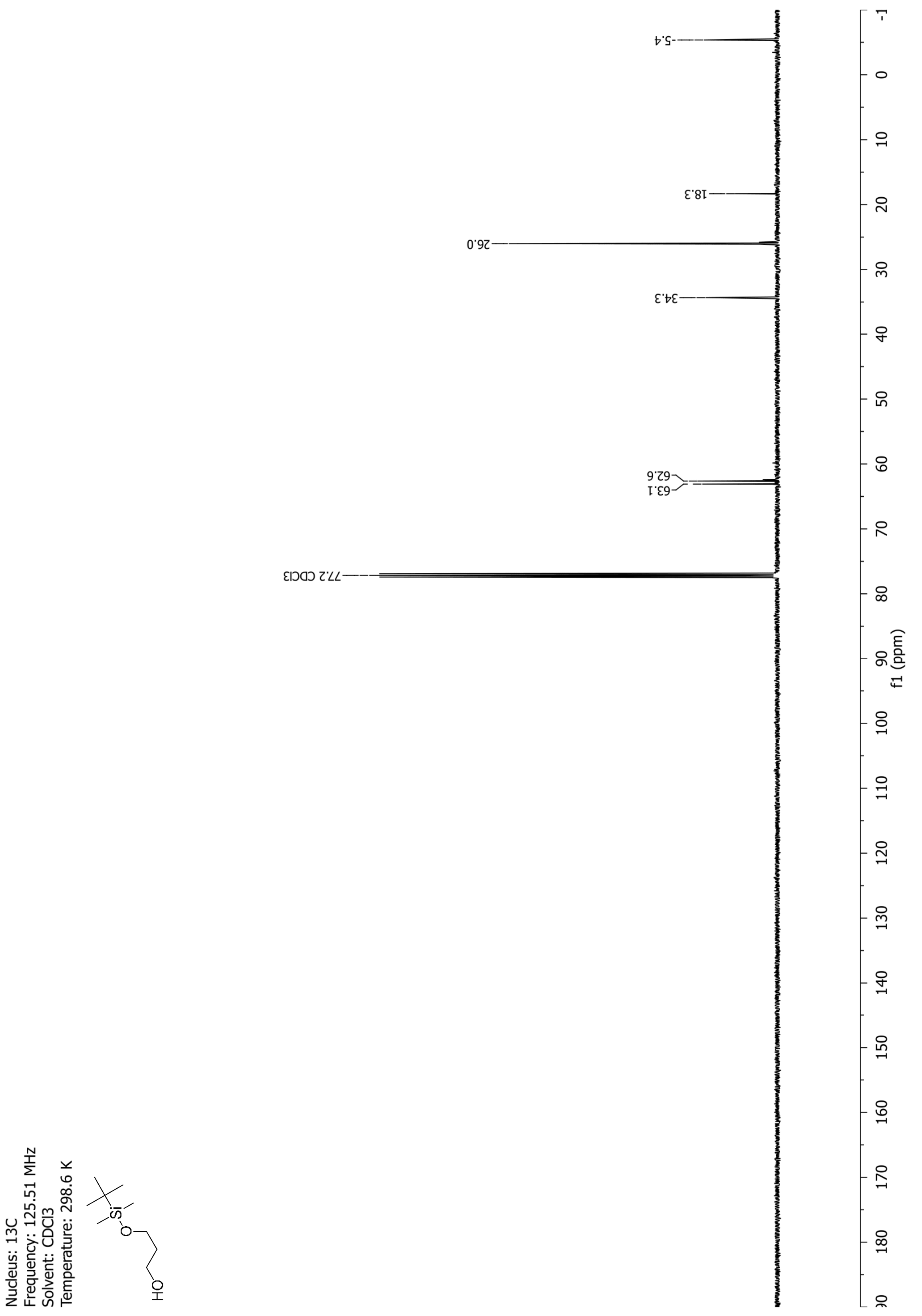
Nucleus: 13C
Frequency: 125.51 MHz
Solvent: CDCl3
Temperature: 298.6 K
HO
O
Si
-5.4
18.3
26.0
34.3
62.6
63.1
77.2 CDCl3
f1 (ppm)

NMR-Spectra for Compound 7

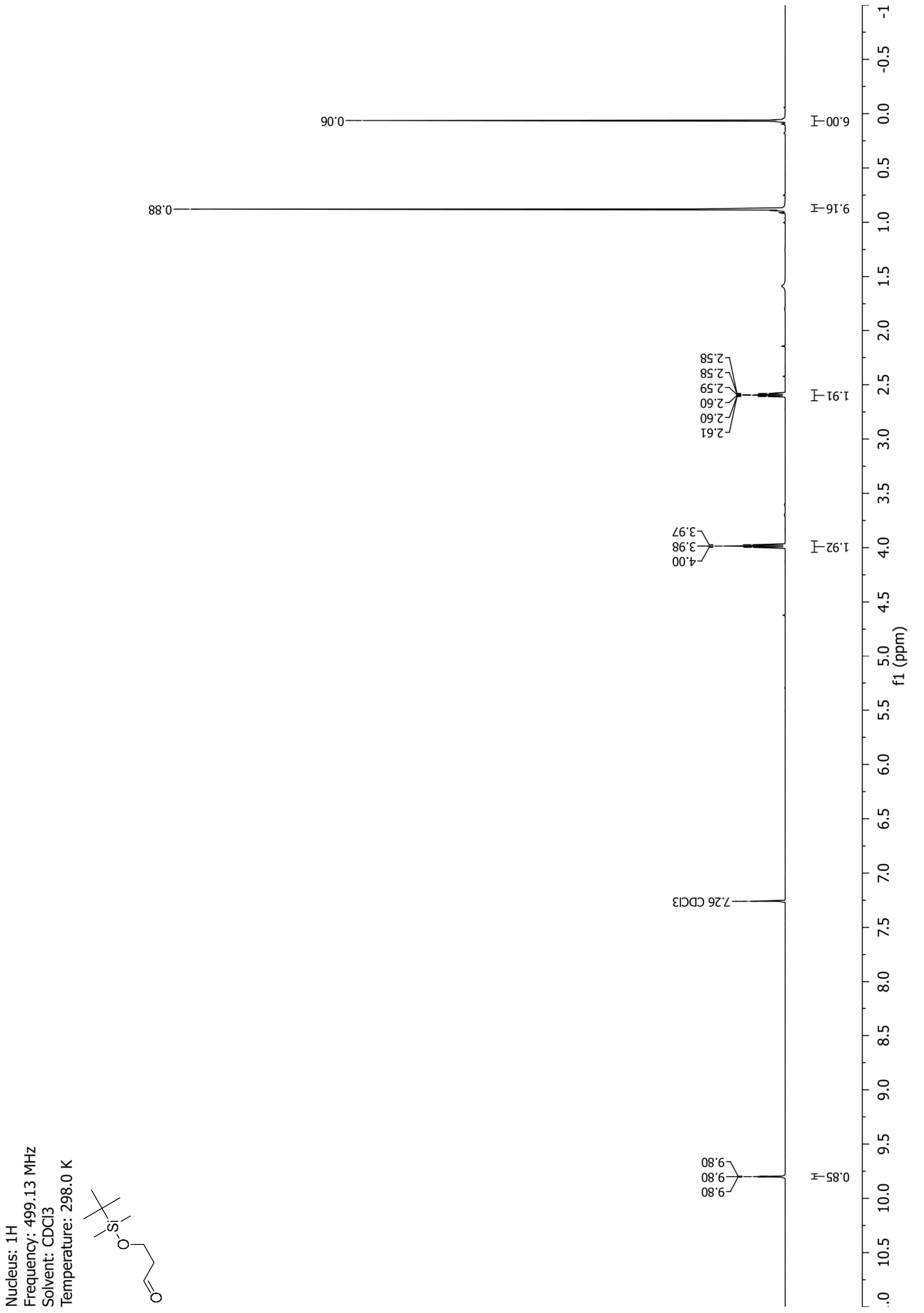

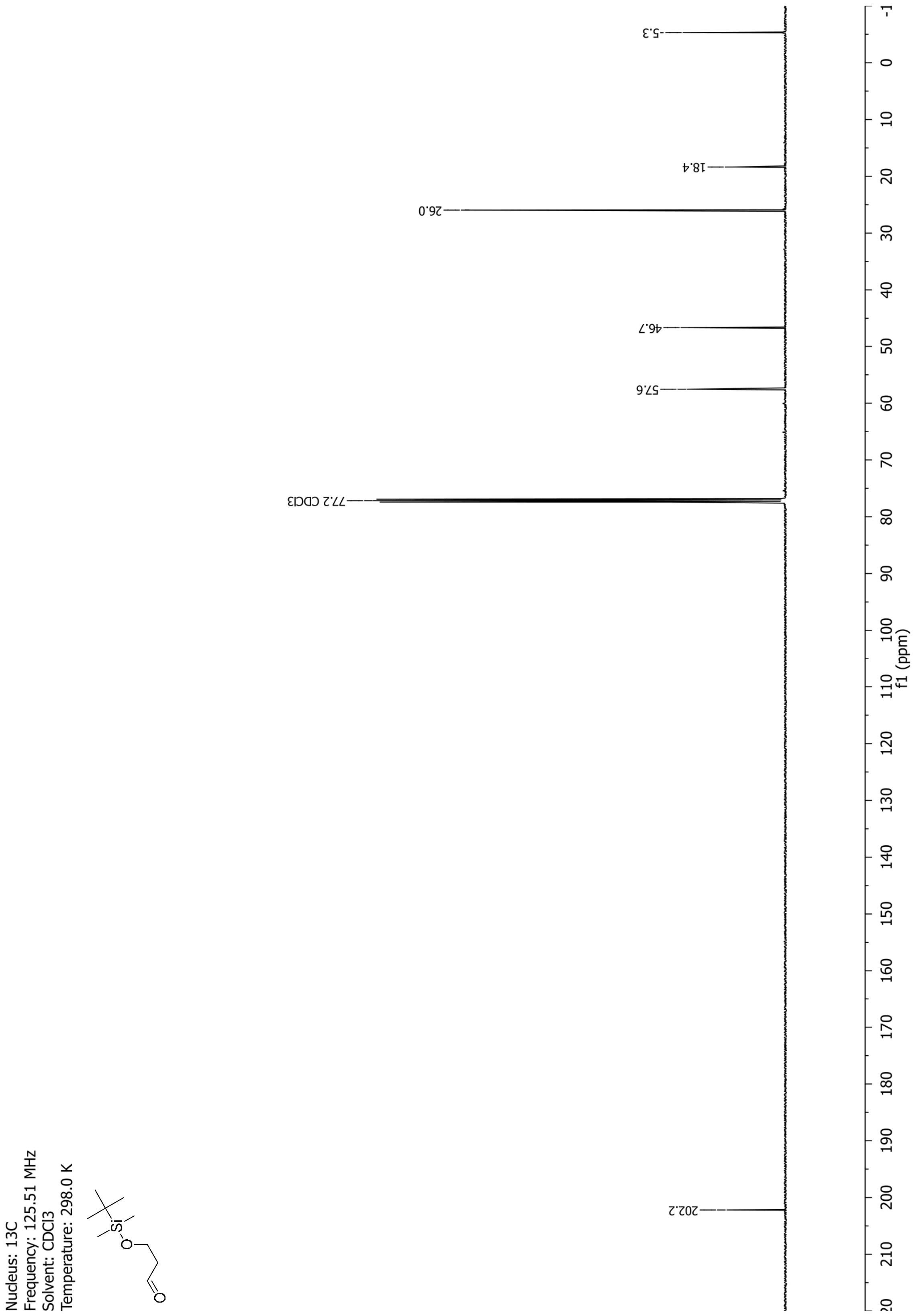

Nucleus: 13C
Frequency: 125.51 MHz
Solvent: CDCl3
Temperature: 298.0 K
Si
O
O
202.2
77.2 CDCl3
57.6
46.7
26.0
18.4
-5.3
f1 (ppm)

NMR-Spectra for Compound 8.I*E*

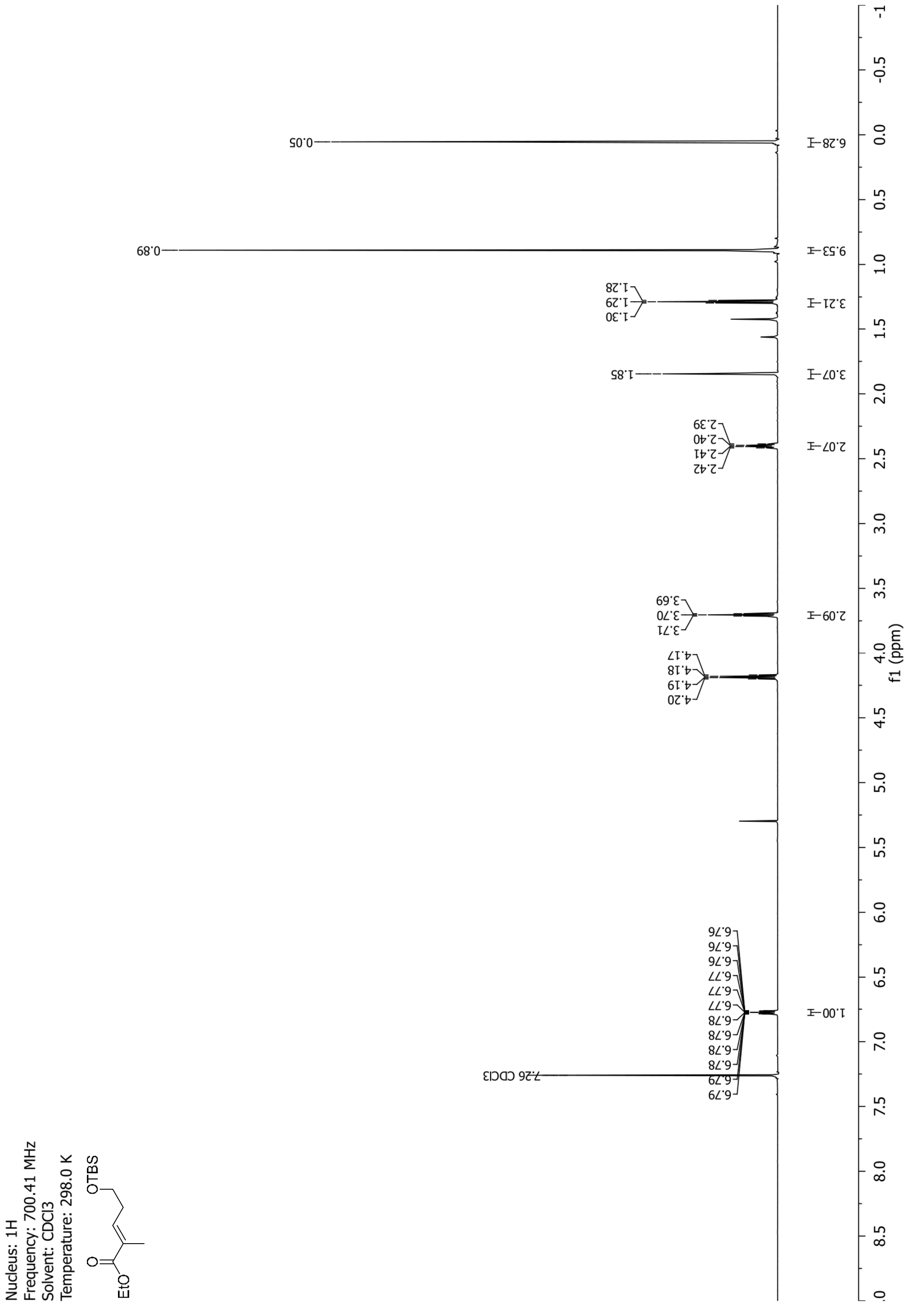

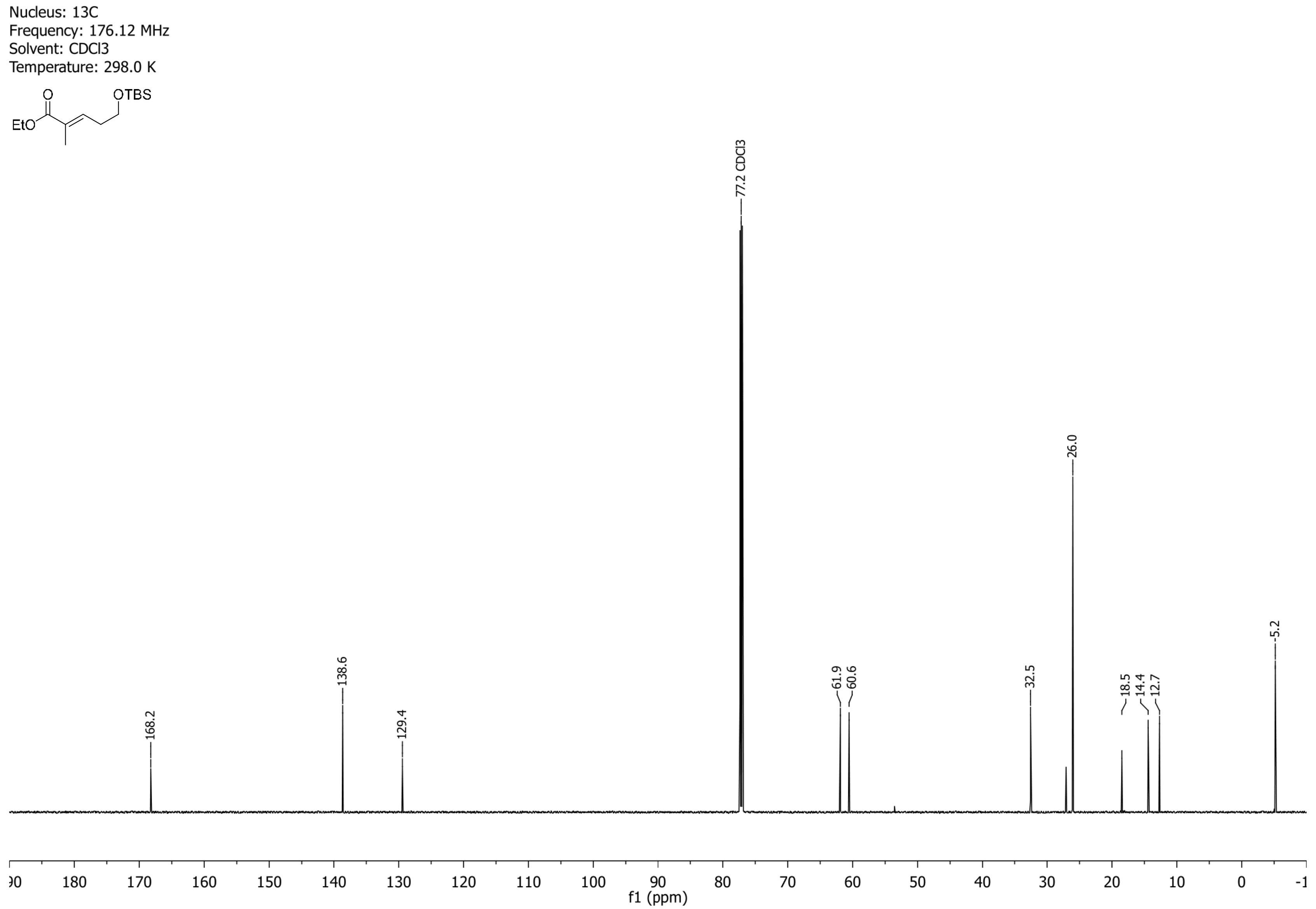
Nucleus: 13C
Frequency: 176.12 MHz
Solvent: CDCl3
Temperature: 298.0 K
O
OTBS
EtO
77.2 CDCl3
26.0
-5.2
168.2
138.6
129.4
61.9
60.6
32.5
18.5
14.4
12.7
f1 (ppm)

NMR-Spectra for Compound 8.IZ

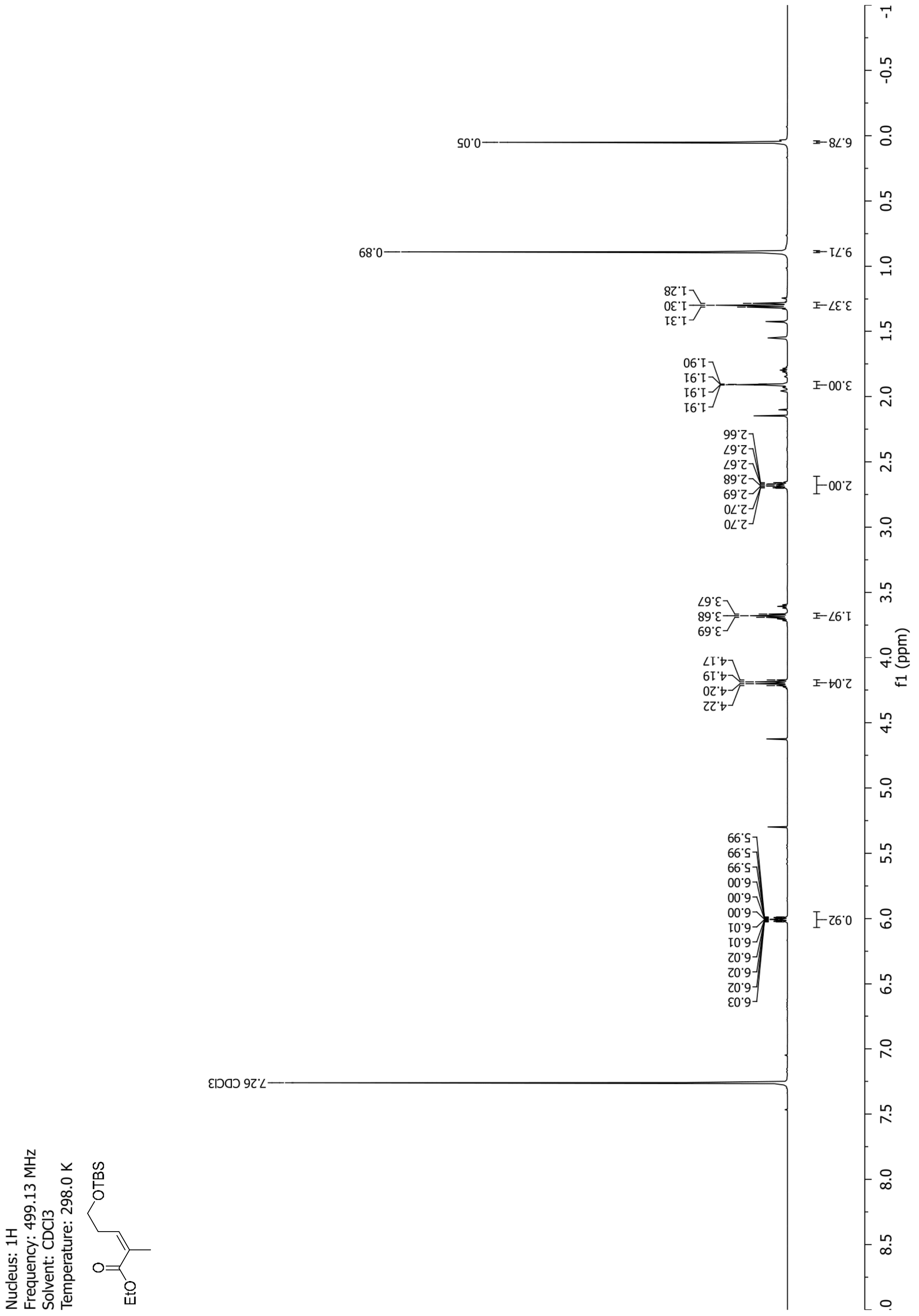

Nucleus: 13C
Frequency: 125.51 MHz
Solvent: CDCl3
Temperature: 298.0 K

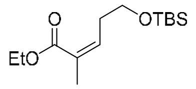

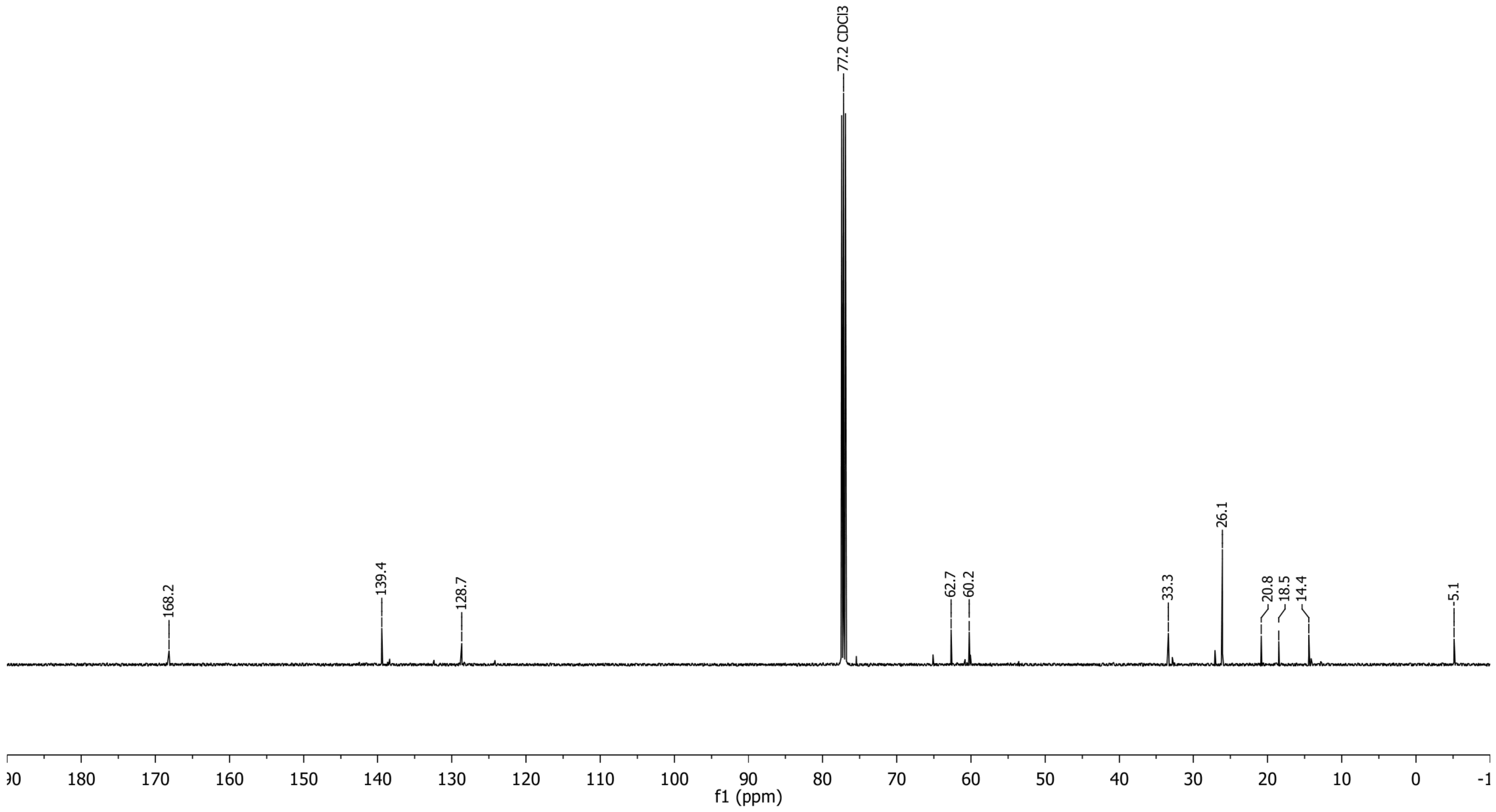

NMR-Spectra for Compound 8

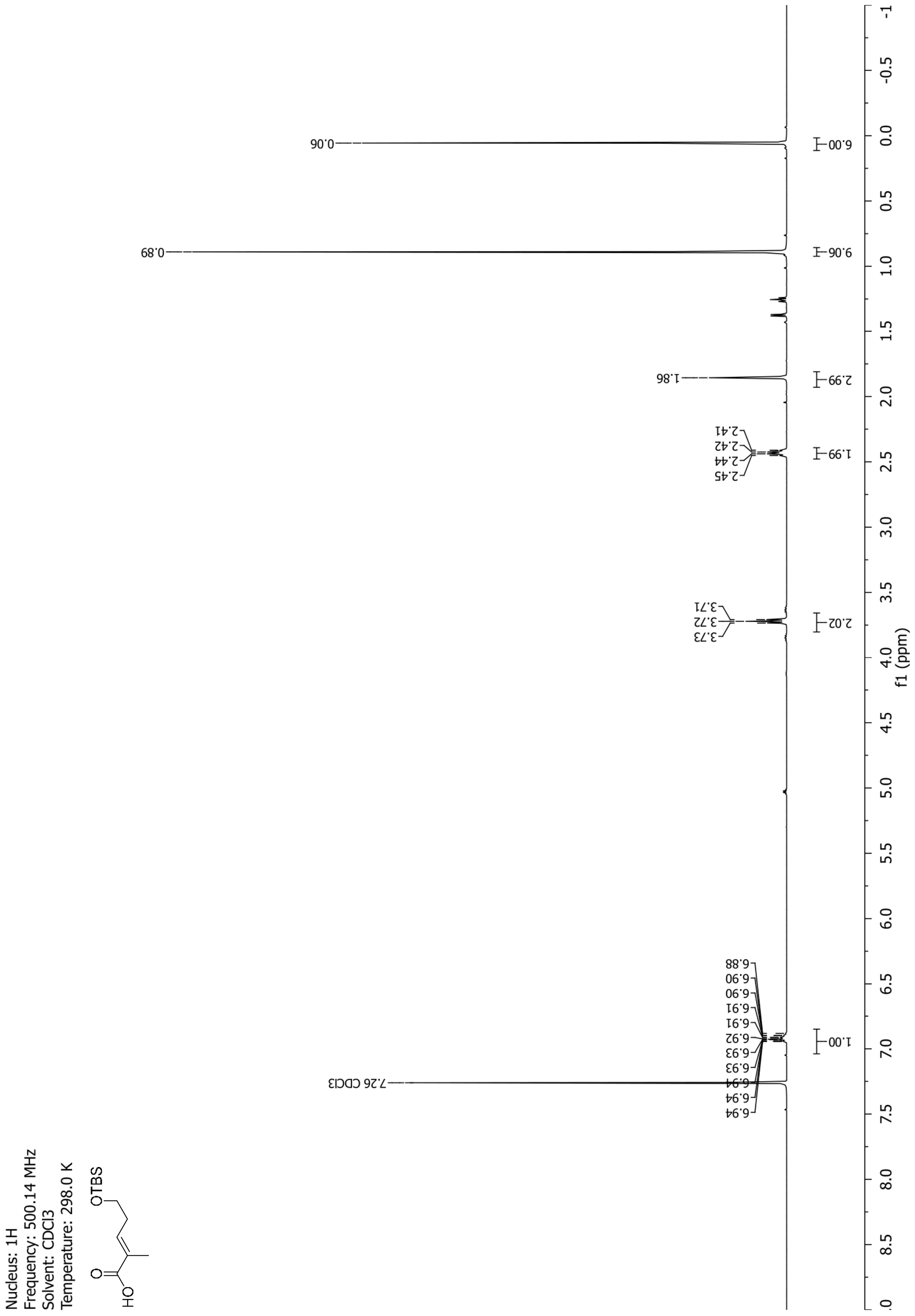

Nucleus: 13C
Frequency: 125.76 MHz
Solvent: CDCl3
Temperature: 298.0 K

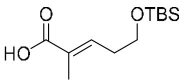

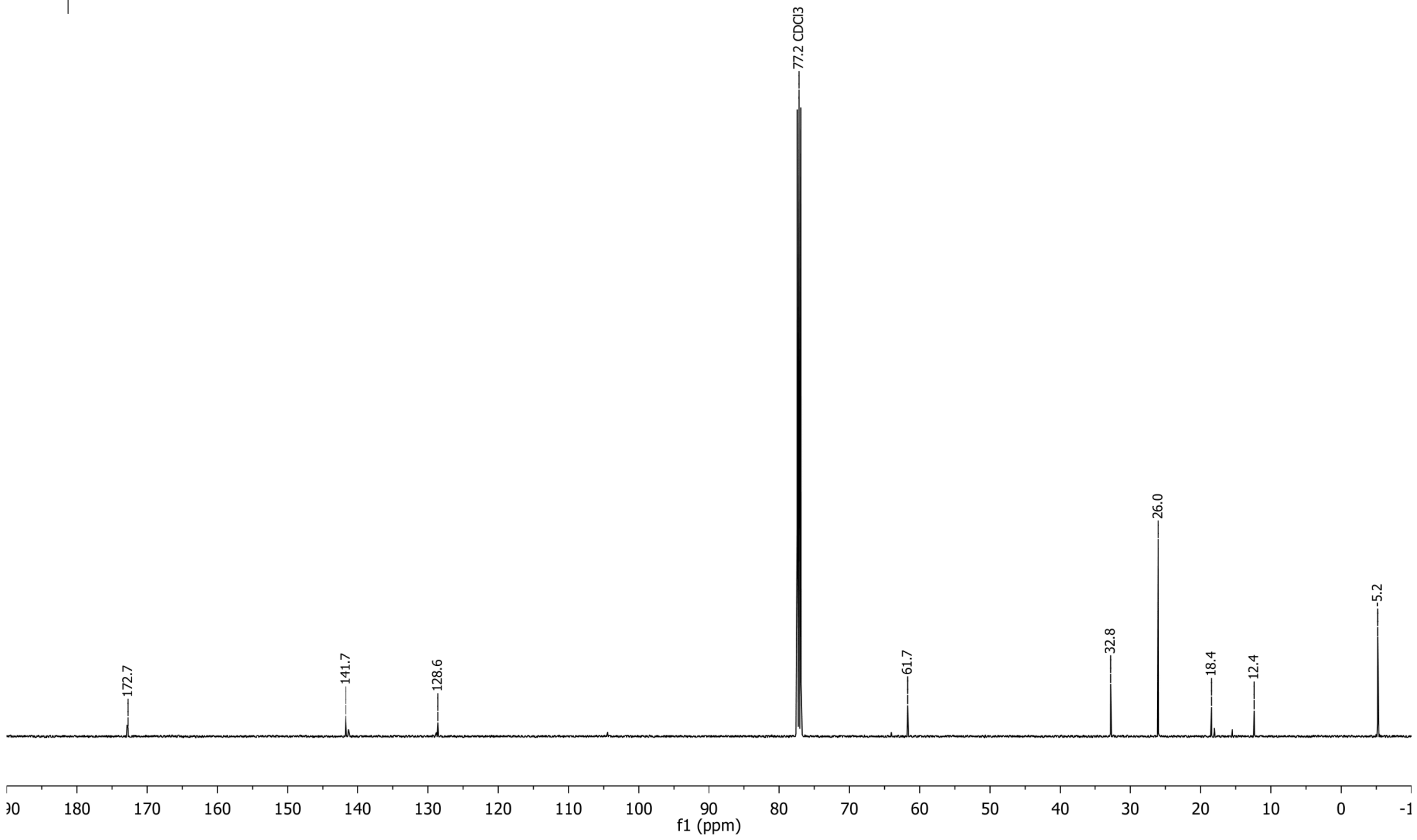

NMR-Spectra for Compound 12

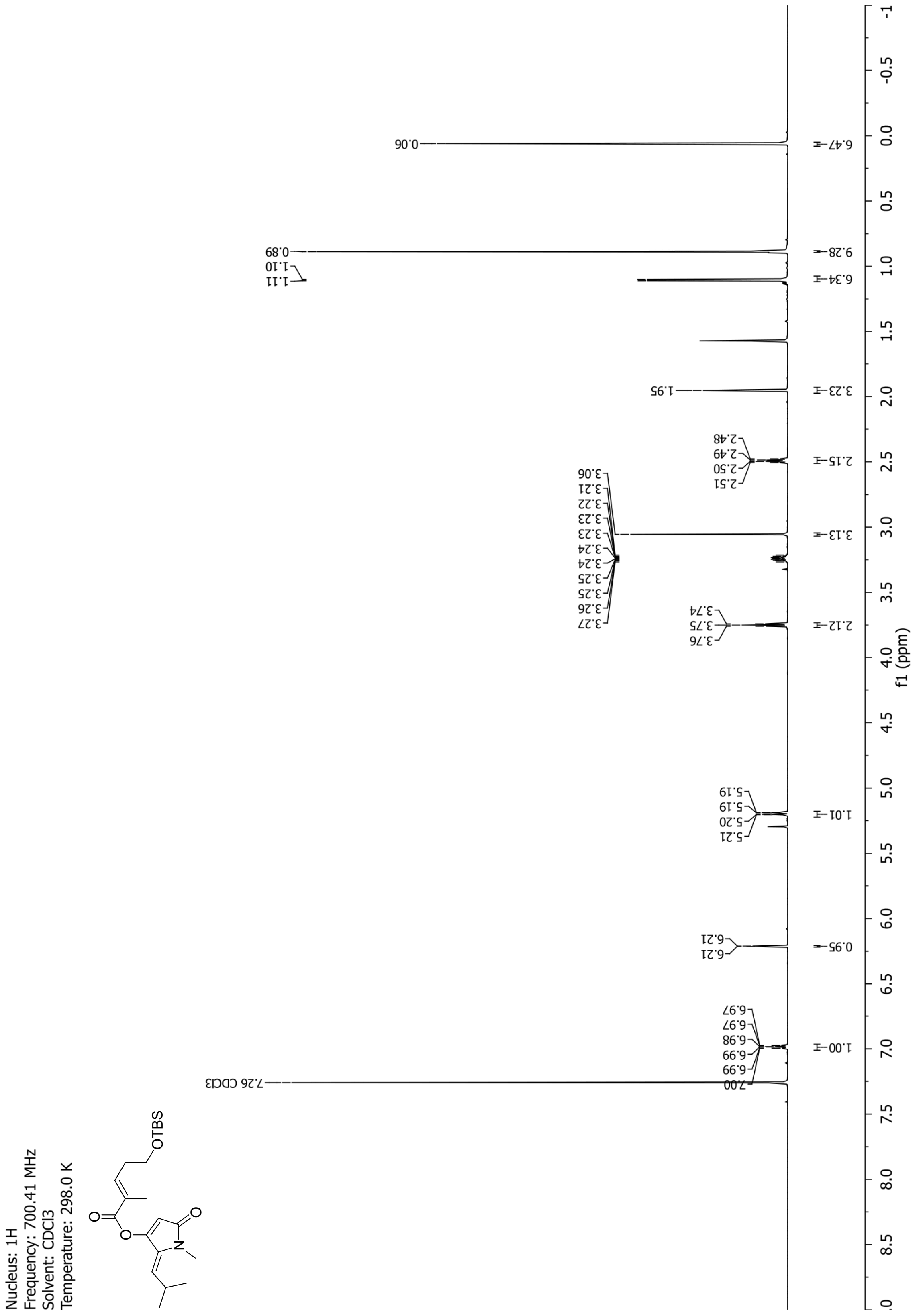

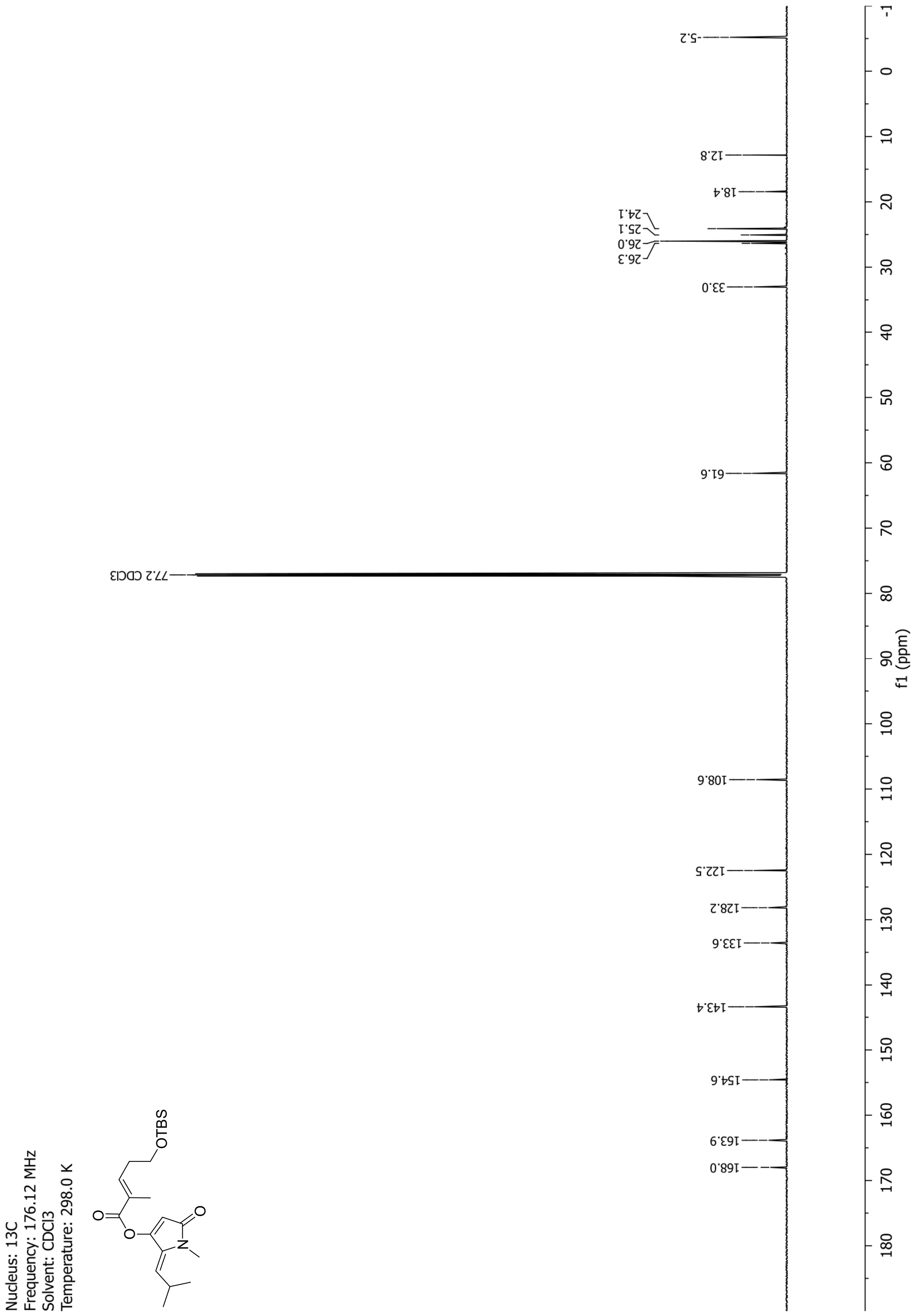
Nucleus: 13C
Frequency: 176.12 MHz
Solvent: CDCl3
Temperature: 298.0 K
OTBS
O
N
-5.2
12.8
18.4
24.1
25.1
26.0
26.3
33.0
61.6
77.2 CDCl3
108.6
122.5
128.2
133.6
143.4
154.6
163.9
168.0
f1 (ppm)

NMR-Spectra for Compound 13

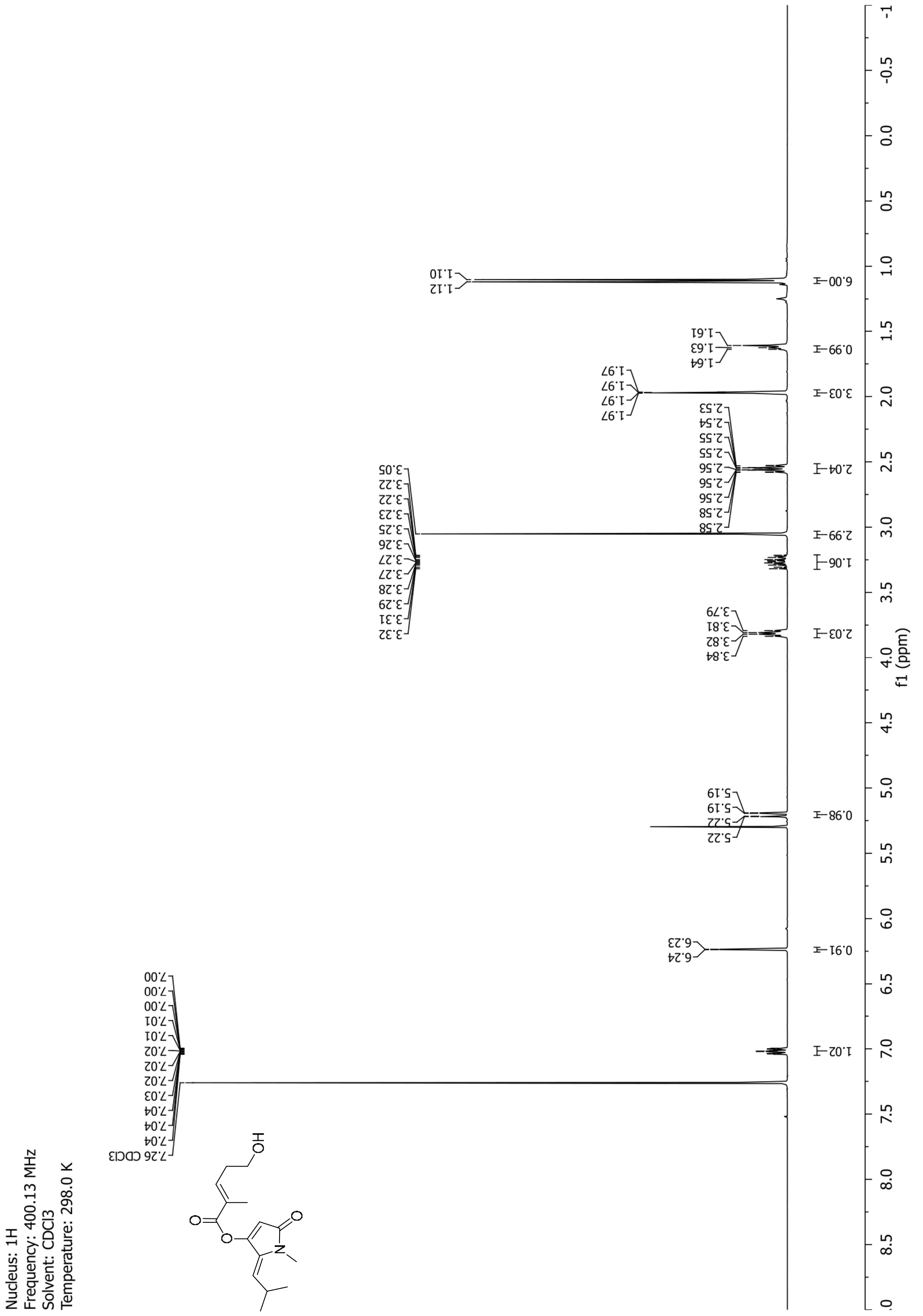

Nucleus: 13C
Frequency: 125.76 MHz
Solvent: CDCl3
Temperature: 298.0 K

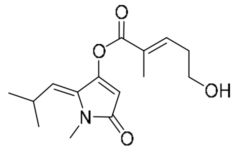

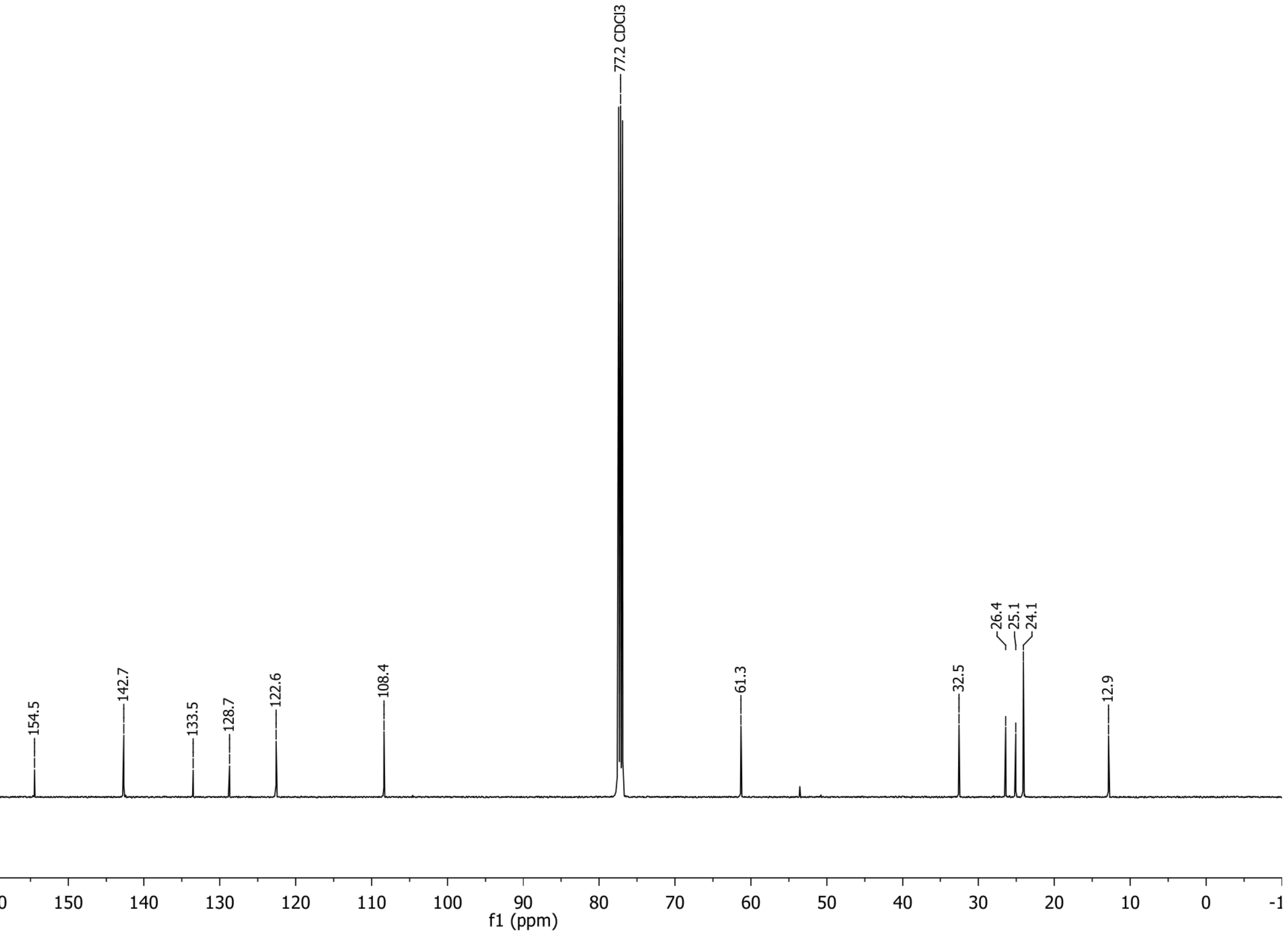

NMR-Spectra for Compound 14

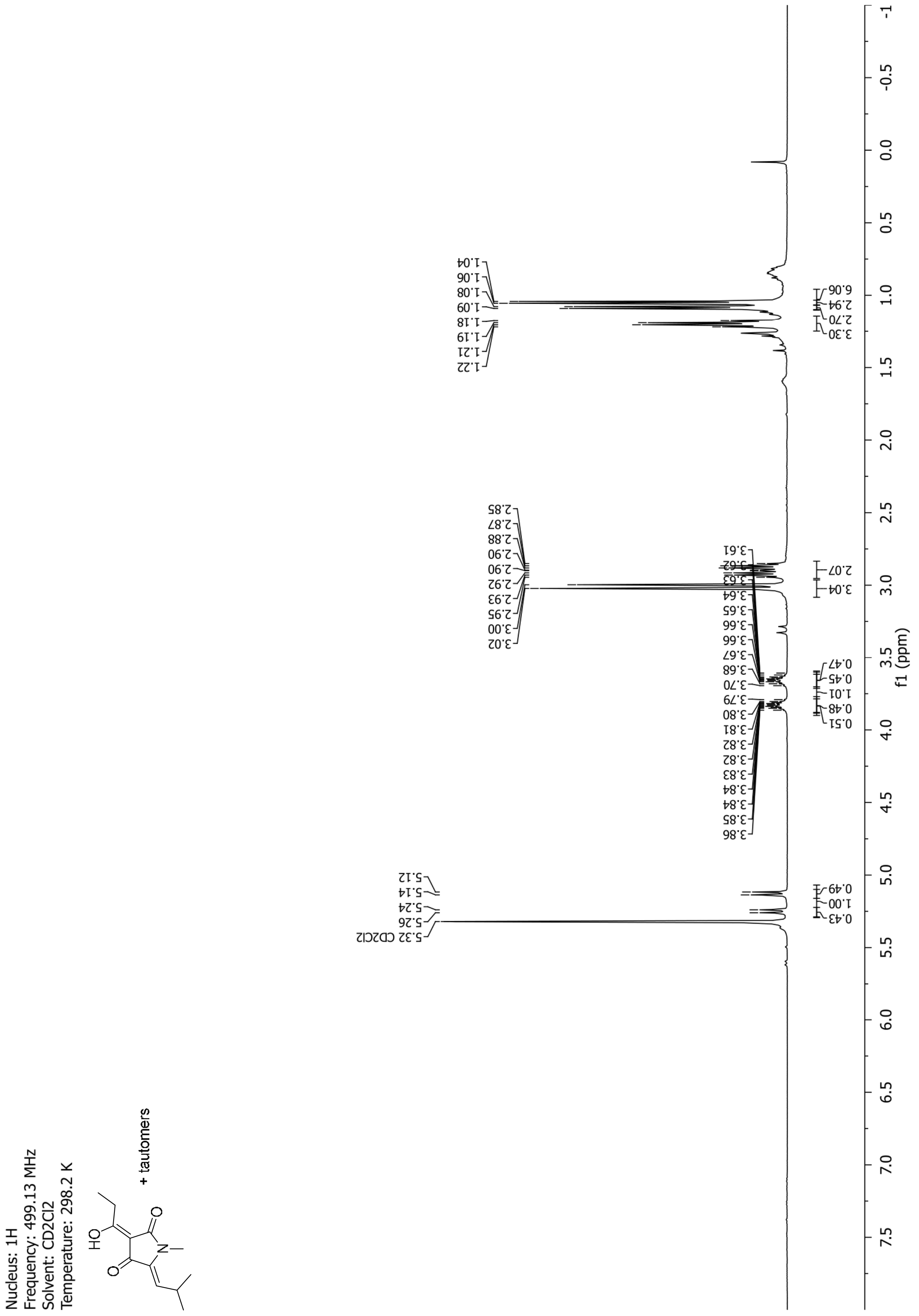

Nucleus: 13C
Frequency: 125.51 MHz
Solvent: CD2Cl2
Temperature: 298.1 K

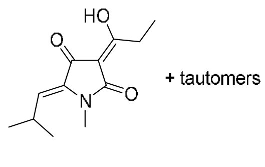

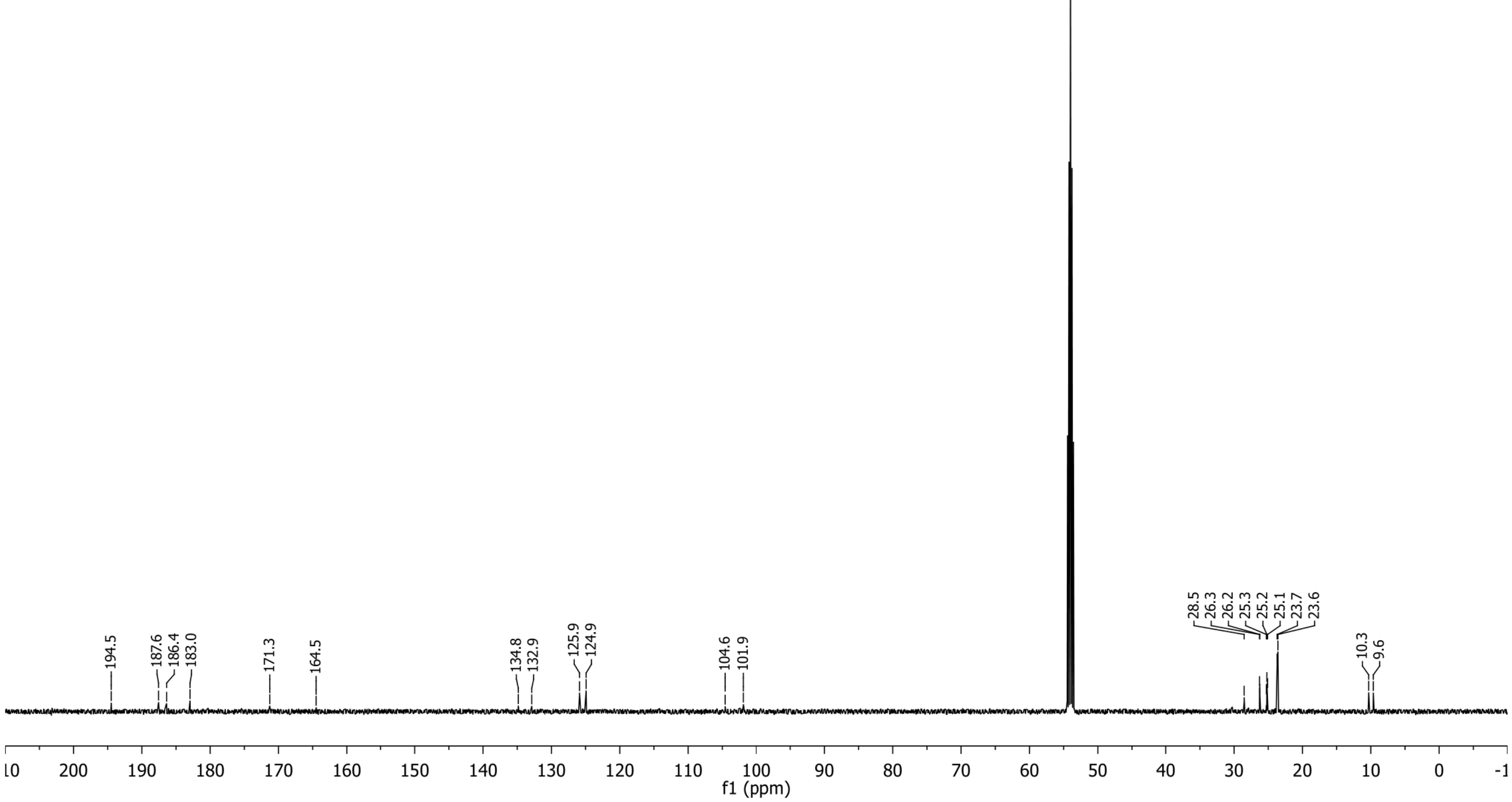

NMR-Spectra for Compound 15

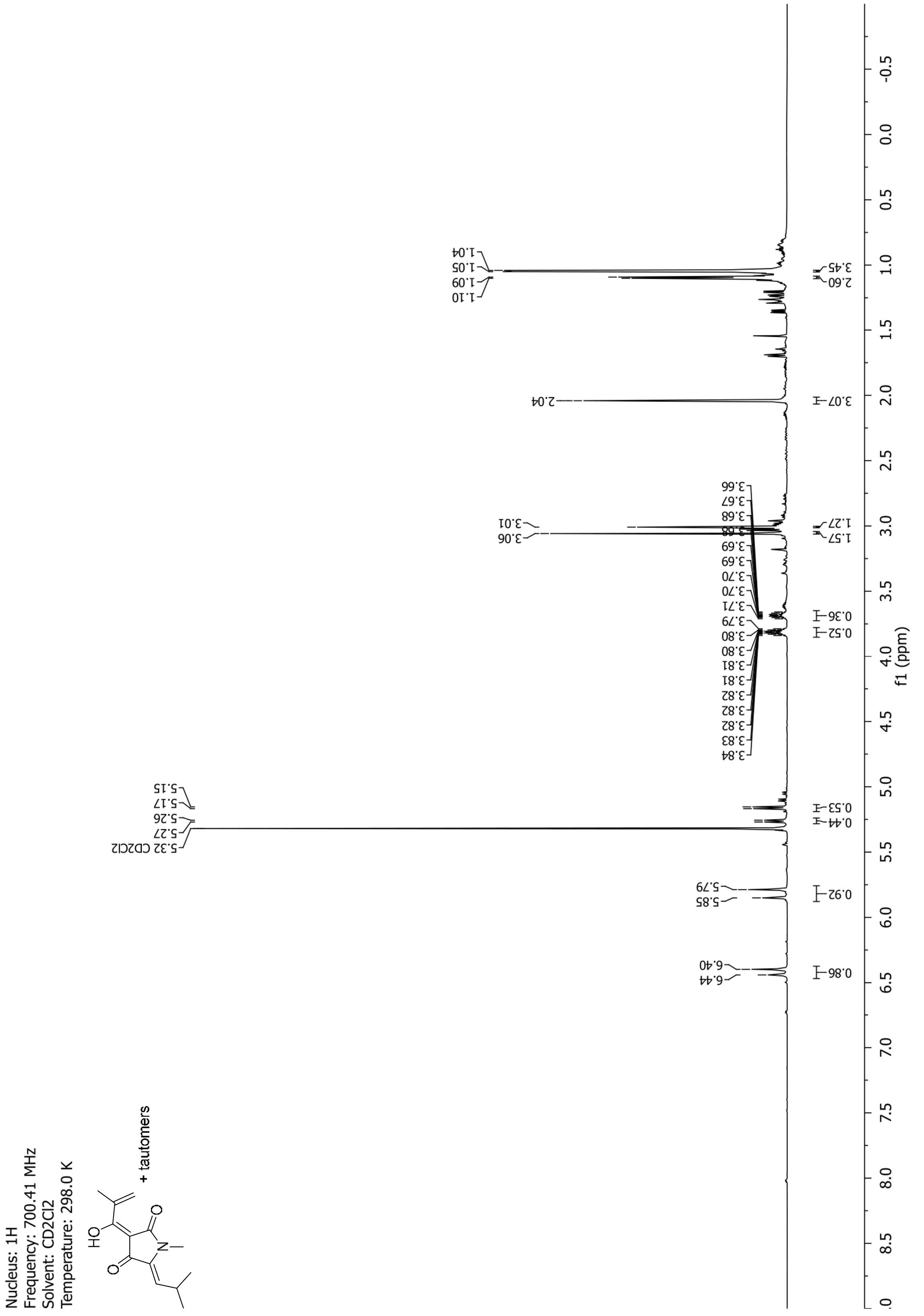

Nucleus: 13C
Frequency: 176.12 MHz
Solvent: CD2Cl2
Temperature: 298.0 K

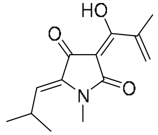

+ tautomers

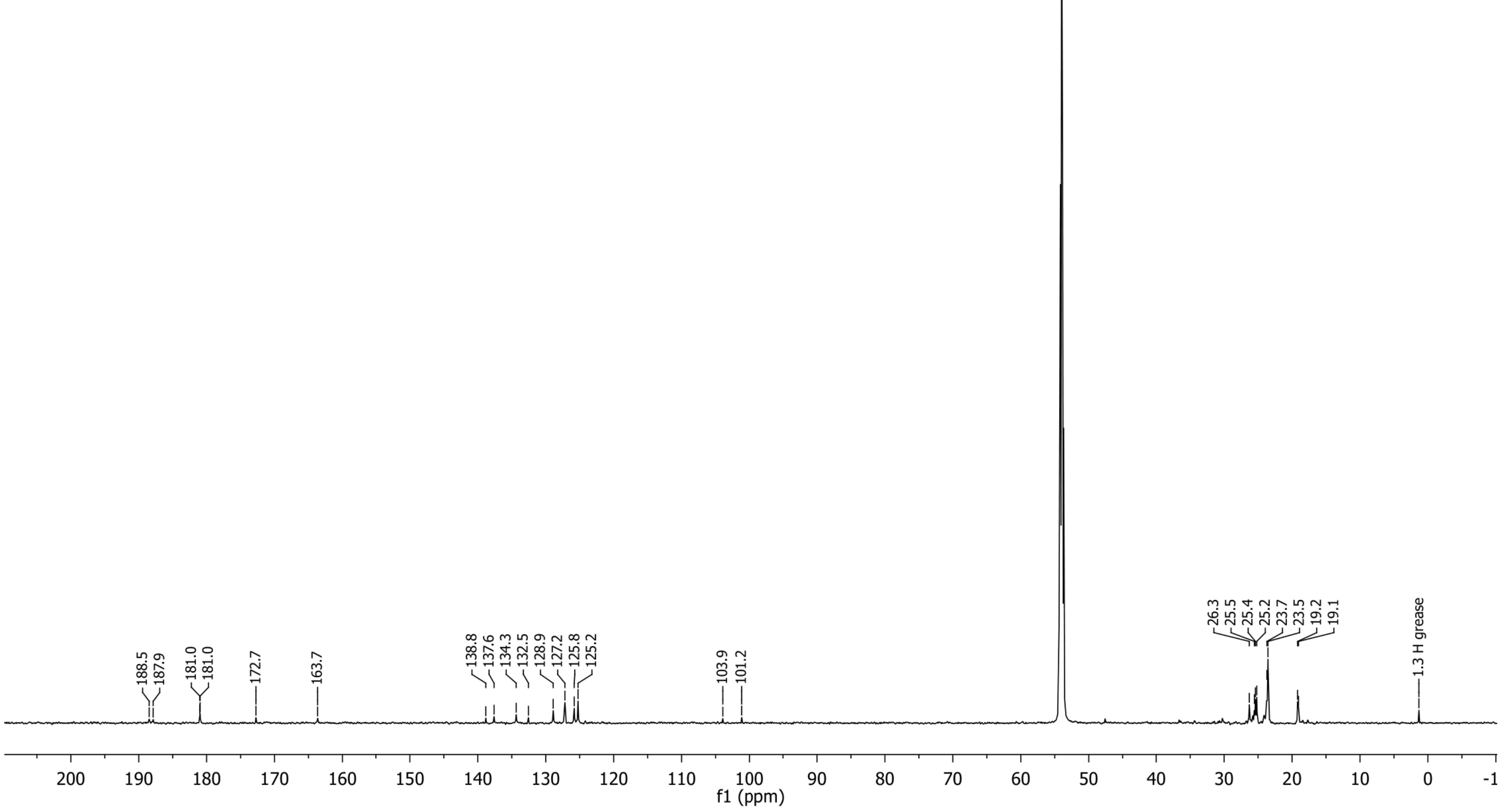

NMR-Spectra for Compound 16

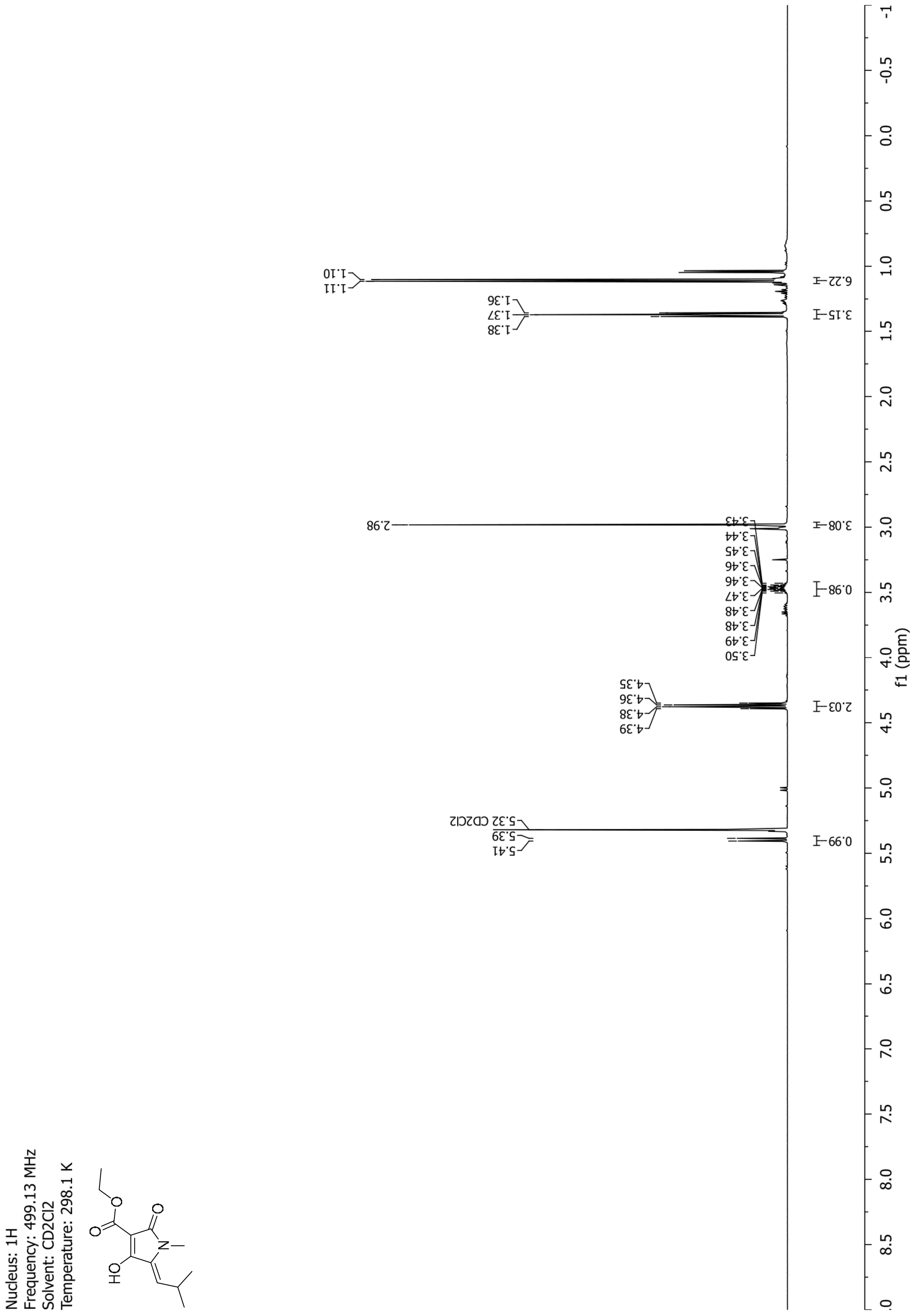

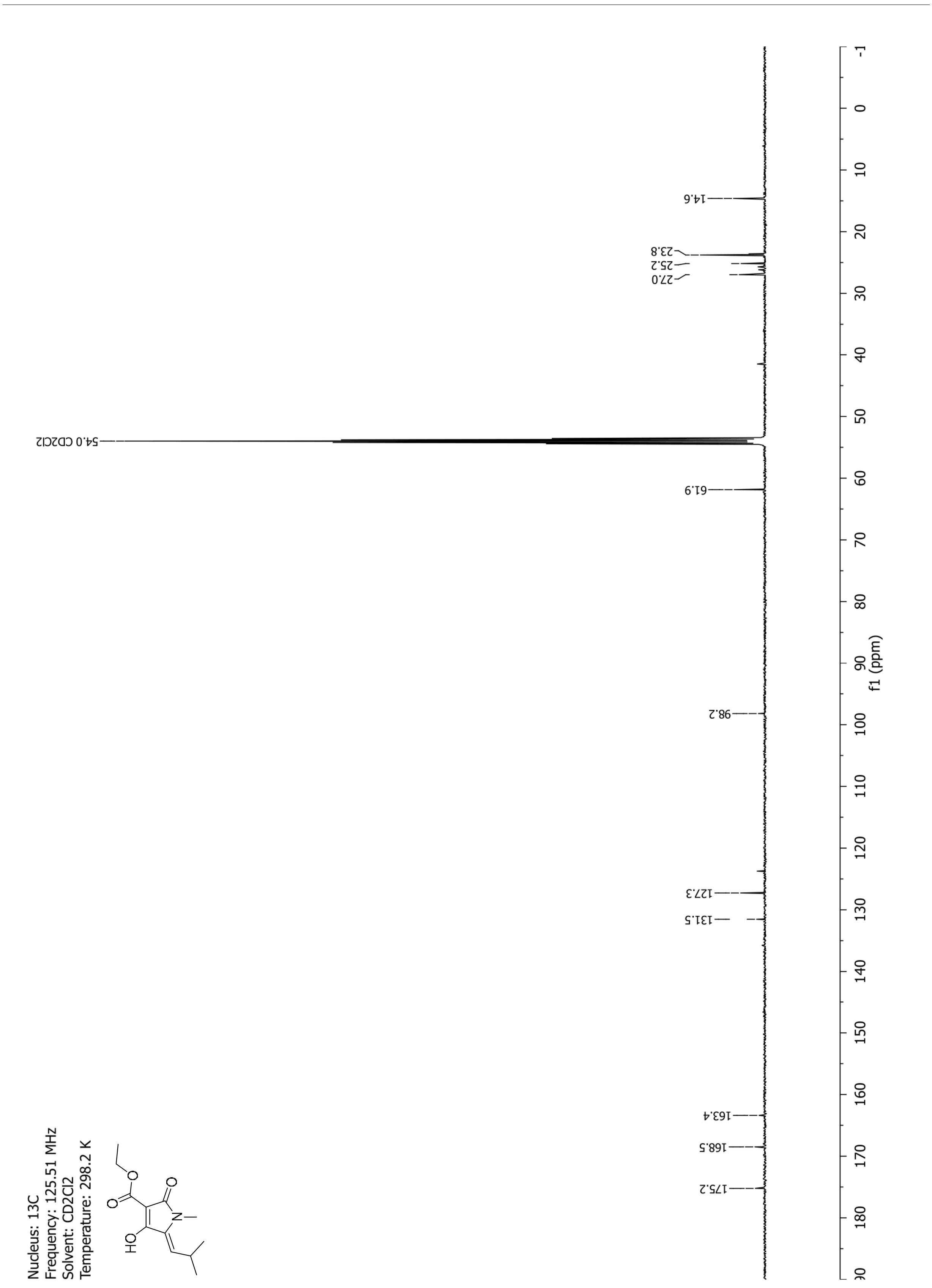

Nucleus: 13C
Frequency: 125.51 MHz
Solvent: CD2Cl2
Temperature: 298.2 K
HO
O
O
O
N
175.2
168.5
163.4
131.5
127.3
98.2
61.9
54.0 CD2Cl2
27.0
25.2
23.8
14.6
f1 (ppm)

NMR-Spectra for Compound 17

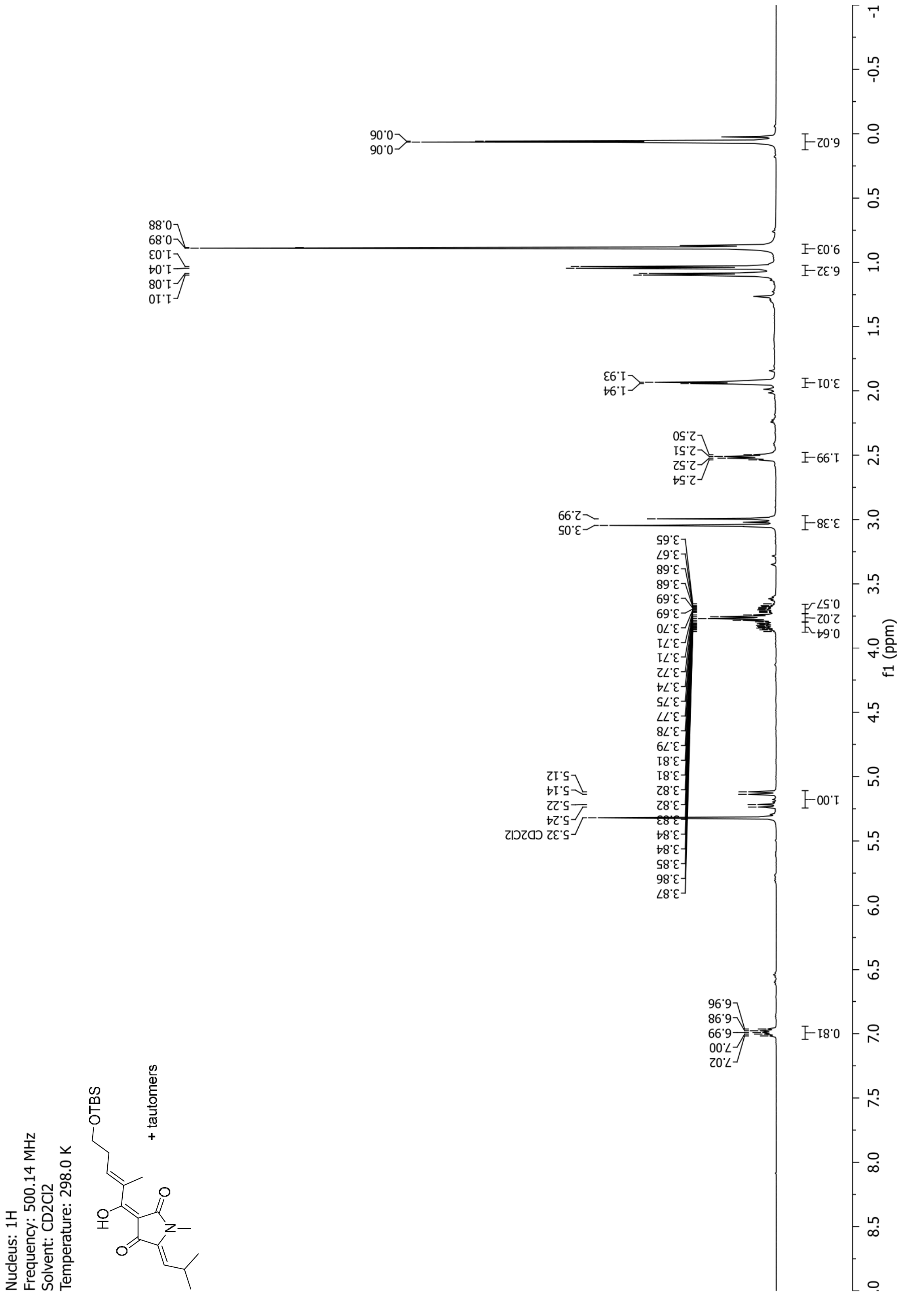

Nucleus: 13C
Frequency: 125.76 MHz
Solvent: CD2Cl2
Temperature: 298.0 K

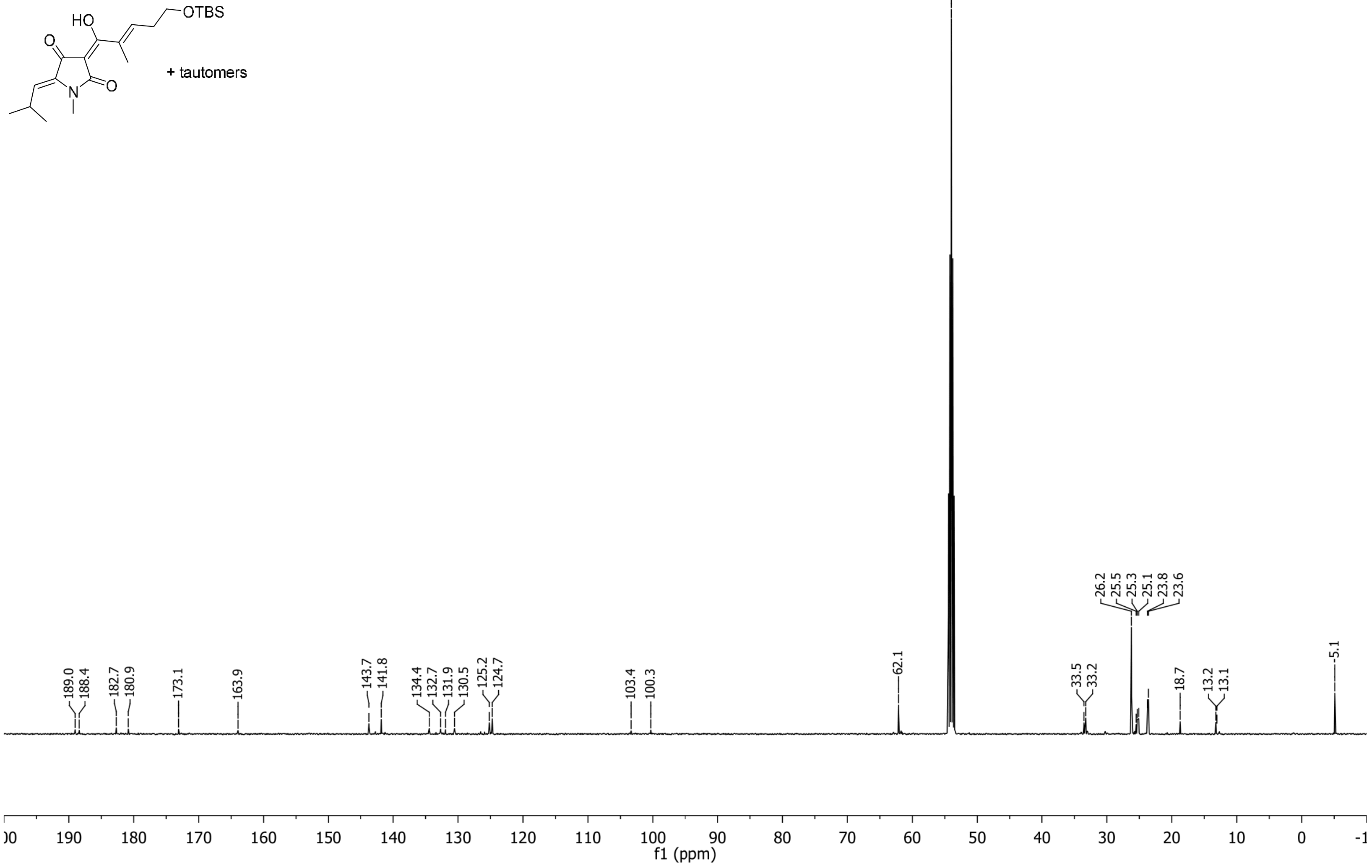

NMR-Spectra for Compound 18

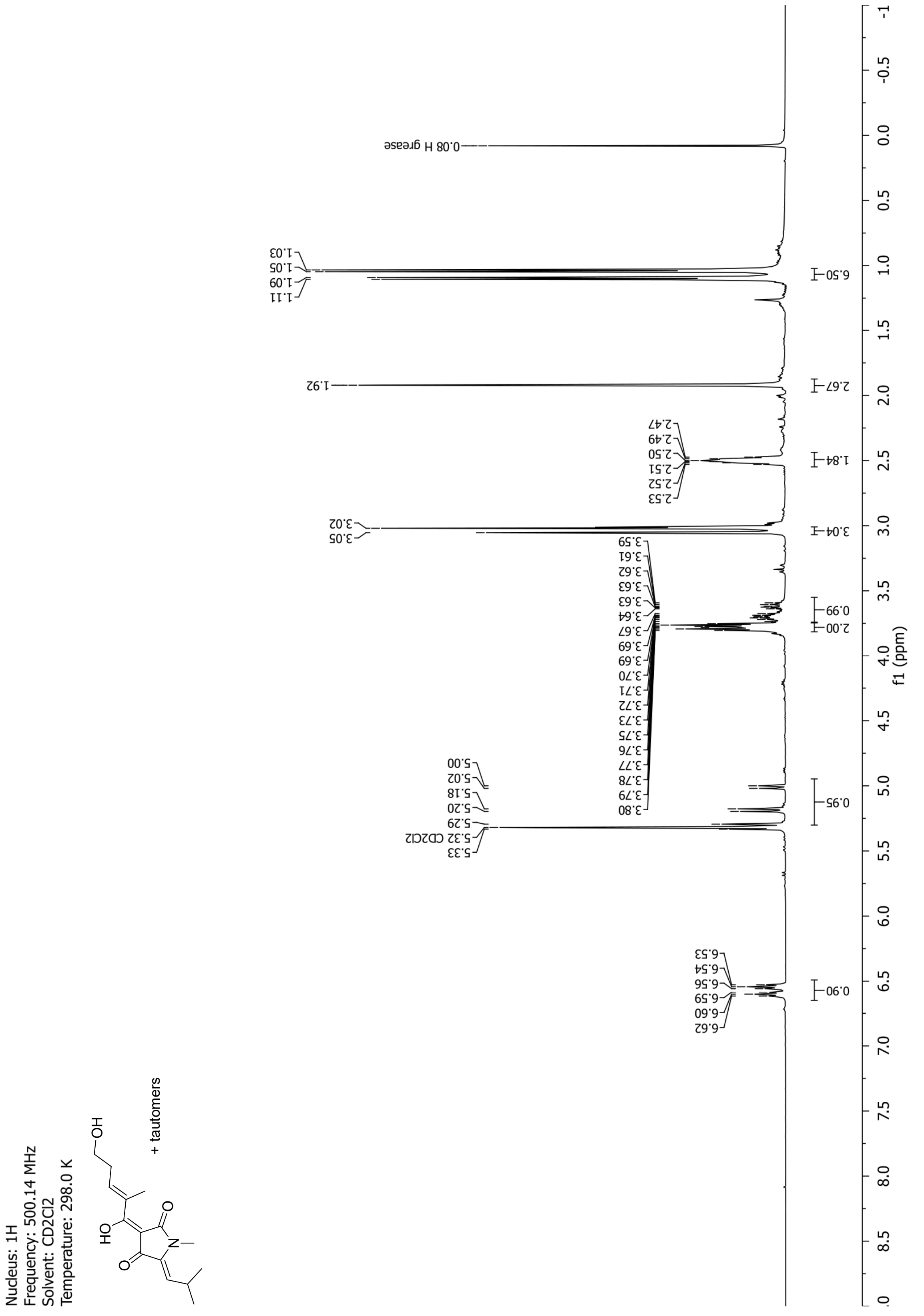

Nucleus: 13C
Frequency: 125.76 MHz
Solvent: CD2Cl2
Temperature: 298.0 K

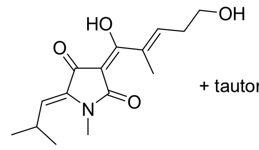

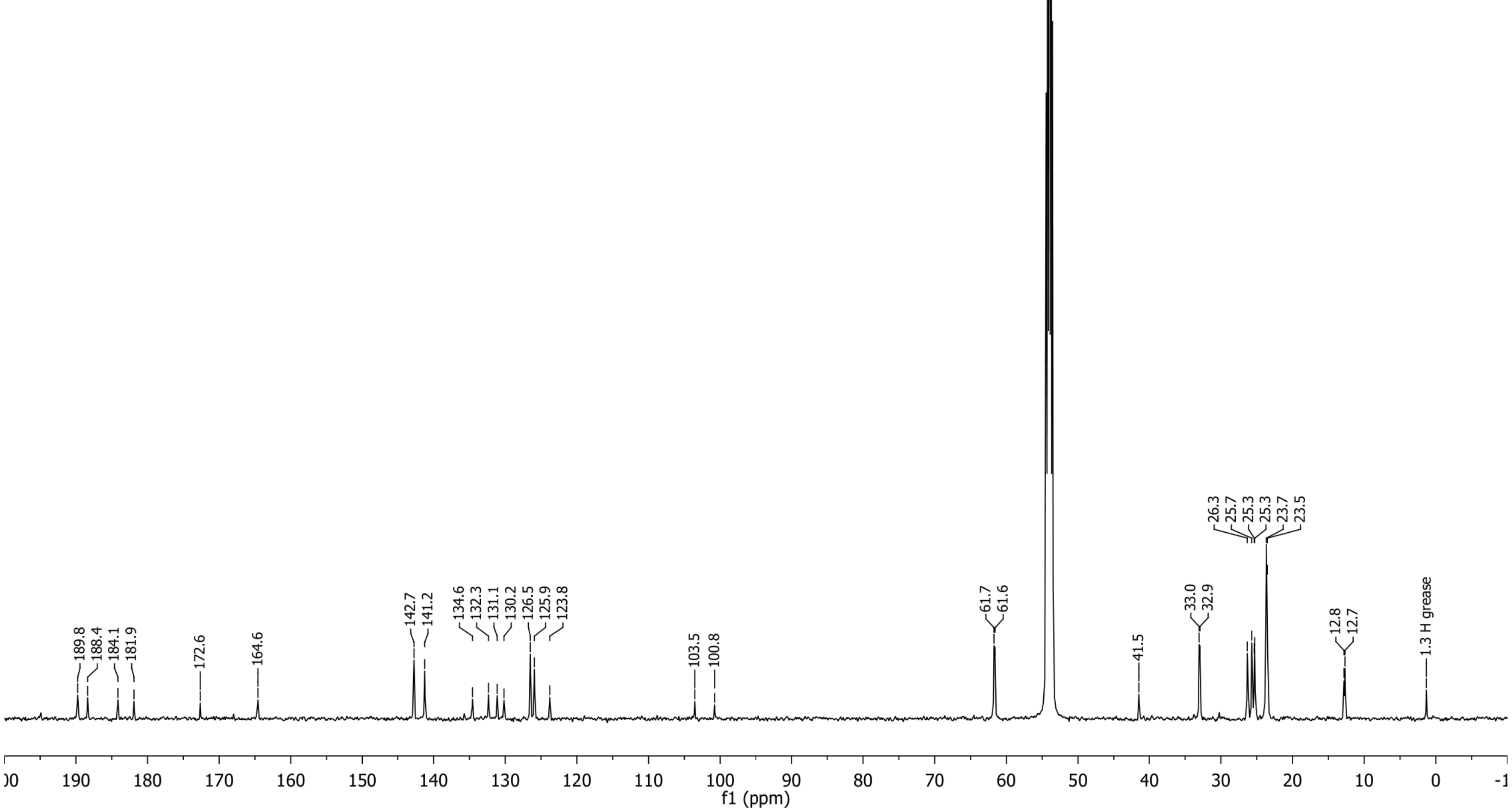

NMR-Spectra for Compound 19

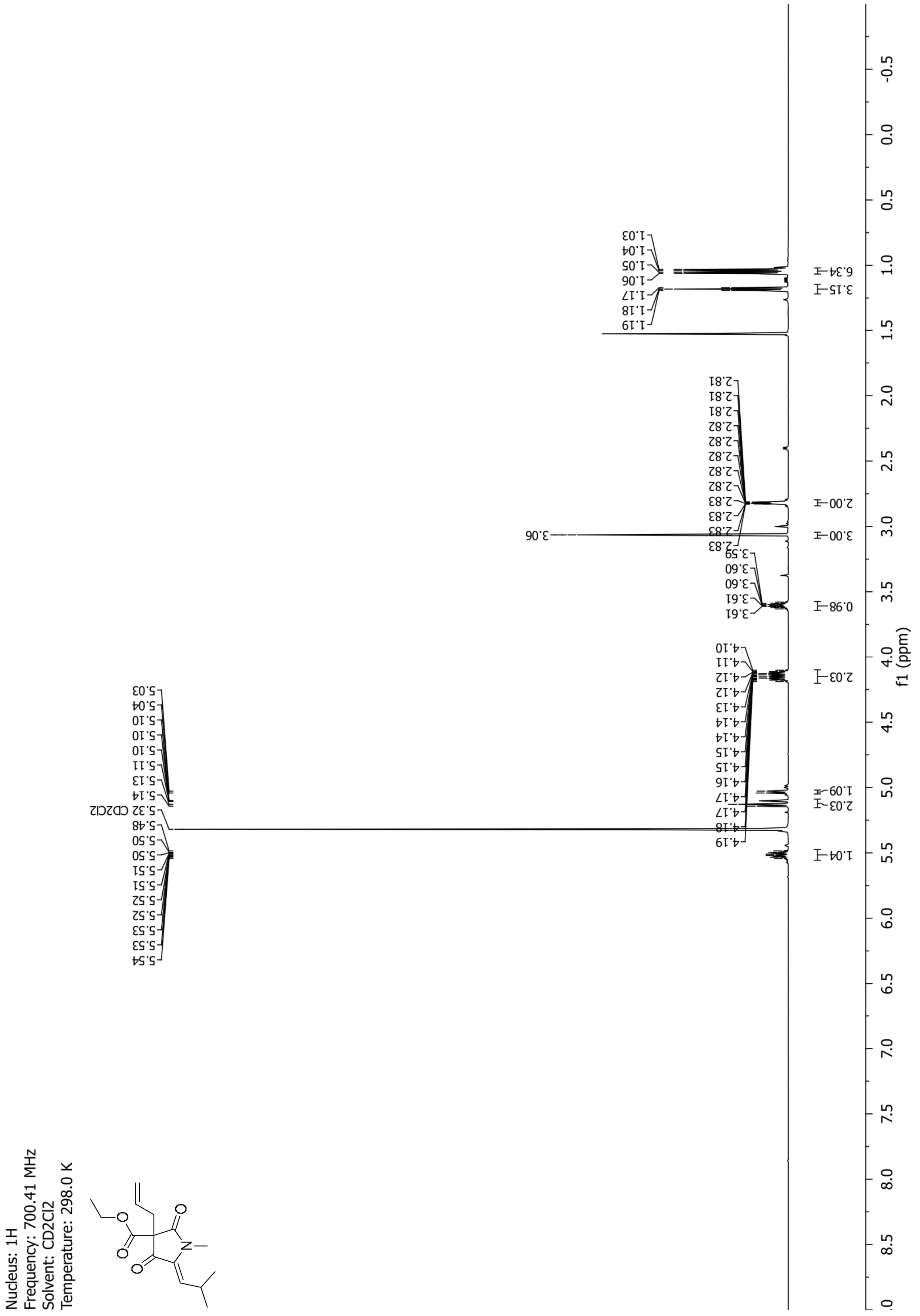

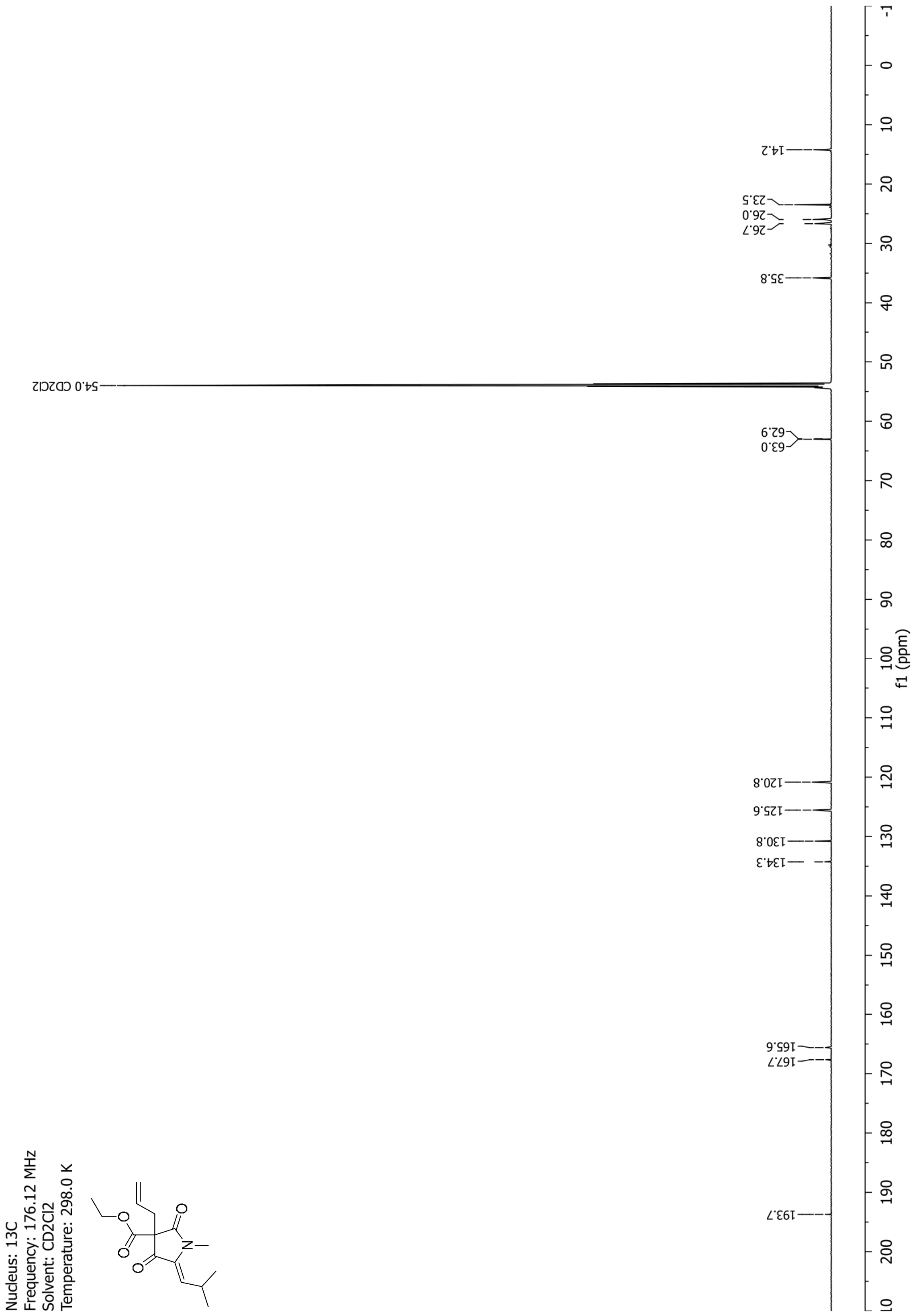
Nucleus: 13C
Frequency: 176.12 MHz
Solvent: CD2Cl2
Temperature: 298.0 K
193.7
167.7
165.6
134.3
130.8
125.6
120.8
63.0
62.9
54.0 CD2Cl2
35.8
26.7
26.0
23.5
14.2
f1 (ppm)
